# Impact of antiviral therapy on hepatocellular carcinoma and mortality in patients with chronic hepatitis C: systematic review and meta-analysis

**DOI:** 10.1186/s12876-017-0606-9

**Published:** 2017-04-04

**Authors:** Chang Seok Bang, Il Han Song

**Affiliations:** 1 Department of Internal Medicine, Hallym University College of Medicine, Chuncheon, Republic of Korea; 2 Division of Hepatology, Department of Internal Medicine, Dankook University College of Medicine, Cheonan, Korea Republic of Korea

**Keywords:** Antiviral therapy, Chronic hepatitis C, Hepatocellular carcinoma, Mortality, Sustained virologic response

## Abstract

**Background:**

The long-term clinical outcomes of antiviral therapy for patients with chronic hepatitis C are uncertain in terms of hepatitis C virus (HCV)-related morbidity and mortality according to the response to antiviral therapy. This study aimed to assess the impact of antiviral treatment on the development of HCC and mortality in patients with chronic HCV infection.

**Methods:**

A systematic review was conducted for studies that evaluated the antiviral efficacy for patients with chronic hepatitis C or assessed the development of HCC or mortality between SVR (sustained virologic response) and non-SVR patients. The methodological quality of the enrolled publications was evaluated using Risk of Bias table or Newcastle-Ottawa scale. Random-effect model meta-analyses and meta-regression were performed. Publication bias was assessed.

**Results:**

In total, 59 studies (4 RCTs, 15 prospective and 40 retrospective cohort studies) were included. Antiviral treatment was associated with reduced development of HCC (vs. no treatment; OR 0.392, 95% CI 0.275–0.557), and this effect was intensified when SVR was achieved (vs. no SVR, OR: 0.203, 95% CI 0.164–0.251). Antiviral treatment was associated with lower all-cause mortality (vs. no treatment; OR 0.380, 95% CI 0.295–0.489) and liver-specific mortality (OR 0.363, 95% CI 0.260–0.508). This rate was also intensified when SVR was achieved [all-cause mortality (vs. no SVR, OR 0.255, 95% CI 0.199–0.326), liver-specific mortality (OR 0.126, 95% CI 0.094–0.169)]. Sensitivity analyses revealed robust results, and a small study effect was minimal.

**Conclusions:**

In patients with chronic hepatitis C, antiviral therapy can reduce the development of HCC and mortality, especially when SVR is achieved.

**Electronic supplementary material:**

The online version of this article (doi:10.1186/s12876-017-0606-9) contains supplementary material, which is available to authorized users.

## Background

Antiviral treatment for chronic hepatitis C (CHC) aims to prevent hepatitis C virus (HCV)-related morbidity and mortality, including complications of liver fibrosis or cirrhosis and the development of hepatocellular carcinoma (HCC). Treatment reduces the degree of necroinflammation of the liver and induces regression of hepatic fibrosis [1]. Although direct-acting antivirals have recently emerged as a promising therapy, conventional interferon (IFN) or pegylated IFN (PegIFN) with or without ribavirin (RBV) has been used as the standard treatment for curing HCV.

A sustained virologic response (SVR) is the surrogate indicator for eradicating HCV and is considered to be “cure” [2]. SVR24 or SVR12, which is the state of undetectable HCV RNA in a sensitive assay with a lower limit of detection <50 IU/mL at week 24 or 12 after the end of treatment are accepted as an endpoint of treatment [3].

The evolution of CHC is slow, and there is no specific symptom before progression to liver fibrosis. Due to delayed diagnosis of HCV-related chronic liver disease such as chronic hepatitis or liver fibrosis, it is difficult to start an anitviral treatment in the early stage of the disease. Previous study has demonstrated an achievement of SVR was associated with less risk for mortality (risk ratio 0.16) and development of HCC (risk ratio 0.37) [4]. However, the majority of studies assessed short-term prognosis and the long-term clinical outcomes of antiviral therapy for patients with chronic hepatitis C are uncertain in terms of HCV-related morbidity and mortality, including disease progression to advanced hepatic fibrosis or cirrhosis, hepatic decompensation, HCC, and liver-specific death, especially according to the response to antiviral therapy. Moreover, viral replication of HCV is not known to be directly related to HCC development [4].

The aim of this study was to assess the impact of antiviral treatment on the development of HCC and mortality in patients with CHC.

## Methods

This systematic review and meta-analysis fully adhered to the principle of PRISMA (Preferred Reporting Items for Systematic reviews and Meta-Analyses) checklist.

### Literature searching strategy

PubMed, Embase, and the Cochrane Library were searched using common keywords associated with chronic hepatitis C, HCC, or SVR (from inception to April 2016) by 2 independent evaluators (C.S.B. and Y.J.Y.). Medical Subject Headings (MeSH) or Emtree keywords were selected for searching of electronic databases. The keywords included ‘hepatitis C’, ‘HCV’, ‘hepatocellular carcinoma’, ‘HCC’, ‘sustained virologic response’, ‘SVR’ and ‘mortality’. These keywords were combined for a searching strategy using Boolean operators. The abstracts of all identified studies were reviewed to exclude irrelevant articles. Full-text reviews were performed to determine whether the inclusion criteria were satisfied by the remaining studies and the bibliographies of relevant articles were reviewed to identify additional studies. Disagreements between the evaluators were resolved by discussion or consultation with a third evaluator (I.H.S.). The detailed searching strategy is described in Table 1.

**Table 1:** Clinical data of included studies

1. PubMed
1. Hepatitis C[Mesh] OR HCV 2. HCC OR “hepatocellular carcinoma” 3. SVR OR “sustained virologic response” 4. Mortality (#1 AND #2) OR (#1 AND #3) OR (#1 AND #4) - > removed duplicated articles
2. Embase
1. (hepatitis C or hcv).mp 2. (hcc or hepatocellular carcinoma).mp 3. (svr or sustained virologic reponse).mp 4. mortality After accumulation of (1 and 2), (1 and 3), and (1 and 4), and then removed duplicated articles
3. Cochrane library
1. Hepatitis C OR HCV 2. HCC OR “hepatocellular carcinoma” 3. SVR OR “sustained virologic response” 4. Mortality (#1 AND #2) OR (#1 AND #3) OR (#1 AND #4) - > removed duplicated articles

### Selection criteria

We included randomized or non-randomized studies that met the following criteria: 1. Study designed to evaluate the efficacy of antiviral treatment on the development of HCC or mortality in CHC patients and a control group, or in CHC patients with SVR and the no SVR group; 2. Publications on human subjects; 3. Full-text publication; and 4. English language. Studies that met the all of the inclusion criteria were sought and selected. The exclusion criteria were as follows: 1. Incomplete data; 2. Review article; 3. Animal study; 4. Letter or case article; or 5. Abstract only publication. Studies meeting at least 1 of the exclusion criteria were excluded from this analysis.

### Methodological quality

The methodological quality of the enrolled publications was assessed using the Risk of Bias table for randomized studies and the Newcastle-Ottawa Scale for non-randomized studies. The Risk of Bias was assessed as described in the Cochrane handbook by recording the method used to generate the randomization sequence, allocation concealment, determination of whether blinding was implemented for participants or staff, and evidence of selective reporting of the outcomes [5]. Review Manager version 5.3.3 (Revman for Windows 7, the Nordic Cochrane Centre, Copenhagen, Denmark) was used to generate the Risk of Bias table. The Newcastle-Ottawa scale is categorized into three parameters: the selection of the study population, the comparability of the groups, and the ascertainment of the exposure or outcome. Each parameter consists of subcategorized questions: selection (*n* = 4), comparability (*n* = 1), and exposure or outcome (*n* = 3) [6, 7]. Stars that are awarded for each item serve as a quick visual assessment of the methodological quality of the studies. A study can be graded a maximum of 9 stars, which indicates the highest quality. Two of the evaluators (C.S.B. and Y.J.Y.) independently assessed the methodological quality of all studies, and any disagreements between the evaluators were resolved by discussion or consultation with a third evaluator (I.H.S.).

### Primary and modifier-based analyses

The following questions were primary topic of this meta-analyses: In patients with CHC, 1. Does the antiviral treatment reduce the development of HCC? 2. Does the antiviral treatment reduce all-cause or 3. liver-specific mortality? 4. Does the achievement of SVR reduce the development of HCC? 5. Does the achievement of SVR reduce all-cause or 6. liver-specific mortality?

The analysis was performed as 6 distinct meta-analyses to answer the 6 questions described above. Two evaluators (C.S.B. and Y.J.Y.) independently used the same data fill-up form to collect the primary summary outcome and modifiers in each study. The outcome was the relative rate of the development of HCC or mortality between antiviral treatment and the control groups, or the SVR and no SVR groups. These ratios were extracted and evaluated by odds ratios (ORs). Sensitivity analyses, including cumulative and one study removed analyses were performed to confirm the robustness of the main analysis results. These analyses were calculated in the order of publication year or effect size to find whether the time trend exists or which study is more or less influential in the pooled estimate. We also performed a meta-ANOVA and meta-regression to identify the reason of heterogeneity based on the multiple modifiers identified during systematic review. These reasons include study format (randomized/prospective cohort/retrospective cohort study), nationality, histology (degree of liver fibrosis), follow-up duration, Newcastle-Ottawa scale, age, and the regimen of the treatment (IFN, IFN with RBV, PegIFN with or without RBV). The follow-up duration of each study was categorized as long-term (≥5 years) or short-term (<5 years).

### Statistics

Comprehensive Meta-Analysis software (version 3, Biostat; Borenstein M, Hedges L, Higgins J and Rothstein H. Englewood, NJ, USA) was used for this meta-analysis. We calculated the ORs with 95% confidence intervals (CIs) using 2 × 2 tables from the original articles to evaluate the efficacy of antiviral treatment between the treatment and control groups, or the SVR and no SVR groups whenever possible. Heterogeneity was determined using the *I* ^*2*^ test developed by Higgins, which measures the percentage of total variation across studies [8]. *I* ^*2*^ was calculated as follows: *I* ^*2*^ (%) = 100 × (Q-df)/Q, where Q is Cochrane’s heterogeneity statistic and df signifies the degree of freedom. Negative values for *I* ^*2*^ were set to zero, and an *I* ^*2*^ value over 50% was considered to be of substantial heterogeneity (range: 0–100%) [9]. Pooled-effect sizes with 95% CIs were calculated using a random effects model and the method of DerSimonian and Laird due to methodological heterogeneity [10]. These results were confirmed by the *I* ^*2*^ test. Significance was set at *p* = 0.05. Publication bias was evaluated using Begg’s funnel plot, Egger’s test of the intercept, Begg and Mazumdar’s rank correlation test, and Duval and Tweedie’s trim and fill method [11–15].

## Results

### Identification of relevant studies

**Fig. 1:**
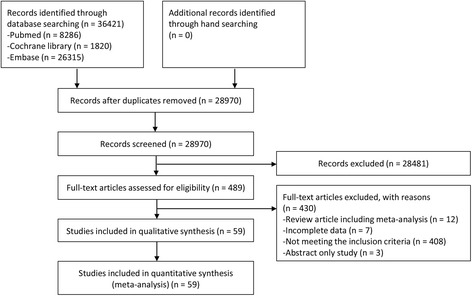
Flow diagram for identification of relevant studies

Figure 1 presents a flow diagram of how relevant studies were identified. In total, 36,421 articles were identified by a search of 3 databases. In all, 7451 duplicate studies and an additional 28,481 studies were excluded during the initial screening through a review of the titles and abstracts. The full texts of the remaining 489 studies were then thoroughly reviewed. Among these studies, 431 articles were excluded from the final analysis. The reasons for study exclusion during the final review were as follows: review article (*n* = 12), incomplete data (*n* = 7), not meeting the inclusion criteria (*n* = 409), or abstract only study (*n* = 3). The remaining 58 studies [4 randomized controlled studies (RCTs), 15 prospective cohort, and 40 retrospective cohort studies] were included in the final analysis.

### Characteristics of included studies

In each study topic, about 13–35 studies were enrolled. In terms of the study format, RCTs, prospective and retrospective cohort studies were mixed. The number of Western population-based studies and the number of Asian population-based studies were evenly distributed. The age of enrolled patients ranged from 37 to 64 years (median). The follow-up duration ranged from 32 months (mean) to 11.5 years (median). Most of the studies used IFN-based regimens with or without RBV in topic 1, 2 and 3. However, a PegIFN-based regimen and IFN-based regimens were evenly distributed in topic 4, 5, and 6. Underlying histology of liver was variable, but some studies exclusively assessing liver cirrhosis patients were included. The detailed characteristics of the included studies are described in Tables 2, 3, 4, 5, and 6.

**Table 2:** Clinical data summary of all included studies

Topic	Number of enrolled studies and population	Study format	Nationality	Age	Follow-up duration	Treatment regimen	Histology
Topic 1	25 studies (9691 treated vs. 6010 control)	3 RCTs 8 prospective cohort studies 14 retrospective cohort studies	15 Western population-based studies 10 Asian population-based studies	37 to 61 years (median)	32 months to 10 years (mean)	IFN-based regimens with or without RBV, except 4 studies with a PegIFN-based regimen	10 studies exclusively assessing LC patients)
Topic 2	17 studies (9868 treated vs. 4700 controls)	1 RCT 5 prospective cohort studies 11 retrospective cohort studies	8 Western population-based studies 9 Asian population-based studies	37 to 61 years (median)	55 months to 11.5 years (median)	IFN-based regimen with or without RBV, except 3 studies with a PegIFN-based regimen	3 studies exclusively assessing LC patients
Topic 3	13 studies (8671 treated vs. 2831 controls)	5 prospective cohort studies 8 retrospective cohort studies	5 Western population-based studies 8 Asian population-based studies	37 to 61 years (median)	55 months to 11.5 years (median)	IFN-based regimen with or without RBV, except 2 studies with a PegIFN-based regimen	4 studies exclusively assessing LC patients
Topic 4	35 studies (14756 patients with SVR vs. 12741 patients with no SVR)	1 RCT 8 prospective cohort studies 26 retrospective cohort studies	17 Western population-based studies 17 Asian population-based studies 1 Saudi Arabia and Egypt population-based study	37 to 64 years (median)	2.1 (median) to 10 years (mean)	20 studies with PegIFN-based regimen 15 studies with IFN-based regimen	9 studies exclusively assessing LC patients
Topic 5	22 studies (12440 patients with SVR vs. 18980 patients with no SVR)	4 prospective cohort studies 18 retrospective cohort studies	12 Western population-based studies 9 Asian population-based studies 1 Saudi Arabia and Egypt population-based study	41.8 to 64 years (mean)	2.1 to 11.5 years (median)	11 studies with PegIFN-based regimen 11 studies with IFN-based regimen	3 studies exclusively assessing LC patients
Topic 6	23 studies (5148 patients with SVR vs. 10356 patients with no SVR)	7 prospective cohort studies 16 retrospective cohort studies	14 Western population-based studies 9 Asian population-based studies	41.8 to 64 (mean)	2.1 to 11.5 years (median)	12 studies with PegIFN-based regimen 11 studies with IFN-based regimen	6 studies exclusively assessing LC patients

*RCT* randomized controlled study, *IFN* interferon, *PegIFN* pegylated interferon, *RBV* ribavirin, *LC* liver cirrhosis, *SVR* sustained virologic response

**Table 3:** Clinical data of included studies for the efficacy of antiviral treatment on the development of HCC in patients with CHC

Study	Nationality	Age	Duration of follow up	Study format	Genotype	NOS	Treatment	HCC/Total treatment	HCC/Control	Histology
Mazella G et al. (1996) [23]	Italy	Tx: 53, control: 54 (mean)	mean 32 months	P	unknown	7	IFN-α or lymphoblastoid	5/193	9/92	Child A LC
Bruno S et al.(1997) [24]	Italy	Tx 56, control: 59 (mean)	median 68 months	P	62% type 1b	7	IFN-α	6/83	16/80	LC (mainly Child A)
Fattovich G et al. (1997) [25]	Italy	Tx: 53, control: 57 (mean)	mean 60 months	R	unknown	8	IFN-α	7/193	16/136	LC
Serfaty L et al. (1998) [26]	France	Tx: 55, control: 56 (mean)	median 40 months	P	48% 1b	7	IFN-α	2/59	9/44	Knodell 10 (mean)
Benvegnù L et al.(1998) [27]	Italy	Tx: 56.7, control: 59.5 (mean)	mean 71.5 months	P	unknown	8	IFN	4/75	20/77	Child A LC
International Interferon-α Hepatocellular Carcinoma Study Group (1998) [28]	Italy and Argentina	54 (median)	36 months	R	unknown	7	IFN-α or lymphoblastoid	21/232	48/259	unknown
Imai Y et al.(1998) [29]	Japan	unknown	Tx: 47.6, control: 46.8 (median)	R	unknown	7	IFN-α	28/419	19/144	F3,4: 37% in Tx, 53% in control
Yoshida H et al. (1999) [30]	Japan	Tx: 49.5, control: 53.6 (mean)	median 4.3 years	R	70.3% type 1	7	IFN-α or IFN-β or combination	89/2400	59/490	F3,4: 33.1% in Tx, 33.8% in control
Okanoue T et al. (1999) [31]	Japan	42.6–57.6 (mean)	mean 39.5–67.1 months	R	unknown	7	IFN-α or lymphoblastoid	52/1148	22/55	F3,4: 34% in Tx, F4: 100% in control
Valla DC et al. (1999) [32]	France	Tx: 57, control: 56 (mean)	mean 160 weeks	RCT	unknown		IFN-α	5/47	9/52	compensated LC
Ikeda K et al. (2001) [33]	Japan	57 (median)	median 7.6 years	R	unknown	7	IFN-α or IFN-β	32/113	271/581	LC
Gramenzi A et al. (2001) [34]	Italy	Tx: 57.9, control: 58.1 (mean)	median 55–58 months	P	unknown	7	IFN-α	6/72	19/72	LC (mainly Child A)
Nishiguchi S et al. (2001) [35]	Japan	Tx: 54.7, control: 57.3 (mean)	mean 8.2 years	RCT	75.6% type 2		IFN-α	12/45	33/45	unknown
Testino G et al. (2002) [16]	Italy	Tx: 55.3, control: 56.8 (mean)	mean 95.4 months	R	55% type 1b, 45% type 2	8	IFN-α	12/51	24/71	Child A LC
Coverdale SA et al. (2004) [36]	Australia	Tx: 37, control: 38 (median)	median 9 years	P	39.6% type 1	7	IFN-α	26/384	7/71	Scheuer fibrosis score 2
Azzaroli F et al. (2004) [37]	Italy	55.1 (mean)	5 years	RCT	64.4% type 1b		IFN-α with RBV	2/71	9/30	LC
Shiratori Y et al. (2005) [38]	Japan	Tx: 57, control: 61 (median)	median 6.8 years	P	71.9% type 1b	8	IFN-α or lymphoblastoid	84/271	35/74	unknown
Yu ML et al. (2006) [39]	Taiwan	Tx: 46.9, control: 43.6 (mean)	mean 5.18–5.15 years	R	46.2% type 1	8	IFN-α with or without RBV	51/1057	54/562	LC 15.6% in Tx, 12.1% in control
Sinn DH et al. (2008) [40]	Korea	48.4–58.2 (mean)	median 55.2 months	R	48.6% type 2	7	IFN/PegIFN with or without RBV	14/490	122/647	F3,4: 49% in Tx, F4: 33% in control
Di Martino V et al. (2011) [41]	France	unknown	median 59 months	R	57.9% type 1	7	IFN with or without RBV, or PegIFN with RBV	9/184	5/184	55.5% F2 or greater
Tateyama M et al. (2011) [42]	Japan	57 (median)	mean 8.2 years	R	72.1% type 1b	8	IFN/PegIFN with or without RBV	110/373	63/334	F3,4: 34.1%
Maruoka D et al. (2012) [43]	Japan	50.4–54 (mean)	mean 9.9 years	R	73.6% type 1	8	IFN-α/IFN-β with or without RBV	85/577	35/144	F3,4: 24.3% in Tx, F4: 43.1% in control
Cozen ML et al. (2013) [44]	US	50.98 (mean)	mean 10 years	R	68.7% type 1	8	IFN-α with or without RBV	11/159	9/199	F3,4: 19% (30.2% in Tx, 10.1% in control)
Aleman S et al. (2013) [45]	Sweden	51 (mean)	mean 5.3 years	R	50% type 1	8	PegIFN with RBV	32/303	14/48	LC
Cozen ML et al. (2016) [46]	US	51.4 (mean)	mean 8.5 years	P	71.6% type 1 or 4	8	IFN-α with RBV	43/692	84/1519	LC 15.8% in Tx, 5.3% in control

*HCC* hepatocellular carcinoma, *CHC* chronic hepatitis C, *NOS* Newcastle-Ottawa scale, *Tx* treatment group, *R* retrospective cohort study, *P* prospective cohort study, *RCT* randomized controlled study, *IFN* interferon, *PegIFN* pegylated interferon, *RBV* ribavirin, *LC* liver cirrhosis

**Table 4:** Clinical data of included studies for the efficacy of antiviral treatment on all-cause and liver-specific mortality in patients with CHC

Study	Nationality	Age	Duration of follow up	Study format	Genotype	NOS	Treatment	Death/Total treatment	Death/Control	Histology
Benvegnù L et al. (1998) [27]	Italy	Tx: 56.7, control: 59.5 (mean)	mean 71.5 months	P	unknown	8	IFN	^a^3/75	^a^15/77	Child A LC
Ikeda K et al. (2001) [33]	Japan	57 (median)	median 7.6 years	R	unknown	7	IFN-α or IFN-β	20/113 ^a^12/113	266/581 ^a^124/581	LC
Gramenzi A et al. (2001) [34]	Italy	Tx: 57.9, control: 58.1 (mean)	median 55–58 months	P	unknown	7	IFN-α	^a^7/72	9/72 ^a^8/72	LC (mainly Child A)
Nishiguchi S et al. (2001) [35]	Japan	Tx: 54.7, control: 57.3 (mean)	mean 8.2 years	RCT	75.6% type 2		IFN-α	5/45	26/45	unknown
Testino G et al. (2002) [16]	Italy	Tx: 55.3, control: 56.8 (mean)	mean 95.4 months	R	55% type 1b, 45% type 2	8	IFN-α	1/51	9/71	Child A LC
Yosida H et al. (2002) [47]	Japan	Tx: 49.5, control: 54.6 (mean)	mean 5.4 years	R	unknown	8	IFN-α or IFN-β	56/2430 ^a^35/2430	30/459 ^a^23/459	F3,4: 32.2% in Tx, 31.6% in control, 26.3% in SVR, 35.2% in no SVR
Imazeki F et al. (2003) [48]	Japan	Tx: 49.2, control: 53.1 (mean)	mean 8.2 years	R	73.9% type 1	8	IFN-α or IFN-β	33/355 ^a^19/355	15/104 ^a^12/104	F3,4: 26.7% in Tx, 29.8% in control,
Coverdale SA et al. (2004) [36]	Australia	Tx: 37, control: 38 (median)	median 9 years	P	39.6% type 1	7	IFN-α	^a^36/384	^a^12/71	Scheuer fibrosis score 2
Kasahara A et al. (2004) [17]	Japan	Tx: 53, control: 54 (median)	mean 6 years	R	unknown	8	IFN	101/2698 ^a^69/2698	52/256 ^a^42/256	F3,4: 38.7% in Tx, 48% in control, 28.6% in SVR, 43% in no SVR
Shiratori Y et al. (2005) [38]	Japan	Tx: 57, control: 61 (median)	median 6.8 years	P	71.9% type 1b	8	IFN-α or lymphoblastoid	45/271 ^a^32/271	24/74 ^a^19/74	unknown
Yu ML et al. (2006) [39]	Taiwan	Tx: 46.9, control: 43.6 (mean)	mean 5.18–5.15 years	R	46.2% type 1	8	IFN-α with or without RBV	16/1057 ^a^14/1057	12/562 ^a^10/562	LC 15.6% in Tx, 12.1% in control
Di Martino V et al. (2011) [41]	France	unknown	median 59 months	R	57.9% type 1	7	IFN with or without RBV, or PegIFN with RBV	9/184 ^a^5/184	20/194 ^a^4/184	55.5% F2 or greater
Yamasaki K et al. (2012) [49]	Japan	60.9 (mean)	median 11.5 years	P	59.9% type 1b	7	IFN-α or β or lymphoblastoid with or without RBV	25/152 ^a^6/152	90/199 ^a^32/199	unknown
Maruoka D et al. (2012) [43]	Japan	50.4–54 (mean)	mean 9.9 years	R	73.6% type 1	8	IFN-α/IFN-β with or without RBV	84/577 ^a^52/577	37/144 ^a^30/144	F3,4: 24.3% in Tx, F4: 43.1% in control
Cozen ML et al. (2013) [44]	US	50.98 (mean)	mean 10 years	R	68.7% type 1	8	IFN-α with or without RBV	31/159	47/199	F3,4: 19% (30.2% in Tx, 10.1% in control)
Aleman S et al. (2013) [45]	Sweden	51 (mean)	mean 5.3 years	R	50% type 1	8	PegIFN with RBV	59/303 ^a^39/303	18/48 ^a^16/48	LC
Kutala BK et al. (2015) [50]	France	50 (median)	median 5.5 years	R	55.7% type 1	8	IFN/PegIFN with or without RBV	30/325	19/102	F3,4: 100%
Cozen ML et al. (2016) [46]	US	51.4 (mean)	mean 8.5 years	P	71.6% type 1 or 4	8	IFN-α with RBV	112/692	488/1519	LC 15.8% in Tx, 5.3% in control

^a^: Liver-specific death, *CHC* chronic hepatitis C, *NOS* Newcastle-Ottawa scale, *Tx* treatment group, *R* retrospective cohort study, *P* prospective cohort study, *RCT* randomized controlled study, *IFN* interferon, *PegIFN* pegylated interferon, *RBV* ribavirin, *LC* liver cirrhosis, *SVR* sustained virologic response

**Table 5:** Clinical data of included studies for the efficacy of SVR on the development of HCC in patients with CHC

Study	Nationality	Age	Duration of follow up	Study format	Genotype	NOS	Treatment	HCC/Total SVR	HCC/No SVR	Histology
Nishiguchi S et al. (1995) [51]	Japan	Tx: 54.7, control: 57.3 (mean)	2–7 years	RCT	75.6% type 2		IFN-α	0/7	2/38	HAI 11.7 in Tx, 11.8 in control (mean)
Tanaka K et al. (1998) [52]	Japan	SVR: 47.7, no SVR: 51 (mean)	about 40 months	P	unknown	7	lymphoblastoid IFN	0/8	10/47	LC
Yoshida H et al. (1999) [30]	Japan	Tx: 49.5, control: 53.6 (mean)	median 4.3 years	R	70.3% type 1	7	IFN-α or IFN-β or combination	10/789	79/1611	F3,4: 33.1% in Tx, 33.8% in control
Testino G et al. (2002) [16]	Italy	Tx: 55.3, control: 56.8 (mean)	mean 95.4 months	R	55% type 1b, 45% type 2	8	IFN-α	3/11	12/40	Child A LC
Okanoue T et al. (2002) [53]	Japan	Tx: 50.4, control: 58.1 (mean)	Mean 5.6 years	R	unknown	7	IFN-α or lymphoblastoid	4/426	110/994	F3,4: 20.9% in SVR, 34.4% in control
Coverdale SA et al. (2004) [36]	Australia	Tx: 37, control: 38 (median)	median 9 years	P	39.6% type 1	7	IFN-α	1/50	25/334	Scheuer fibrosis score 2
Shiratori Y et al. (2005) [38]	Japan	Tx: 57, control: 61 (median)	median 6.8 years	P	71.9% type 1b	8	IFN-α or lymphoblastoid	11/64	73/207	unknown
Yu ML et al. (2006) [39]	Taiwan	Tx: 46.9, control: 43.6 (mean)	mean 5.18–5.15 years	R	46.2% type 1	8	IFN-α with or without RBV	12/715	39/342	LC 15.6% in Tx, 12.1% in control
Pradat P et al. (2007) [54]	Europe	45–47 (mean)	5–7 years	P	49.2% type 1	6	IFN/PegIFN with or without RBV	0/91	17/266	unknown
Braks RE et al. (2007) [55]	France	54.1 (mean)	mean 7.7 years	R	61.1% type 1	8	IFN-α with or without RBV, or PegIFN with RBV	1/37	24/76	Child A LC
Bruno S et al. (2007) [56]	Italy	54.7 (mean)	Mean 96.1 months	R	71.8% type 1	8	IFN-α	7/124	122/759	Child A LC
Hasegawa E et al. (2007) [57]	Japan	56 (median)	median 4.6 years	R	65% 2a	7	IFN-α,β/lymphoblastoid with or without RBV	3/48	16/57	LC
Veldt BJ et al. (2007) [58]	Europe and Canada	48 (median)	median 2.1 years	R	59% type 1	8	IFN/PegIFN with or without RBV	3/142	32/337	Ishak 4–6
Floreani A et al. (2008) [59]	Italy	44.5–55.7 (mean)	mean 23.4–25.2 months	R	41.3% type 1	7	PegIFN with RBV	0/40	5/38	unknown
Sinn DH et al. (2008) [40]	Korea	48.4–58.2 (mean)	median 55.2 months	R	48.6% type 2	7	IFN/PegIFN with or without RBV	4/296	10/194	F3,4: 49% in Tx, F4: 33% in control
Kurokawa M et al. (2009) [60]	Japan	55.8 (mean)	median 36.5 months	R	72.9% type 1	7	IFN-α with RBV	4/139	21/264	F3,4: 31.3%
Asahina Y et al. (2010) [61]	Japan	55.4 (mean)	mean 7.5 years	R	69.6% type 1b	8	IFN-α,β with or without RBV, or PegIFN with RBV	22/686	149/1356	F3,4: 25.2%
Kawamura Y et al. (2010) [62]	Japan	50 (median)	median 6.7 years	R	unknown	8	IFN-α,β with or without RBV	12/1081	61/977	F1,2: 93.1%
Cardoso AC et al. (2010) [63]	France	55 (mean)	median 3.5 years	R	60% type 1	7	IFN/PegIFN with or without RBV	6/103	40/204	F3,4: 100%
Morgan TR et al. (2010) [64]	US	48.6–49.6 (mean)	median 79–96 months	P	87.2% type 1	8	PegIFN with or without RBV	2/140	33/386	F3,4: 100%
Di Martino V et al. (2011) [41]	France	unknown	median 59 months	R	57.9% type 1	7	IFN with or without RBV, or PegIFN with RBV	1/59	8/125	55.5% F2 or greater
Velosa J et al. (2011) [65]	Portugal	51.7 (mean)	mean 6.4 years	R	61% type 1	7	IFN/PegIFN with or without RBV	1/39	20/91	compensated LC
Iacobellis A et al. (2011) [66]	Italy	59–62 (mean)	mean 51 months	P	57.3% type 1	7	PegIFN with RBV	5/24	11/51	decompensated LC
Hung CH et al. (2011) [67]	Taiwan	53 (median)	median 4.3 years	R	49% type 1	7	IFN/PegIFN with or without RBV	33/1027	54/443	unknown
Takahashi H et al. (2011) [68]	Japan	55.4 (mean)	Mean 52 months	R	74.9% type 1b	7	IFN-α,β/PegIFN with RBV	1/89	12/114	F3,4: 23.2%
Backus LI et al. (2011) [69]	US	51–53 (mean)	median 3.8 years	R	72.1% type 1	6	PegIFN with RBV	223/7434	283/1440	13% LC
Tateyama M et al. (2011) [42]	Japan	57 (median)	mean 8.2 years	R	72.1% type 1b	8	IFN/PegIFN with or without RBV	3/139	44/234	F3,4: 34.1%
Osaki Y et al. (2012) [70]	Japan	59 (median)	median 4.1 years	R	59.9% type 1	7	IFN/PegIFN with RBV	1/185	22/197	unknown
van der Meer AJ et al. (2012) [71]	Europe and Canada	48 (mean)	median 8.4 years	R	68% type 1	8	IFN/PegIFN with or without RBV	7/125	76/405	Ishak 4–6
Maruoka D et al. (2012) [43]	Japan	50.4–54 (mean)	mean 9.9 years	R	73.6% type 1	8	IFN-α/IFN-β with or without RBV	5/221	80/356	F3,4: 24.3% in Tx, F4: 43.1% in control
Cozen ML et al. (2013) [44]	US	50.98 (mean)	mean 10 years	R	68.7% type 1	8	IFN-α with or without RBV	2/69	9/90	F3,4: 19% (30.2% in Tx, 10.1% in control)
Alfaleh FZ et al. (2013) [72]	Saudi Arabia, Egypt	48 (mean)	mean 63.8 months	P	30.6% type 4	8	PegIFN with or without RBV	0/62	4/95	F3,4: 24.6% (27.1% in SVR, 31.1% in no SVR)
Aleman S et al. (2013) [45]	Sweden	51 (mean)	mean 5.3 years	R	50% type 1	8	PegIFN with RBV	6/110	26/193	LC
Di Marco V et al. (2016) [73]	Italy	58 (mean)	median 7.6 years	P	83.4% type 1	8	PegIFN with RBV	7/108	92/336	compensated LC
Ikezaki H et al. (2016) [74]	Japan	60–64 (median)	median 2.8 years	R	52.7% in type 1	7	IFN- β with RBV	2/68	7/44	F3,4: 30.9% in SVR, 72.7% in no SVR

*SVR* sustained virologic response, *HCC* hepatocellular carcinoma, *CHC* chronic hepatitis C, *NOS* Newcastle-Ottawa scale, *Tx* treatment group, *R* retrospective cohort study, *P* prospective cohort study, *RCT* randomized controlled study, *IFN* interferon, *PegIFN* pegylated interferon, *RBV* ribavirin, *LC* liver cirrhosis

**Table 6:** Clinical data of included studies for the efficacy of SVR on all-cause and liver-specific mortality in patients with CHC

Study	Nationality	Age	Duration of follow up	Study format	Genotype	NOS	Treatment	Death/Total SVR	Death/No SVR	Histology
Yosida H et al. (2002) [47]	Japan	Tx: 49.5, control: 54.6 (mean)	mean 5.4 years	R	unknown	8	IFN-α or IFN-β	^a^7/817	^a^49/1613	F3,4: 32.2% in Tx, 31.6% in control, 26.3% in SVR, 35.2% in no SVR
Okanoue T et al. (2002) [53]	Japan	Tx: 50.4, control: 58.1 (mean)	Mean 5.6 years	R	unknown	7	IFN-α or lymphoblastoid	2/426 ^a^0/426	47/994 ^a^34/994	F3,4: 20.9% in SVR, 34.4% in control
Imazeki F et al. (2003) [48]	Japan	Tx: 49.2, control: 53.1 (mean)	mean 8.2 years	R	73.9% type 1	8	IFN-α or IFN-β	4/116 ^a^1/116	29/239 ^a^18/239	F3,4: 26.7% in Tx, 29.8% in control
Coverdale SA et al. (2004) [36]	Australia	Tx: 37, control: 38 (median)	median 9 years	P	39.6% type 1	7	IFN-α	^a^1/50	^a^35/334	Scheuer fibrosis score 2
Kasahara A et al. (2004) [17]	Japan	Tx: 53, control: 54 (median)	mean 6 years	R	unknown	8	IFN	7/738 ^a^1/738	94/1930 ^a^68/1930	F3,4: 38.7% in Tx, 48% in control, 28.6% in SVR, 43% in no SVR
Shiratori Y et al. (2005) [38]	Japan	Tx: 57, control: 61 (median)	median 6.8 years	P	71.9% type 1b	8	IFN-α or lymphoblastoid	1/64 ^a^0/64	44/207 ^a^32/207	unknown
Yu ML et al. (2006) [39]	Taiwan	Tx: 46.9, control: 43.6 (mean)	mean 5.18–5.15 years	R	46.2% type 1	8	IFN-α with or without RBV	4/715 ^a^3/715	12/342 ^a^11/342	LC 15.6% in Tx, 12.1% in control
Arase Y et al. (2007) [75]	Japan	SVR: 63, no SVR: 64 (mean)	mean 7.4 years	R	60.4% type 1b	8	IFN-α/β with or without RBV	9/140 ^a^2/140	44/360 ^a^32/360	F3,4: 14.5 in SVR, 27.5 in no SVR
Bruno S et al. (2007) [56]	Italy	54.7 (mean)	Mean 96.1 months	R	71.8% type 1	8	IFN-α	6/124 ^a^2/120	114/759 ^a^83/728	Child A LC
Veldt BJ et al. (2007) [58]	Europe and Canada	48 (median)	median 2.1 years	R	59% type 1	8	IFN/PegIFN with or without RBV	2/142 ^a^1/142	24/337 ^a^19/337	Ishak 4–6
Cardoso AC et al. (2010) [63]	France	55 (mean)	median 3.5 years	R	60% type 1	7	IFN/PegIFN with or without RBV	^a^3/103	^a^18/204	F3,4: 100%
Morgan TR et al. (2010) [64]	US	48.6–49.6 (mean)	median 79–96 months	P	87.2% type 1	8	PegIFN with or without RBV	^a^1/140	^a^23/386	F3,4: 100%
Innes HA et al. (2011) [76]	UK	41.8 (mean)	mean 5.3 years	R	35.6% type 1	8	IFN/PegIFN with or without RBV	13/560 ^a^5/560	75/655 ^a^50/655	85.8% no LC
Di Martino V et al. (2011) [41]	France	unknown	median 59 months	R	57.9% type 1	7	IFN with or without RBV, or PegIFN with RBV	0/59 ^a^0/59	9/125 ^a^5/125	55.5% F2 or greater
Velosa J et al. (2011) [65]	Portugal	51.7 (mean)	mean 6.4 years	R	61% type 1	7	IFN/PegIFN with or without RBV	^a^0/39	^a^15/91	compensated LC
Iacobellis A et al. (2011) [66]	Italy	59–62 (mean)	mean 51 months	P	57.3% type 1	7	PegIFN with RBV	^a^2/24	^a^23/51	decompensated LC
Backus LI et al. (2011) [69]	US	51–53 (mean)	median 3.8 years	R	72.1% type 1	6	PegIFN with RBV	525/7434	1440/9430	13% LC
Yamasaki K et al. (2012) [49]	Japan	60.9 (mean)	median 11.5 years	P	59.9% type 1b	7	IFN-α or β or lymphoblastoid with or without RBV	9/72 ^a^1/72	16/80 ^a^5/80	unknown
van der Meer AJ et al. (2012) [71]	Europe and Canada	48 (mean)	median 8.4 years	R	68% type 1	8	IFN/PegIFN with or without RBV	13/125 ^a^3/125	100/405 ^a^103/405	Ishak 4–6
Maruoka D et al. (2012) [43]	Japan	50.4–54 (mean)	mean 9.9 years	R	73.6% type 1	8	IFN-α/IFN-β with or without RBV	10/221 ^a^2/221	74/356 ^a^50/356	F3,4: 24.3% in Tx, F4: 43.1% in control
Cozen ML et al. (2013) [44]	US	50.98 (mean)	mean 10 years	R	68.7% type 1	8	IFN-α with or without RBV	6/69	25/90	F3,4: 19% (30.2% in Tx, 10.1% in control)
Alfaleh FZ et al. (2013) [72]	Saudi Arabia, Egypt	48 (mean)	mean 63.8 months	P	30.6% type 4	8	PegIFN with or without RBV	0/62 ^a^0/62	4/95 ^a^8/95	F3,4: 24.6% (27.1% in SVR, 31.1% in no SVR)
Aleman S et al. (2013) [45]	Sweden	51 (mean)	mean 5.3 years	R	50% type 1	8	PegIFN with RBV	11/110 ^a^4/110	48/193 ^a^35/193	LC
Singal AG et al. (2013) [77]	US	48 (median)	median 36–72 months	R	68.6% type 1	7	PegIFN with RBV	2/83	41/159	17.3% LC
Dieperink E et al. (2014) [78]	US	51.4 (mean)	median 7.5 years	R	70% type 1	8	IFN/PegIFN with or without RBV	19/222 ^a^6/222	81/314 ^a^56/314	F3,4: 54.5% (41.3% in SVR, 64.7% in no SVR)
Kutala BK et al. (2015) [50]	France	50 (median)	median 5.5 years	R	55.7% type 1	8	IFN/PegIFN with or without RBV	3/104	27/221	F3,4: 100%
Di Marco V et al. (2016) [73]	Italy	58 (mean)	median 7.6 years	P	83.4% type 1	8	PegIFN with RBV	^a^8/108	^a^98/336	compensated LC

^a^: Liver-specific death, *SVR* sustained virologic response, *CHC* chronic hepatitis C, *NOS* Newcastle-Ottawa scale, *Tx* treatment group, *R* retrospective cohort study, *P* prospective cohort study, *RCT* randomized controlled study, *IFN* interferon, *PegIFN* pegylated interferon, *RBV* ribavirin, *LC* liver cirrhosis

### Methodological quality

The methodological quality of cohort study is described in the Table 3, 4, 5 and 6. This feature was evaluated as modifiers in each analysis. The methodological quality of RCT is described in Additional file 1: Appendix 1. Given the similar methodological quality among RCTs, sensitivity analysis or subgroup analyses based on the methodological quality in RCTs were not performed.

### Efficacy of antiviral treatment on the development of HCC in chronic hepatitis C patients

**Fig. 2:**
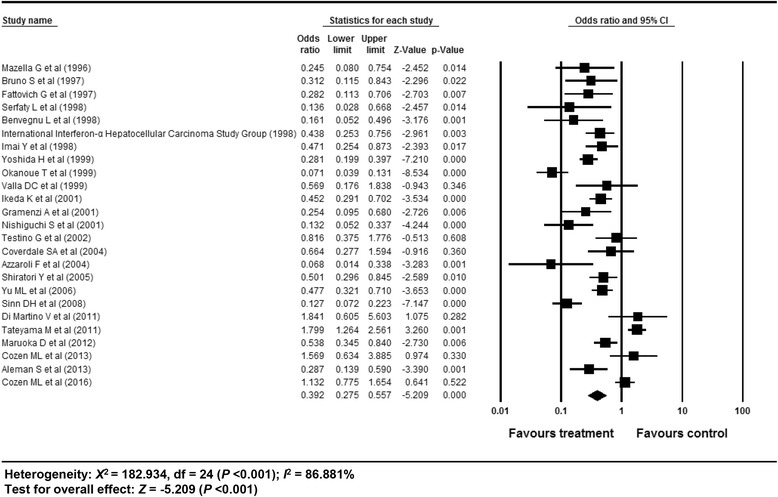
Efficacy of antiviral treatment on the development of HCC in patients with CHC. The size of each square is proportional to the study’s weight. Diamond is the summary estimate from the pooled studies (random effect model). HCC, hepatocellular carcinoma; CHC, chronic hepatitis C

The overall efficacy of antiviral treatment on the development of HCC exhibited an OR of 0.392 (95% CI: 0.275–0.557, *p* <0.001) in a random effect model analysis (Fig. 2). The funnel plot showed asymmetry on the right lower quadrant area (Additional file 1: Appendix Figure S2). However, the Egger’s test revealed an intercept of −2.131 (95% CI: −4.81–0.54, t-value: 1.64, df: 23, *p* = 0.11 (2-tailed)). The rank correlation test also showed a Kendall’s tau of −0.19 with a continuity correction (*p* = 0.17). The trim and fill method indicated that no study was trimmed. Overall, there was no evidence of publication bias.

A cumulative meta-analysis of enrolled studies based on publication year showed no specific time trend (Additional file 1: Appendix 3). A cumulative meta-analysis based on effect size showed no small study bias (Additional file 1: Appendix 4). One study removed meta-analysis revealed a stable feature (Additional file 1: Appendix 5). Overall, the sensitivity meta-analyses revealed robust results.

Methodological quality of Newcastle-Ottawa scale potentially explained heterogeneity in meta-ANOVA tests (*p* = 0.027) (Additional file 1: Appendix 6). A meta-regression revealed a Newcastle-Ottawa scale score of 8 for the reason of heterogeneity (*p* = 0.027) (Additional file 2: Table S1). After excluding 10 studies (Newcastle-Ottawa scale 8), no covariates explained heterogeneity in meta-regression tests. Therefore, methodological quality was the reason of heterogeneity in this analysis.

### Efficacy of antiviral treatment on All-cause mortality in patients with chronic hepatitis C

**Fig. 3:**
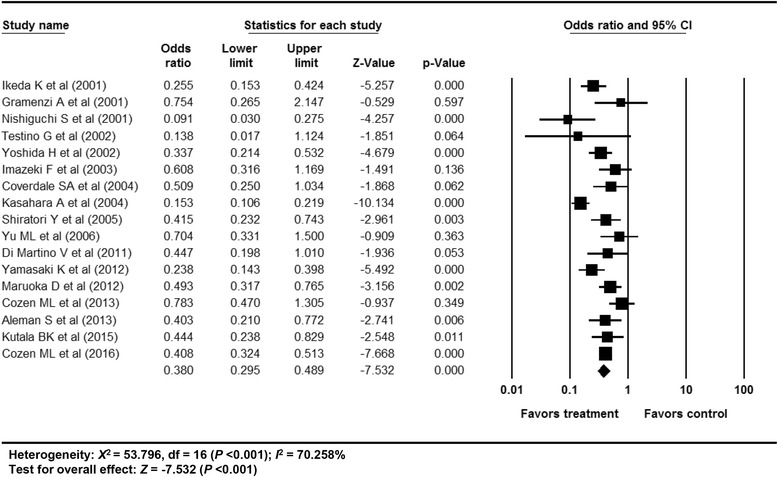
Efficacy of antiviral treatment on all-cause mortality in patients with CHC. The size of each square is proportional to the study’s weight. Diamond is the summary estimate from the pooled studies (random effect model). CHC, chronic hepatitis C

The overall efficacy of antiviral treatment on all-cause mortality revealed an OR of 0.380 (95% CI: 0.295–0.489, *p* <0.001) in a random effect model analysis (Fig. 3). The funnel plot showed asymmetry on the right lower quadrant area (Additional file 1: Appendix 7). However, the Egger’s test revealed an intercept of 0.266 (95% CI: −2.010–2.542, t-value: 0.25, df: 15, *p* = 0.81 (2-tailed)). The rank correlation test also showed a Kendall’s tau of 0.04 with a continuity correction (*p* = 0.84). The trim and fill method indicated that 1 study was trimmed. After excluding the study by Testino et al. [16] located on the left lower quadrant in funnel plot, the OR was 0.385 (95% CI: 0.298–0.496, *p* <0.001). Overall, the impact of bias was minimal.

A cumulative meta-analysis of enrolled studies based on publication year showed no specific time trend (Additional file 1: Appendix 8). A cumulative meta-analysis based on effect size showed no small study bias (Additional file 1: Appendix 9). One study removed meta-analysis revealed a stable feature (Additional file 1: Appendix 10). Overall, the sensitivity meta-analyses revealed robust results.

Meta-ANOVA or meta-regression showed no specific modifier for the reason of heterogeneity (Additional file 1: Appendix 11) (Additional file 2: Table S2). Overall, no covariates were found to be explaining heterogeneity in this meta-analysis.

### Efficacy of antiviral treatment on liver-specific mortality in chronic hepatitis C patients

The overall efficacy of antiviral treatment on liver-specific mortality exhibited an OR of 0.363 (95% CI: 0.260–0.508, *p* <0.001) in a random effect model analysis (Additional file 1: Appendix 12). The funnel plot showed symmetry (Additional file 1: Appendix 13). However, the Egger’s test revealed that intercept was 3.06 (95% CI: 0.295–5.831, t-value: 2.43, df: 11, *p* = 0.03 (2-tailed)). The rank correlation test showed a Kendall’s tau of 0.28 with a continuity correction (*p* = 0.20). The trim and fill method indicated that no study was trimmed. After excluding an outlier (study by Kasahara A et al. [17]) located on the left upper quadrant area in funnel plot, the OR was 0.398 (95% CI: 0.314–0.504, *p* <0.001). Overall, the impact of bias was minimal.

A cumulative meta-analysis of enrolled studies based on publication year showed no specific time trend (Additional file 1: Appendix 14). A cumulative meta-analysis based on effect size showed no small study bias (Additional file 1: Appendix 15). One study removed meta-analysis revealed a stable feature (Additional file 1: Appendix 16). Overall, the sensitivity meta-analyses showed robust results.

A meta-ANOVA indicated that follow-up duration (*p* = 0.036) and methodological quality (*p* = 0.029) were suspicious for the reason of heterogeneity (Additional file 1: Appendix 17). A meta-regression indicated that follow-up duration (*p* = 0.036) and Newcastle-Ottawa scale score of 8 (*p* = 0.029) explained the heterogeneity (Additional file 2: Table S3). After excluding 2 studies (short-term follow-up duration), no covariates explained heterogeneity in meta-regression tests. After excluding 7 studies (Newcastle-Ottawa scale 8), no covariates explained heterogeneity in meta-regression tests. Therefore, follow-up duration and methodological quality were the reasons of heterogeneity in this analysis.

### Efficacy of SVR on the development of HCC in patients with chronic hepatitis C

**Fig. 4:**
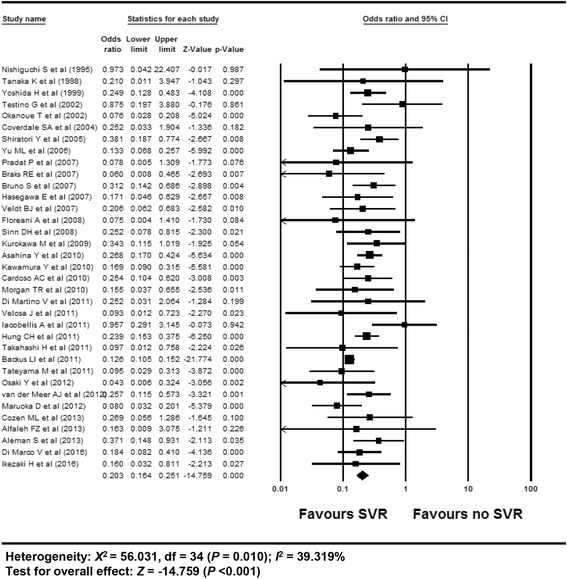
Efficacy of SVR on the development of HCC in patients with CHC. The size of each square is proportional to the study’s weight. Diamond is the summary estimate from the pooled studies (random effect model). SVR, sustained virologic response; HCC, hepatocellular carcinoma; CHC, chronic hepatitis C

The overall efficacy of SVR on the development of HCC exhibited an OR of 0.203 (95% CI: 0.164–0.251, *p* <0.001) in a random effect model analysis (Fig. 4). The funnel plot showed symmetry (Additional file 1: Appendix 18). The Egger’s test showed that intercept was 0.56 (95% CI: −0.099–1.217, t-value: 1.73, df: 33, *p* = 0.09 (2-tailed)). The rank correlation test showed a Kendall’s tau of −0.17 with a continuity correction (*p* = 0.16). The trim and fill method indicated that no study was trimmed. Overall, there was no evidence of publication bias.

A cumulative meta-analysis of enrolled studies based on publication year showed no specific time trend (Additional file 1: Appendix 19). A cumulative meta-analysis based on effect size showed no small study bias (Additional file 1: Appendix 20). One study removed meta-analysis showed a stable feature (Additional file 1: Appendix 21). Overall, the sensitivity meta-analyses revealed robust results.

Meta-ANOVA or meta-regression identified no specific modifier for the reason of heterogeneity (Additional file 1: Appendix 22) (Additional file 2: Table S4). Overall, no covariates explained heterogeneity.

### Efficacy of SVR on all-cause mortality in patients with chronic hepatitis C

**Fig. 5:**
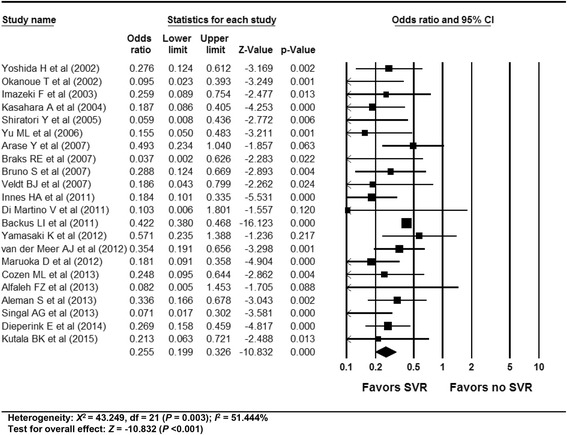
Efficacy of SVR on all-cause mortality in patients with CHC. The size of each square is proportional to the study’s weight. Diamond is the summary estimate from the pooled studies (random effect model). SVR, sustained virologic response; CHC, chronic hepatitis C

The overall efficacy of SVR on all-cause mortality revealed an OR of 0.255 (95% CI: 0.199–0.326, *p* < 0.001) in a random effect model analysis (Fig. 5). The funnel plot showed asymmetry on the right lower quadrant area (Additional file 1: Appendix 23). The Egger’s test showed that the intercept was −1.44 (95% CI: −1.921– −0.949, t-value: 6.16, df: 20, *p* <0.001 (2-tailed)). The rank correlation test showed a Kendall’s tau of −0.23 with a continuity correction (*p* = 0.14). The trim and fill method indicated 11 studies were trimmed. Overall, there was evidence of publication bias.

A cumulative meta-analysis of enrolled studies based on publication year showed no specific time trend (Additional file 1: Appendix 24). A cumulative meta-analysis based on effect size showed no small study bias (Additional file 1: Appendix 25). One study removed meta-analysis revealed a stable feature (Additional file 1: Appendix 26). Overall, the sensitivity meta-analyses showed robust results.

Meta-ANOVA indicated that methodological quality potentially explained heterogeneity (*p* = 0.030) (Additional file 1: Appendix 27). Meta-regression revealed a Newcastle-Ottawa scale score of 8 for the reason of heterogeneity (Additional file 2: Table S5). After excluding 16 studies (Newcastle-Ottawa scale 8), no covariates explained heterogeneity in meta-regression tests. Therefore, methodological quality was the reasons of heterogeneity in this analysis.

### Efficacy of SVR on liver-specific mortality in chronic hepatitis C patients

The overall efficacy of SVR on liver-specific mortality exhibited an OR of 0.126 (95% CI: 0.094–0.169, *p* < 0.001) in a random effect model analysis (Additional file 1: Appendix 28). The funnel plot showed asymmetry on the right lower quadrant area (Additional file 1: Appendix 29). The Egger’s test indicated that intercept was −0.77 (95% CI: −1.473 – −0.057, t-value: 2.25, df: 21, *p* = 0.036 (2-tailed)). The rank correlation test revealed a Kendall’s tau of −0.19 with a continuity correction (*p* = 0.20). The trim and fill method showed 6 studies were trimmed. Overall, there was evidence of publication bias.

A cumulative meta-analysis of enrolled studies based on publication year showed no specific time trend (Additional file 1: Appendix 30). A cumulative meta-analysis based on effect size showed no small study bias (Additional file 1: Appendix 31). One study removed meta-analysis revealed a stable feature (Additional file 1: Appendix 32). Overall, the sensitivity meta-analyses showed robust results.

Meta-ANOVA or meta-regression revealed no specific modifier for the reason of heterogeneity (Additional file 1: Appendix 33) (Additional file 2: Table S6). Overall, no covariates explained heterogeneity.

The results of meta-regression analyses for each topic are summarized in Table 7.

**Table 7:** Results of meta-regression analyses

Modifier	Coefficient	Standard error	*P* value
NOS (topic 1)	NOS 8: 1.203 NOS 7: 0.501	0.565 0.561	0.033 0.372 Q: 7.24, df: 2, *P* = 0.027
Follow-up duration (topic 3)	1.140	0.542	0.036
NOS (topic 3)	NOS 8: −0.659	0.302	0.029
NOS (topic 5)	NOS 8: −0.540 NOS 7: −0.544	0.209 0.322	0.010 0.091 Q: 7.03, df: 2, *P* = 0.030

*NOS* Newcastle-Ottawa scale

## Discussion

This meta-analyses confirmed the long-term efficacy of antiviral treatment in terms of prevention of HCC and reduction in all-cause and liver-specific mortality in patients with chronic HCV infection. This long-term efficacy was also intensified when SVR was achieved. Clinical outcomes regarding the efficacy of antiviral therapy in CHC patients have been continuously investigated by previous studies with a small number of patients or short-term follow-up duration. The reasons for performing this meta-analysis were a persistent risk of HCC even after attainment of SVR and a lack of sufficient data regarding long-term efficacy [18]. Persistent low-level of viremia and dysplastic hepatocyte regeneration are representative grounds for persistent risk of HCC after antiviral treatment [19, 20]. Interestingly, a recent meta-analysis revealed that IFN nonresponders exhibited a decreased risk of HCC recurrence after curative treatment of HCC, compared with no treatment patients, thus indicating that reduced necroinflammation and an inhibition of hepatic fibrosis progression prevent the development of HCC [21]. This results is consistent with that of our study and emphasized the importance of screening strategy of chronic hepatitis C.

Early antiviral treatment before progression to advanced fibrosis or cirrhosis is associated with an increasing probability of achieving SVR [22]. However, an indolent course of chronic hepatitis C makes it difficult for early diagnosis and treatment. Authors have revealed that favorable antiviral efficacy persists in all patients with chronic hepatitis C, regardless of histology. This result was also confirmed by a previous study indicating favorable antiviral efficacy even in patients with LC [18]. Considering the advanced fibrosis or cirrhosis is the sequelae of long-standing inflammation of liver, our study confirmed antiviral treatment is still valid in the late course of chronic hepatitis C. Although histology was not a significant modifier in our meta-analysis, all of the included studies have substantially heterogeneous populations regarding the degree of fibrosis or cirrhosis of the liver. This finding was commonly detected in a previous meta-analysis [18]. However, considering the expanding treatment indication, including decompensated LC by the advent of direct-acting antiviral agents, histology is not expected to affect the long-term efficacy of antiviral treatment in the near future.

Despite the favorable efficacy of antiviral treatment, 2 modifiers associated with heterogeneity were identified in the meta-ANOVA and meta-regression analyses. Studies with Newcastle-Ottawa scale of 8 were modifier in the analysis of association between antiviral treatment and the development of HCC (Additional file 2: Table S1), in the analysis of association between antiviral treatment and the liver-specific mortality (Additional file 2: Table S3), and in the analysis of association between SVR and all-cause mortality (Additional file 2: Table S5). Studies with a short-term follow-up duration were also modifier in the analysis of association between antiviral treatment and liver-specific mortality (Additional file 2: Table S3). Although these modifiers were confirmed as not significantly affecting the results of main analyses, this finding indicated the need for more number of high-quality and long-term follow-up studies on this topic.

Publication bias was detected in 2 topics (topic 5 and 6). Sensitivity analyses including cumulative and one study removed meta-analyses were rigorously performed to find the small study effect associated with publication bias, and these analyses showed no small study effect. Overall, the impact of publication bias was minimal.

This meta-analysis included the largest number of articles identified by a comprehensive literature search, and potential confounding modifiers were searched within each study whenever possible. Sensitivity analyses and meta-regression tests were performed to demonstrate robustness or identify the reason of heterogeneity. Despite the strengths, several limitations were detected during the systematic review. First, pretreatment predictive factors associated with the treatment response were not controlled or evaluated in these analyses, including pretreatment viral load, genotype, IL-28β polymorphism, and HBV or HIV coinfection. Direct-acting antiviral agents are expected to overcome these factors. Therefore, results of studies including these agents are expected in the near future. Second, the baseline characteristics of each enrolled study were not comparable between the treatment vs. no treatment groups, or the SVR vs. no SVR groups in some studies. This phenomenon was reflected in the evaluation of methodological quality and was confirmed to be a significant modifier associated with heterogeneity. Notably, difference by race or country including life style (obesity, consumption of alcohol or aflatoxin-contaminated foods, and chemical carcinogens exposure) was not appropriately investigated in our study. Considering the HCC is a heterogenous malignancy resulting from diverse causes of liver injury, different mechanisms or molecular pathways on the basis of country could be a cause of different treatment response. However, due to the heterogenous baseline characteristics including genotype and lacking of enough data about risk factors of HCC, the subgroup analyses by country could not present meaningful data. The limitations described above could be a cause of potential heterogeneity and bias. Therefore, studies controlling for various risk factors are needed to confirm these findings.

## Conclusion

In conclusion, antiviral treatment for chronic hepatitis C showed improved outcome in the development of HCC and mortality, especially when SVR is achieved, although studies controlling for various risk factors of HCC and mortality are still lacking.

## Additional files

Additional file 1: Contains 33 figures including assessment of methodological quality, funnel plots for publication bias, sensitivity analyses, and Meta-ANOVA. (DOC 24248 kb)

Additional file 2: Contains 6 tables including detailed meta-regression data of 6 study topics of this study. (DOC 79 kb)

## Abbreviations

CHC: Chronic hepatitis C
CI: Confidence interval
HCC: Hepatocellular carcinoma
HCV: Hepatitis C virus
IFN: Interferon
OR: Odds ratio
PegIFN: Pegylated interferon
RBV: Ribavirin
RCT: Randomized controlled studies
SVR: Sustained virologic response
