# Supplementary material for: Impact of antiviral therapy on hepatocellular carcinoma and mortality in patients with chronic hepatitis C: systematic review and meta-analysis
Source: BMC Gastroenterol. 2017 Apr 4;17:46. doi: 10.1186/s12876-017-0606-9 (PMC5379714; doi:10.1186/s12876-017-0606-9)
Supplement: Supplementary file 1 — Contains 33 figures including assessment of methodological quality, funnel plots for publication bias, sensitivity analyses, and Meta-ANOVA. (DOC 24248 kb) [file 12876_2017_606_MOESM1_ESM.doc]

**Additional file 1**

**Appendix 1.** RoB table for the assessment of methodological quality for randomized studies.

**
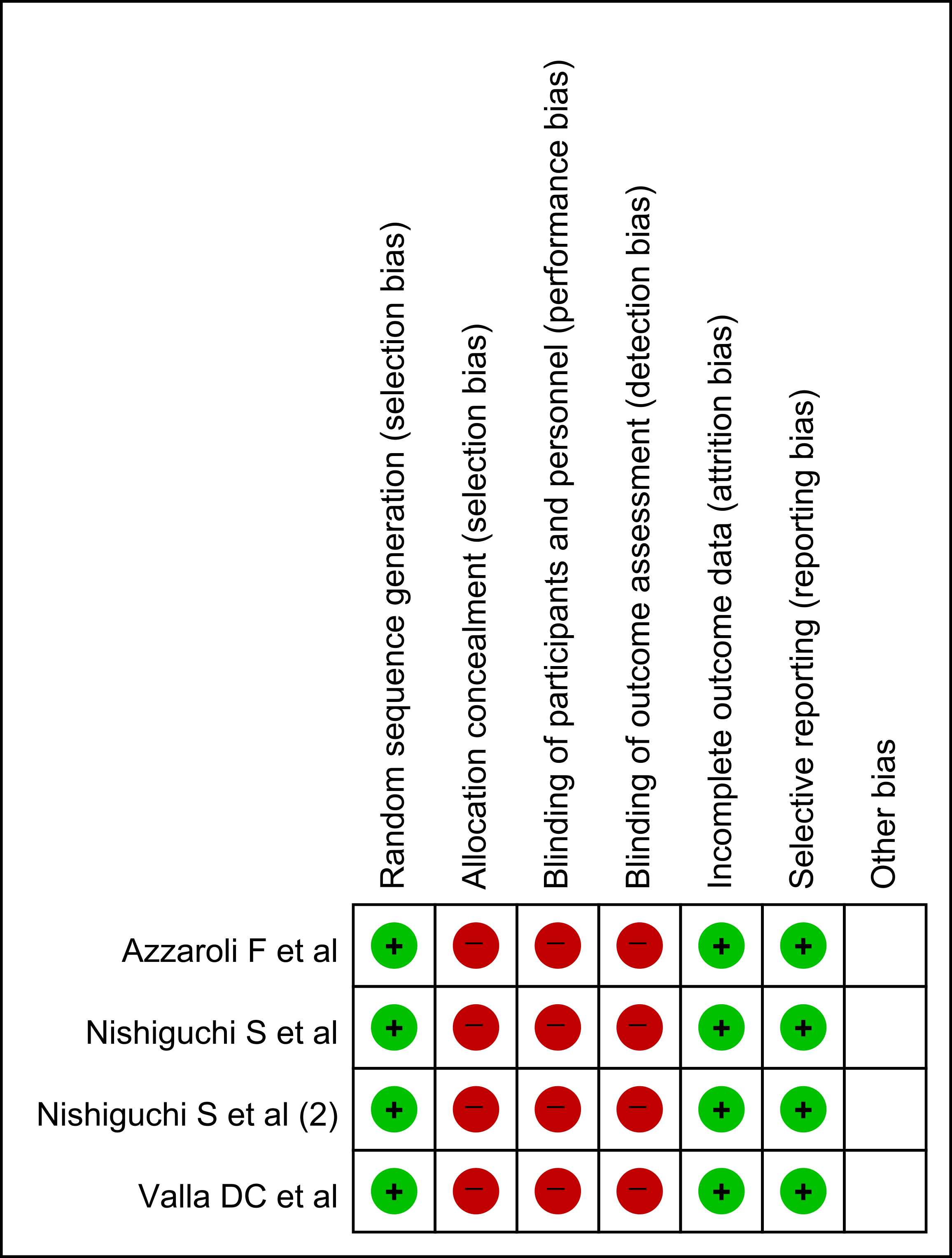
**

RoB, risk of bias. (+) denotes low risk of bias, blank denotes unclear risk of bias.

**Appendix 2.** Funnel plot of studies for efficacy of antiviral treatment on the development of HCC.

**
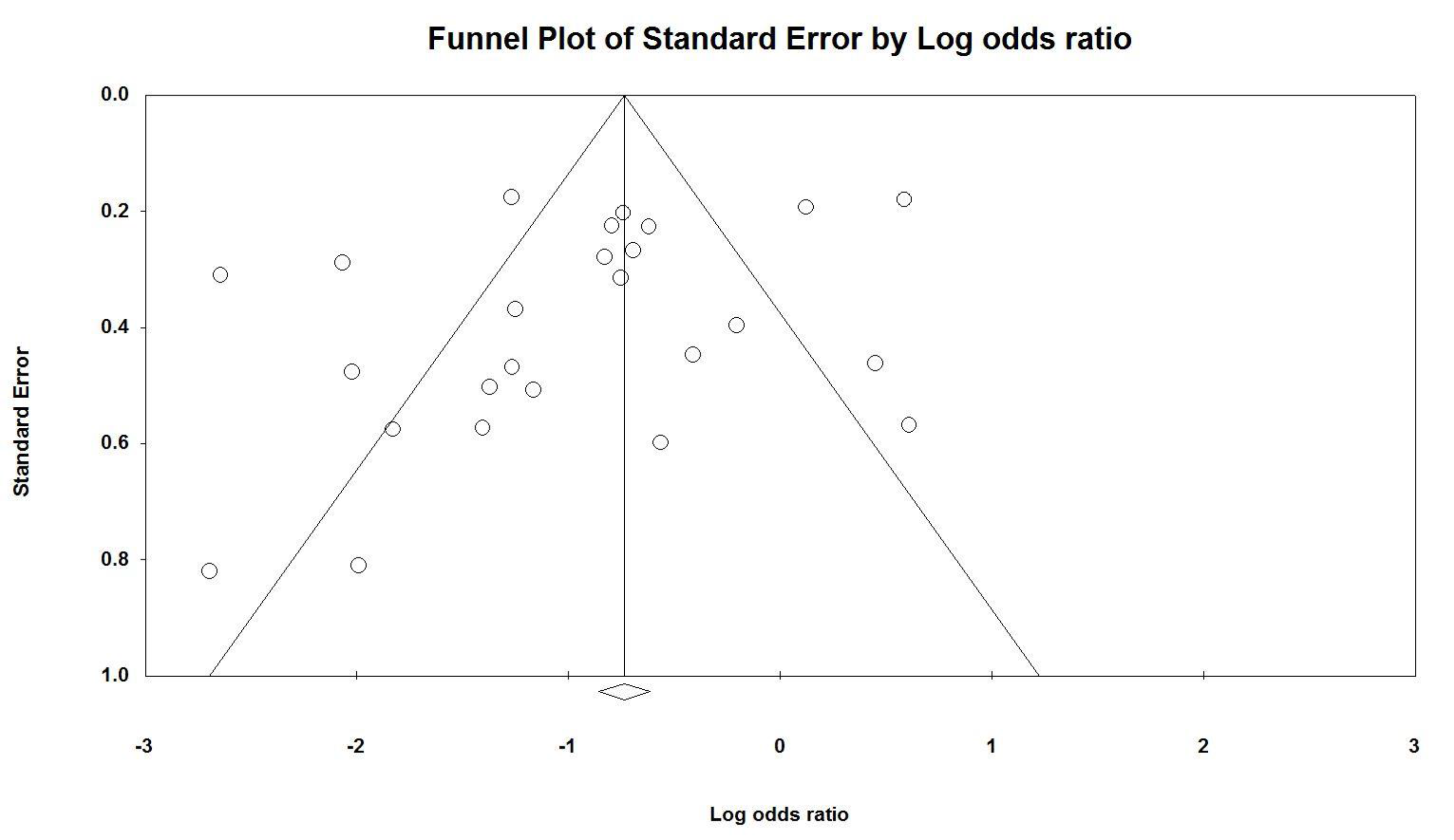
**

The line in center is the natural logarithm of pooled OR, and 2 oblique lines are pseudo 95% confidence limits. HCC, hepatocellular carcinoma; OR, odds ratio.

**Appendix 3.** Cumulative meta-analysis of enrolled studies for the efficacy of antiviral treatment on the development of HCC (based on publication year).

**
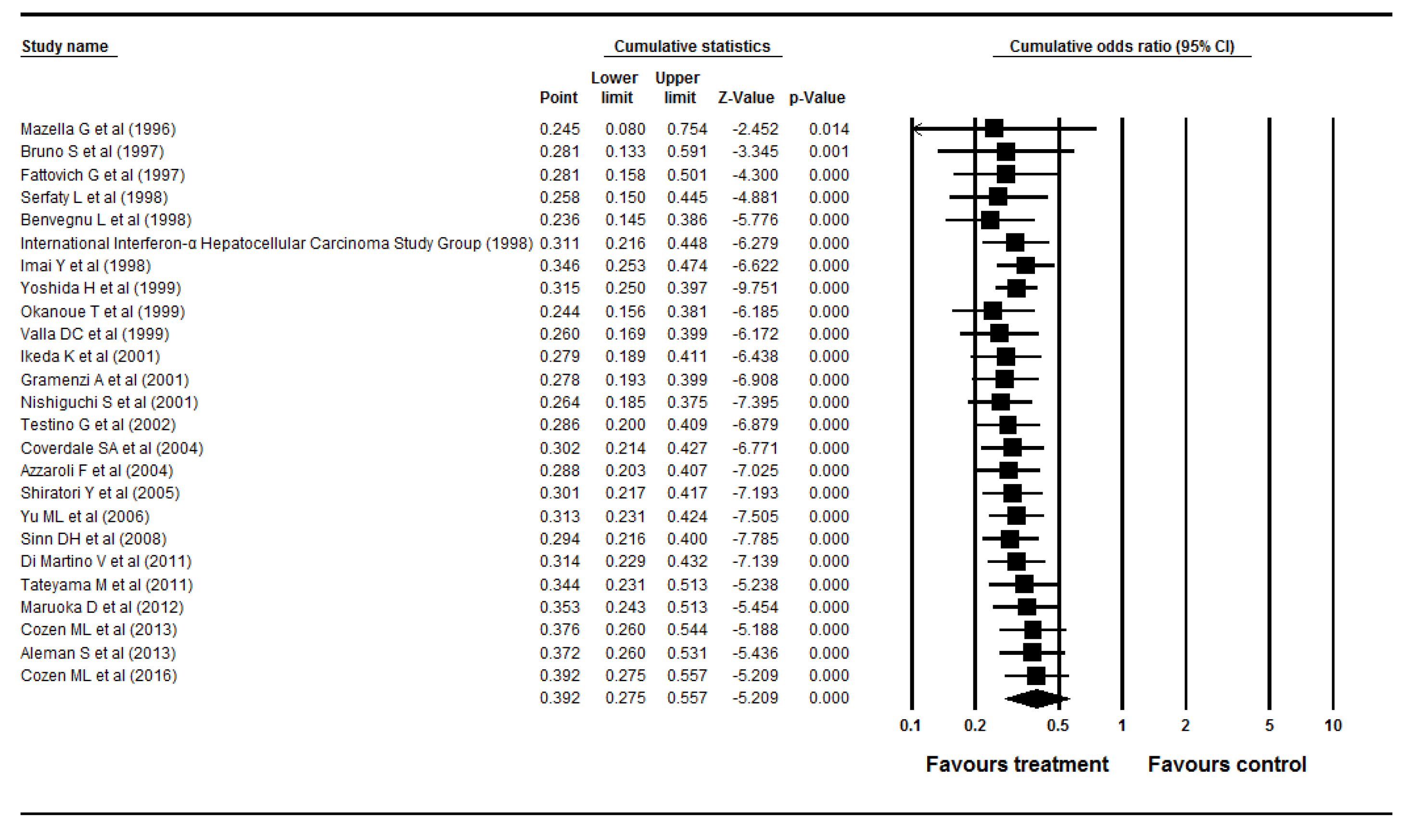
**

Diamond is the summary estimate from the pooled studies with 95% CI (Random effect model). HCC, hepatocellular carcinoma; CI, confidence interval.

**Appendix 4.** Cumulative meta-analysis of enrolled studies for the efficacy of antiviral treatment on the development of HCC (based on effect size).

**
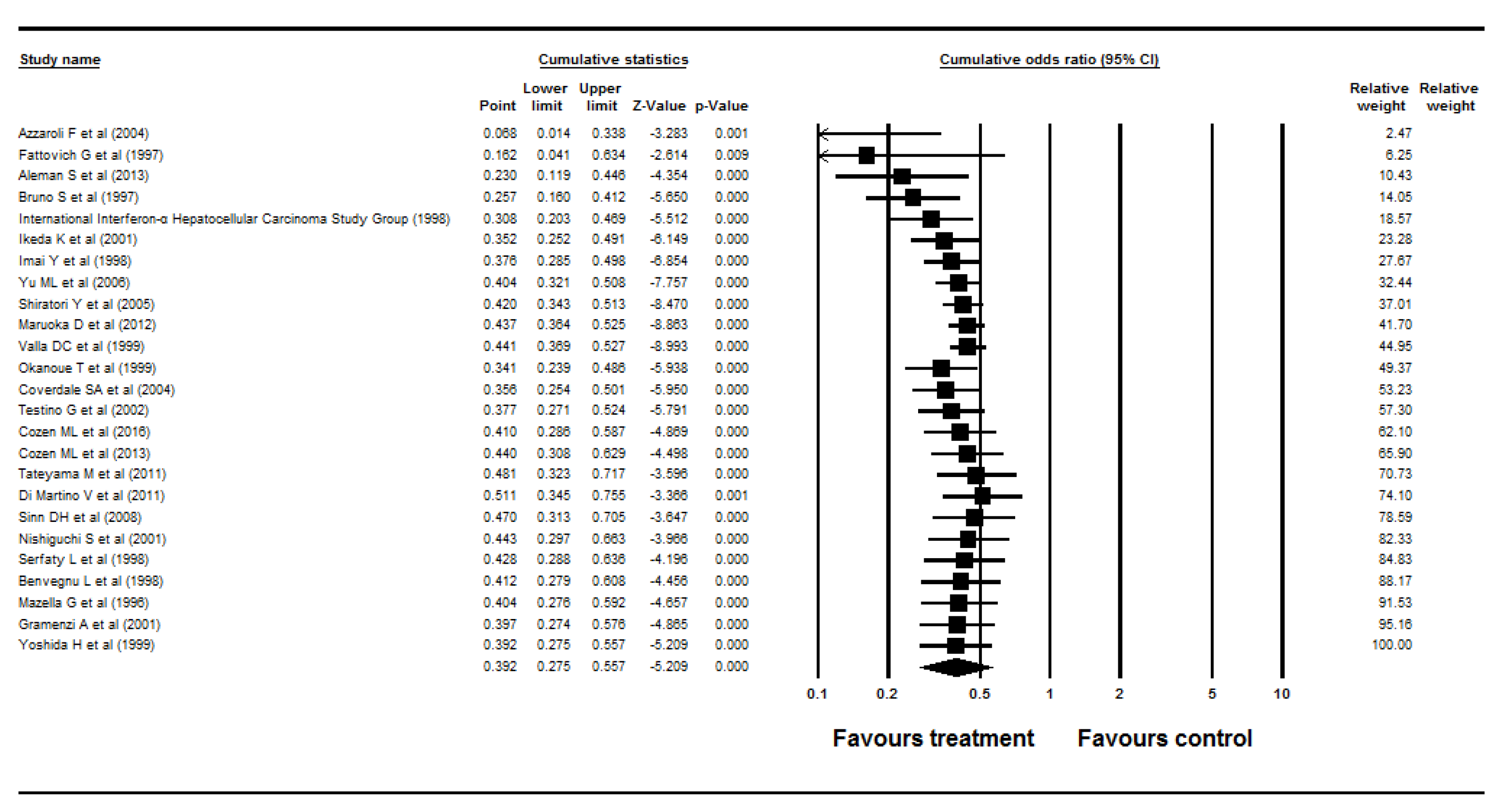
**

Diamond is the summary estimate from the pooled studies with 95% CI (Random effect model). HCC, hepatocellular carcinoma; CI, confidence interval.

**Appendix 5.** One study removed meta-analysis of enrolled studies for the efficacy of antiviral treatment on the development of HCC. **
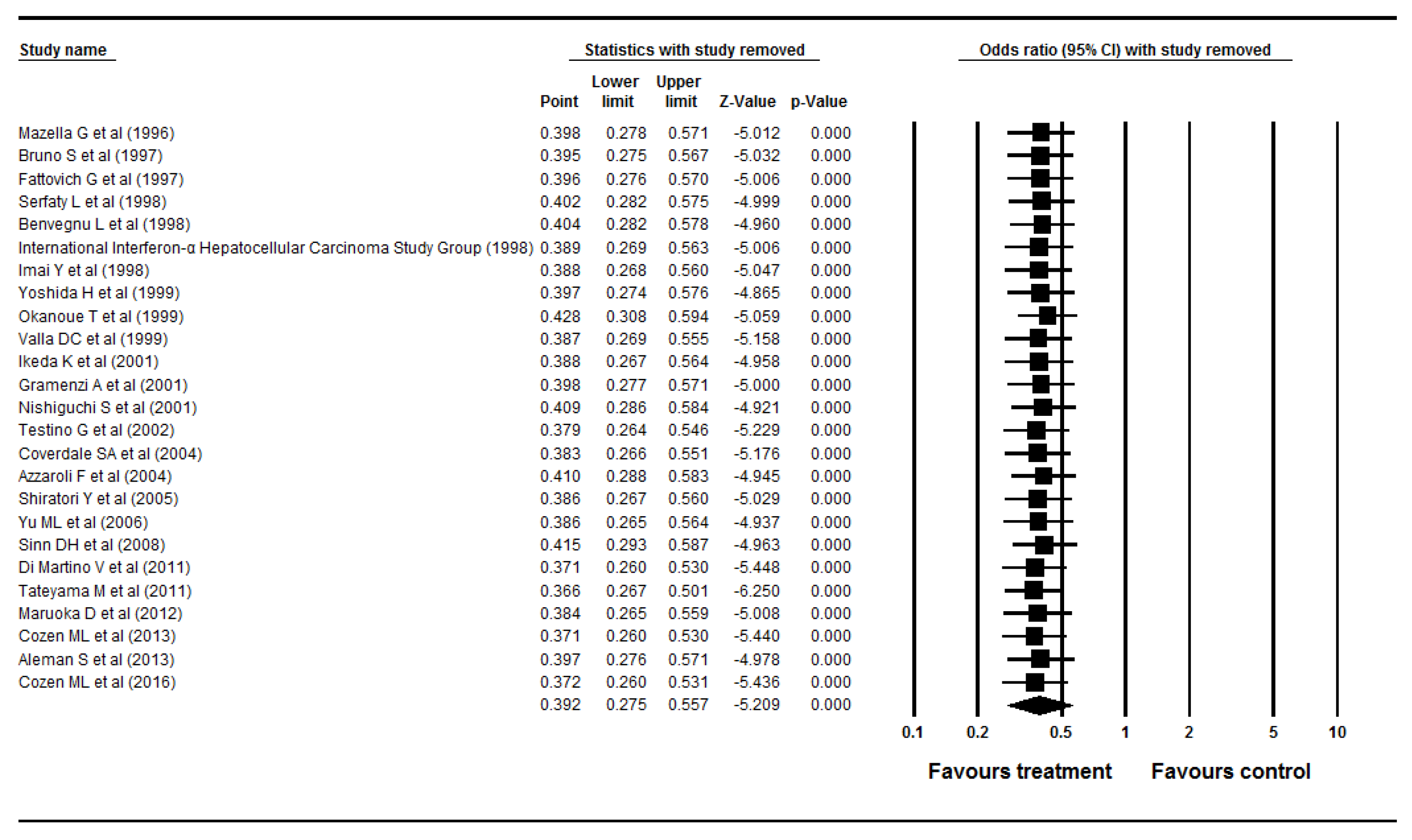
**

Diamond is the summary estimate from the pooled studies with 95% CI (Random effect model). HCC, hepatocellular carcinoma; CI, confidence interval.

**Appendix 6.** Meta-ANOVA according to the modifiers for the efficacy of antiviral treatment on the development of HCC (study format / Nationality / Histology / Follow-up duration / Newcastle-Ottawa scale / Age / Treatment). **
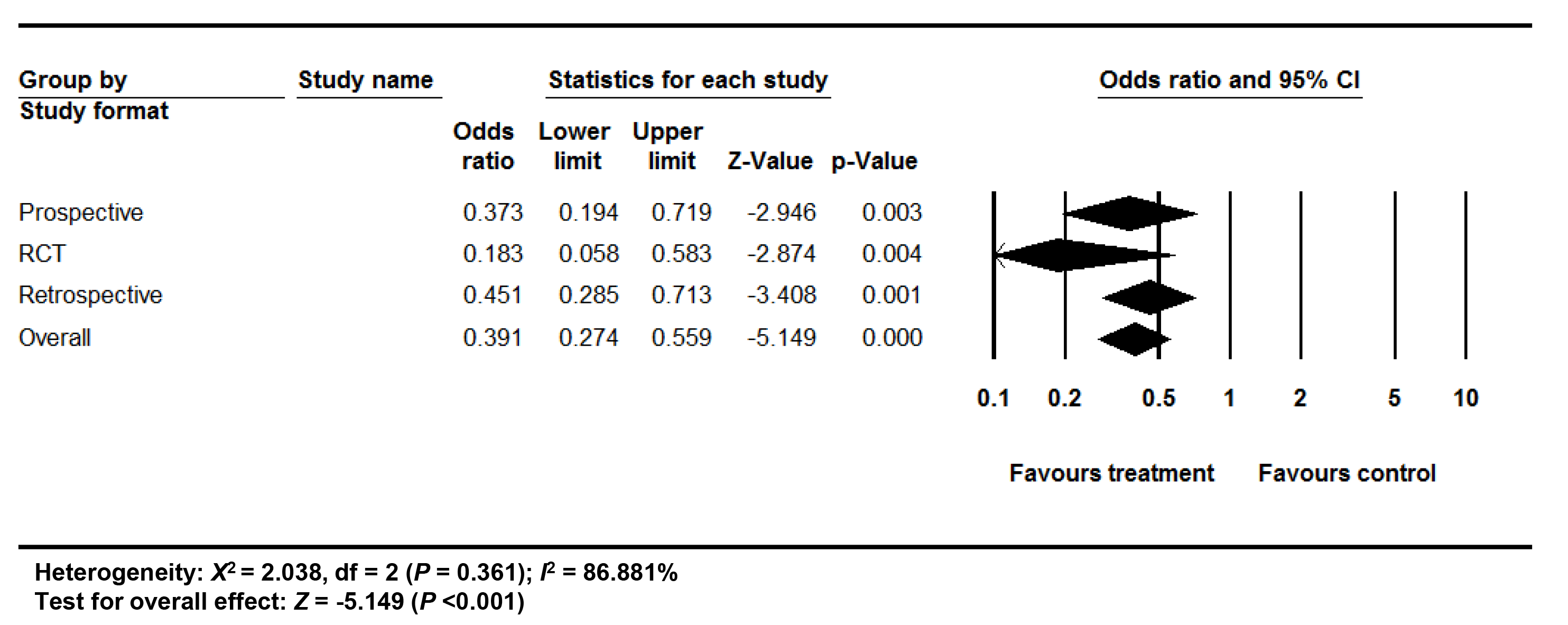
**

**
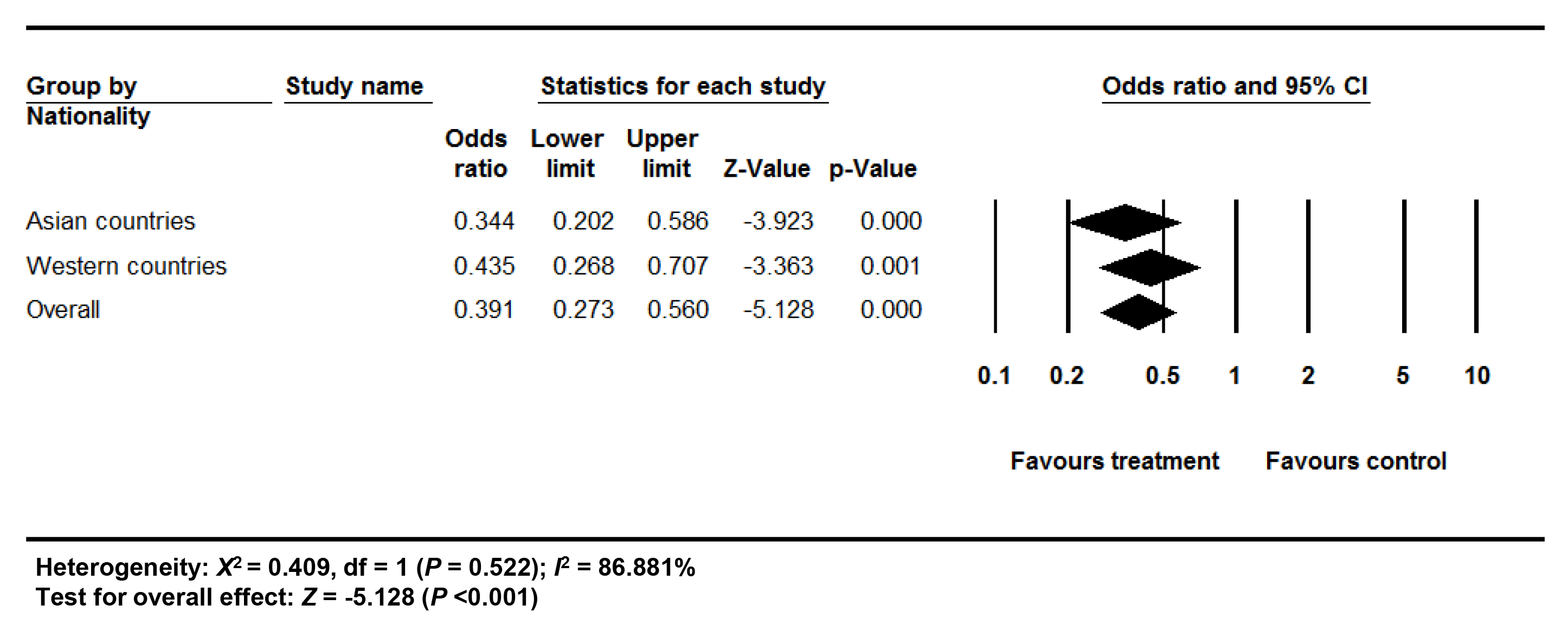
**

**
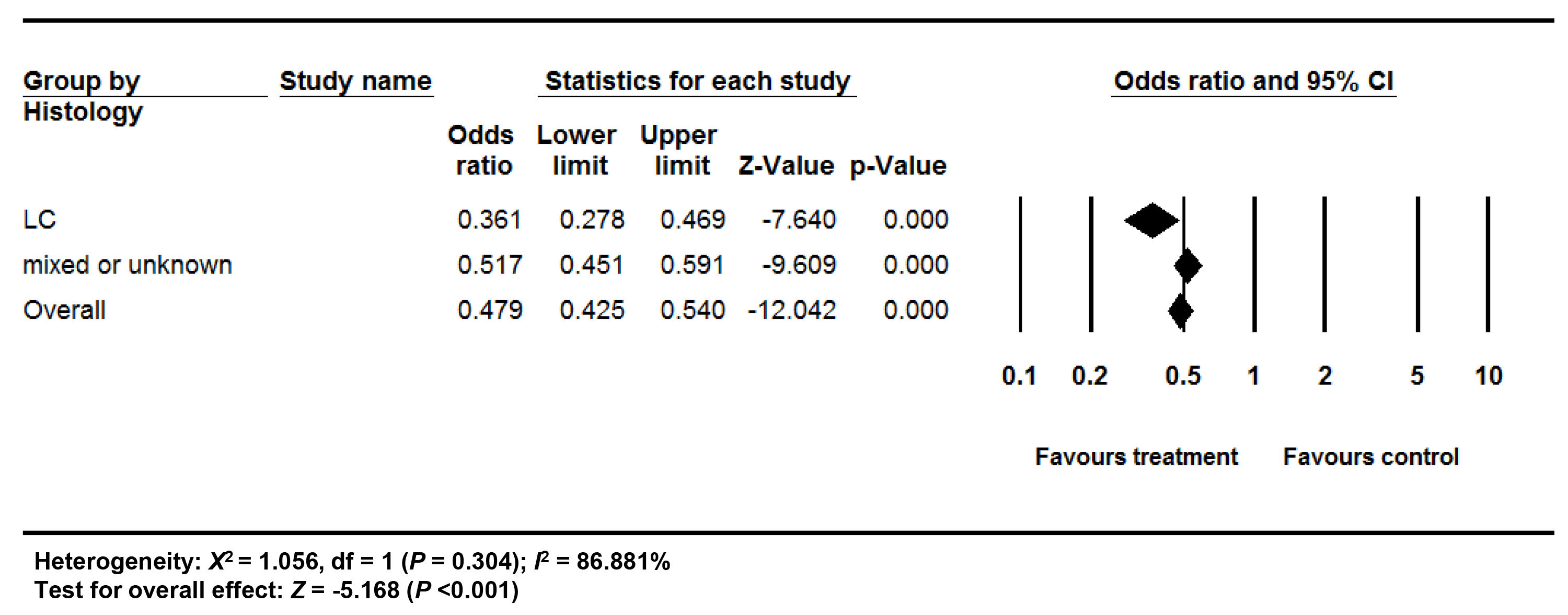
**

**
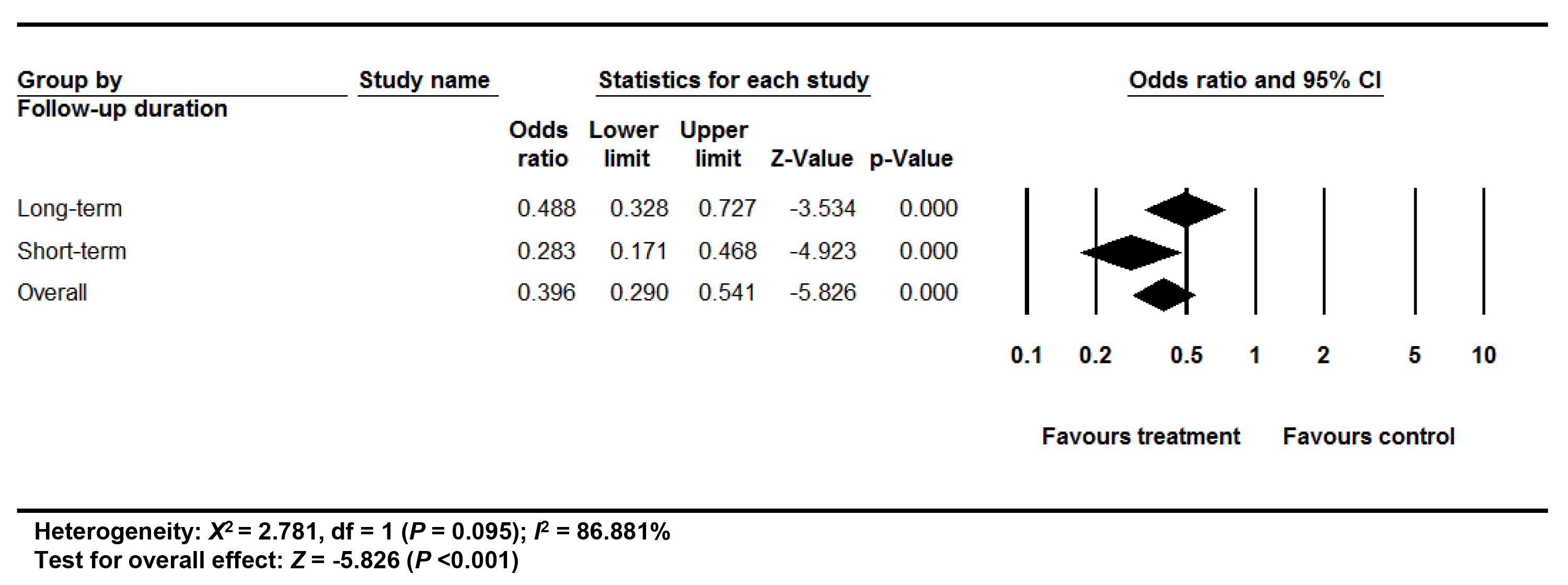
**

**
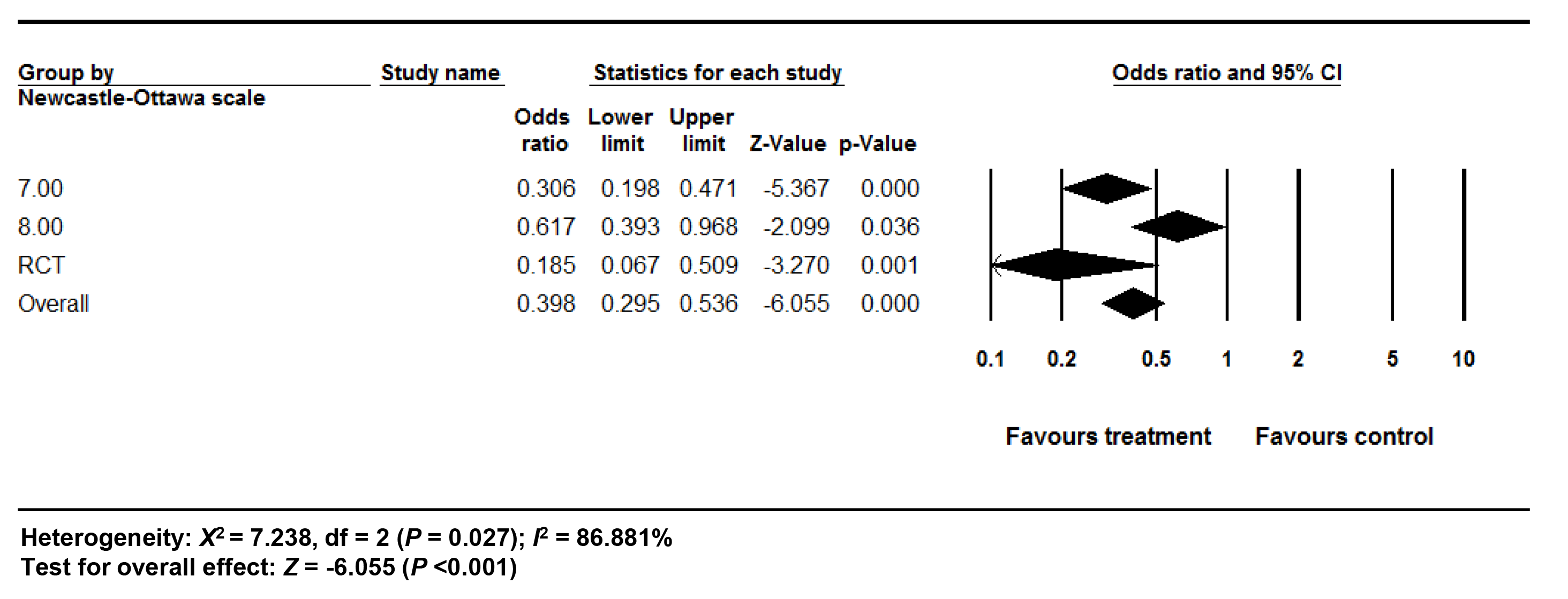
**

**
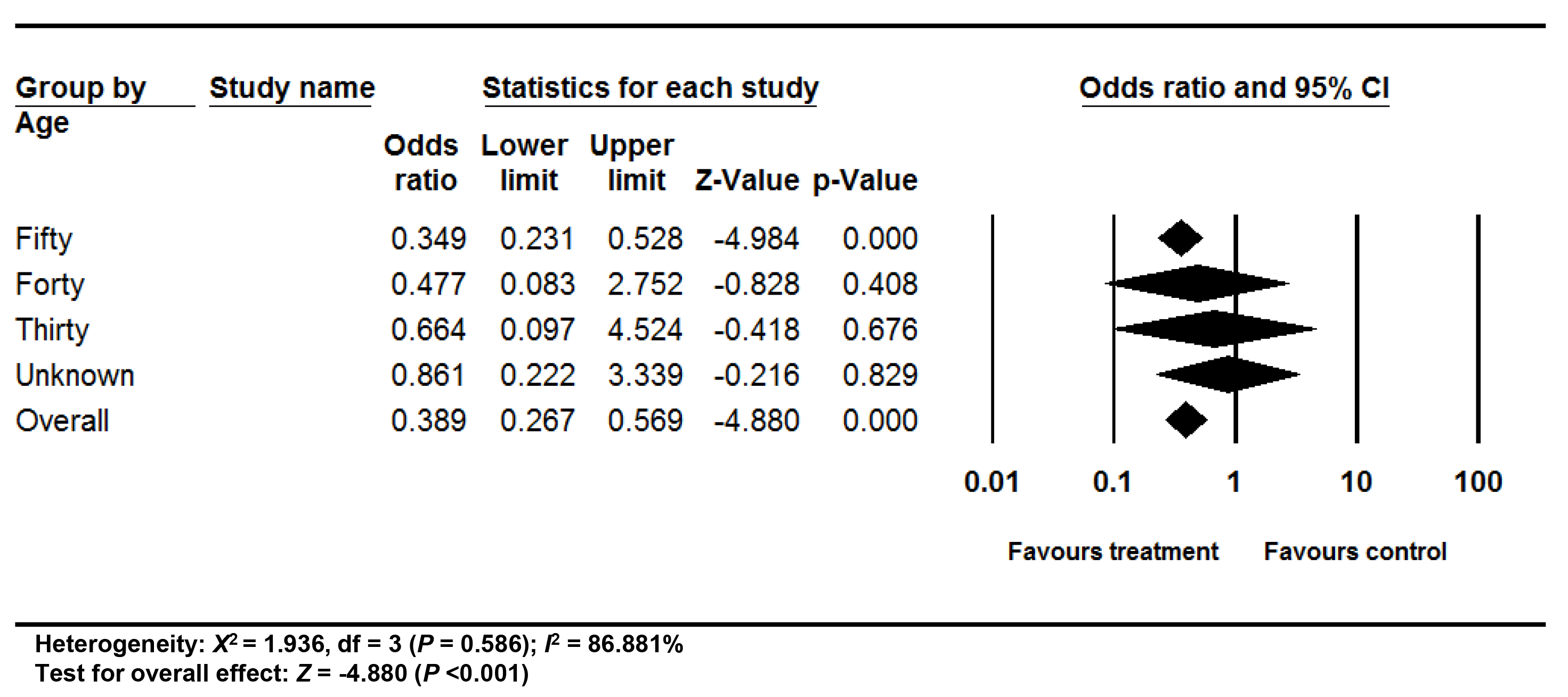
**


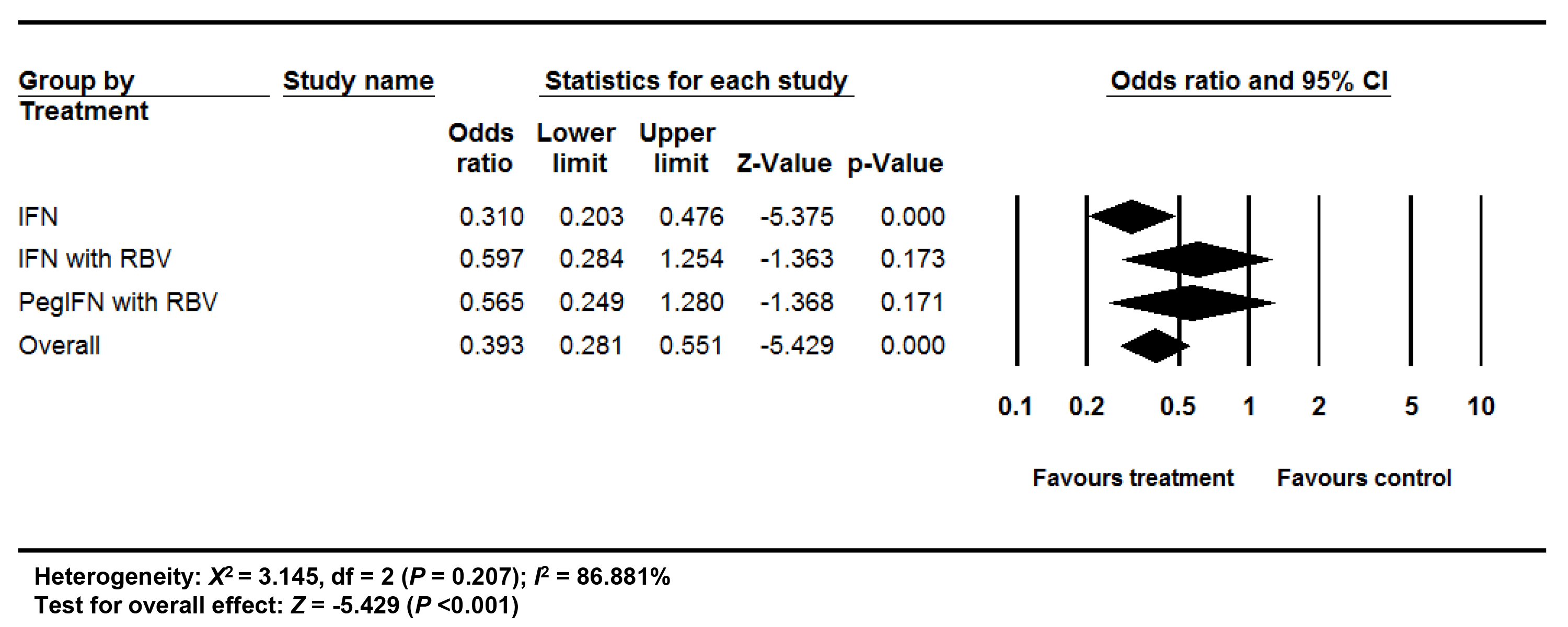


Diamond is the summary estimate from the pooled studies with 95% CI (Mixed effect model). HCC, hepatocellular carcinoma; CI, confidence interval.

**Appendix 7.** Funnel plot of studies for efficacy of antiviral treatment on all-cause mortality.

**
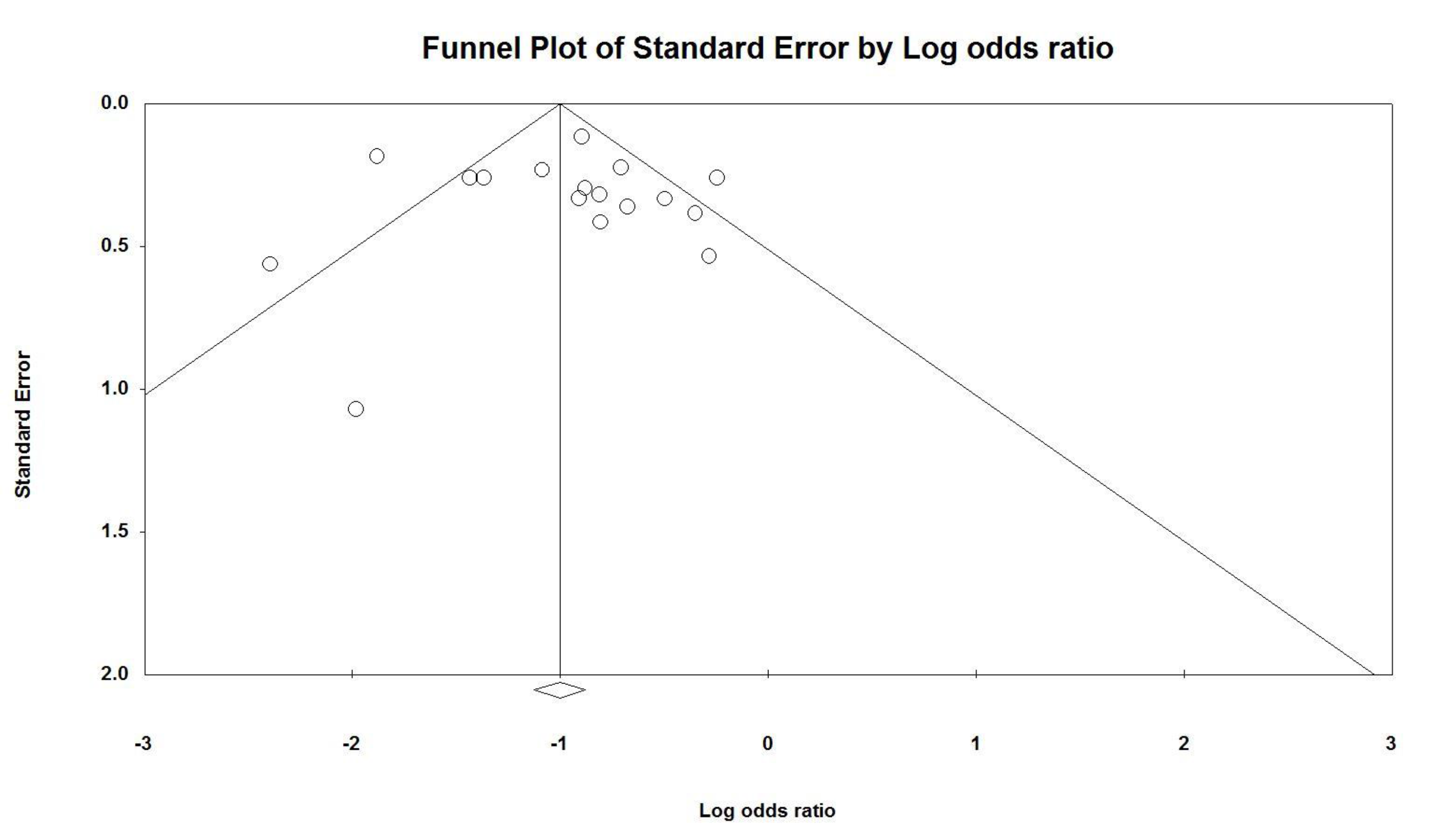
**

The line in center is the natural logarithm of pooled OR, and 2 oblique lines are pseudo 95% confidence limits. OR, odds ratio.

**Appendix 8.** Cumulative meta-analysis of enrolled studies for the efficacy of antiviral treatment on all-cause mortality (based on publication year).

**
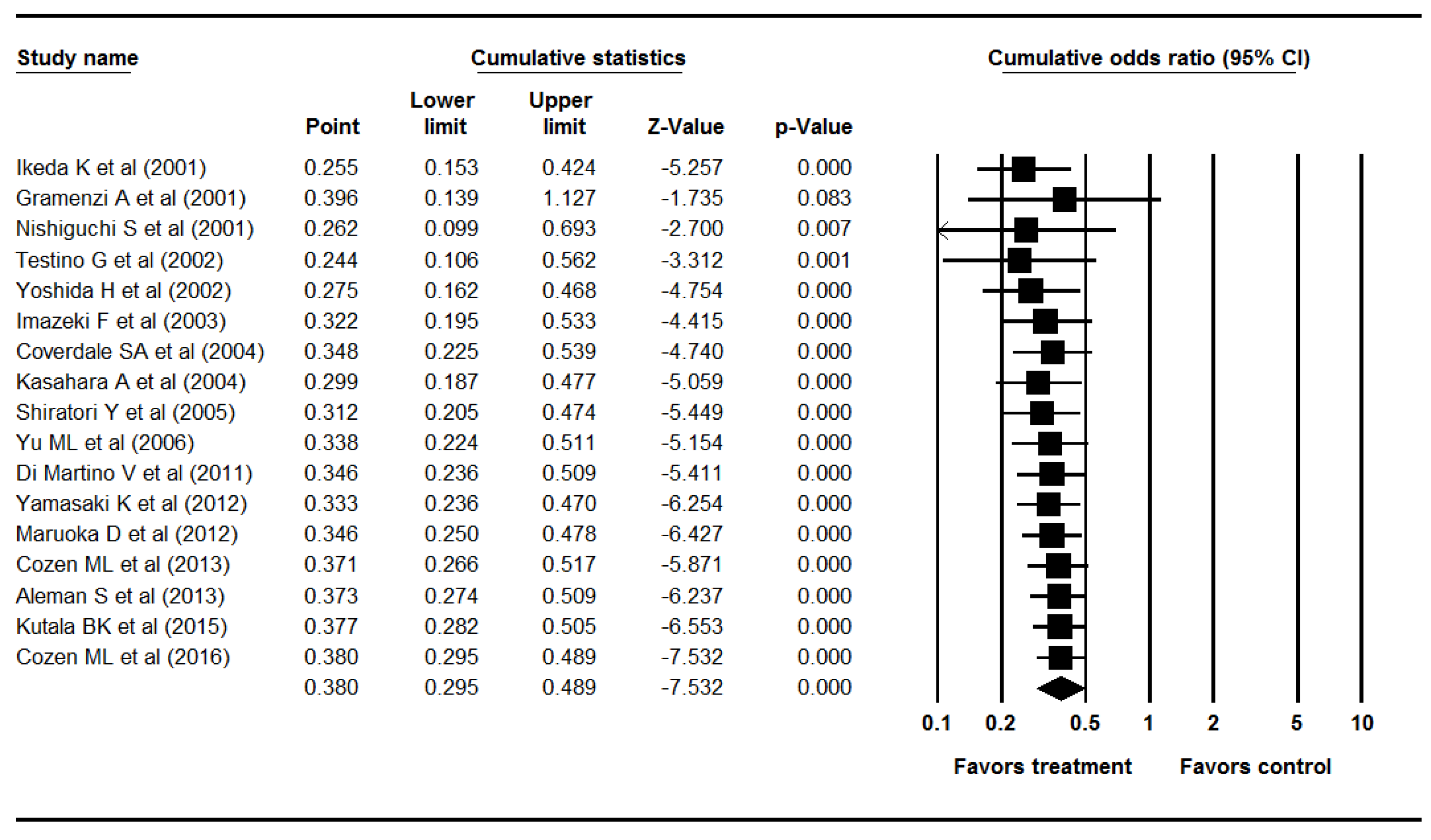
**

Diamond is the summary estimate from the pooled studies with 95% CI (Random effect model). CI, confidence interval.

**Appendix 9.** Cumulative meta-analysis of enrolled studies for the efficacy of antiviral treatment on all-cause mortality (based on effect size).

**
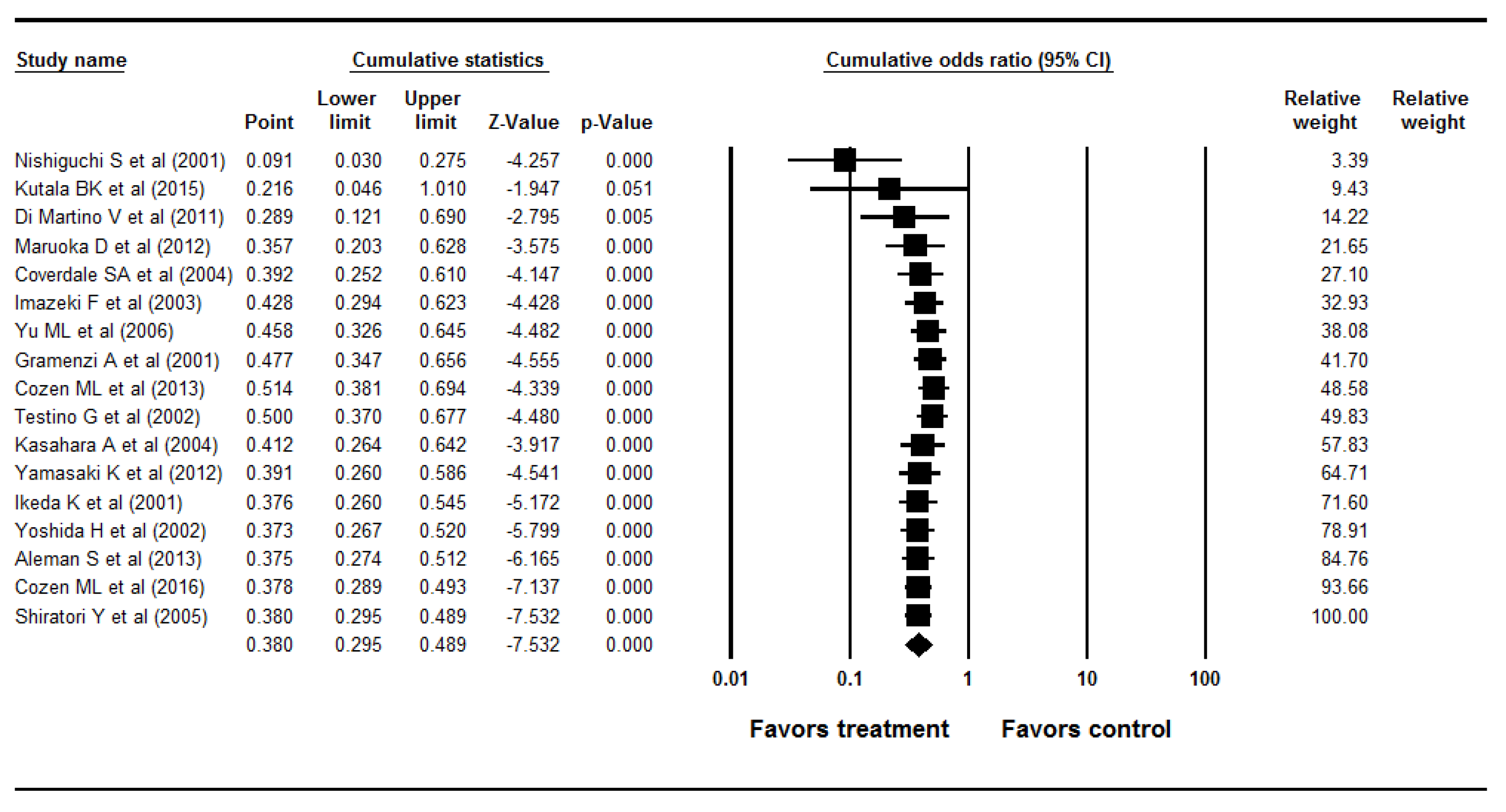
**

Diamond is the summary estimate from the pooled studies with 95% CI (Random effect model). CI, confidence interval.

**Appendix 10.** One study removed meta-analysis of enrolled studies for the efficacy of antiviral treatment on all-cause mortality.

**
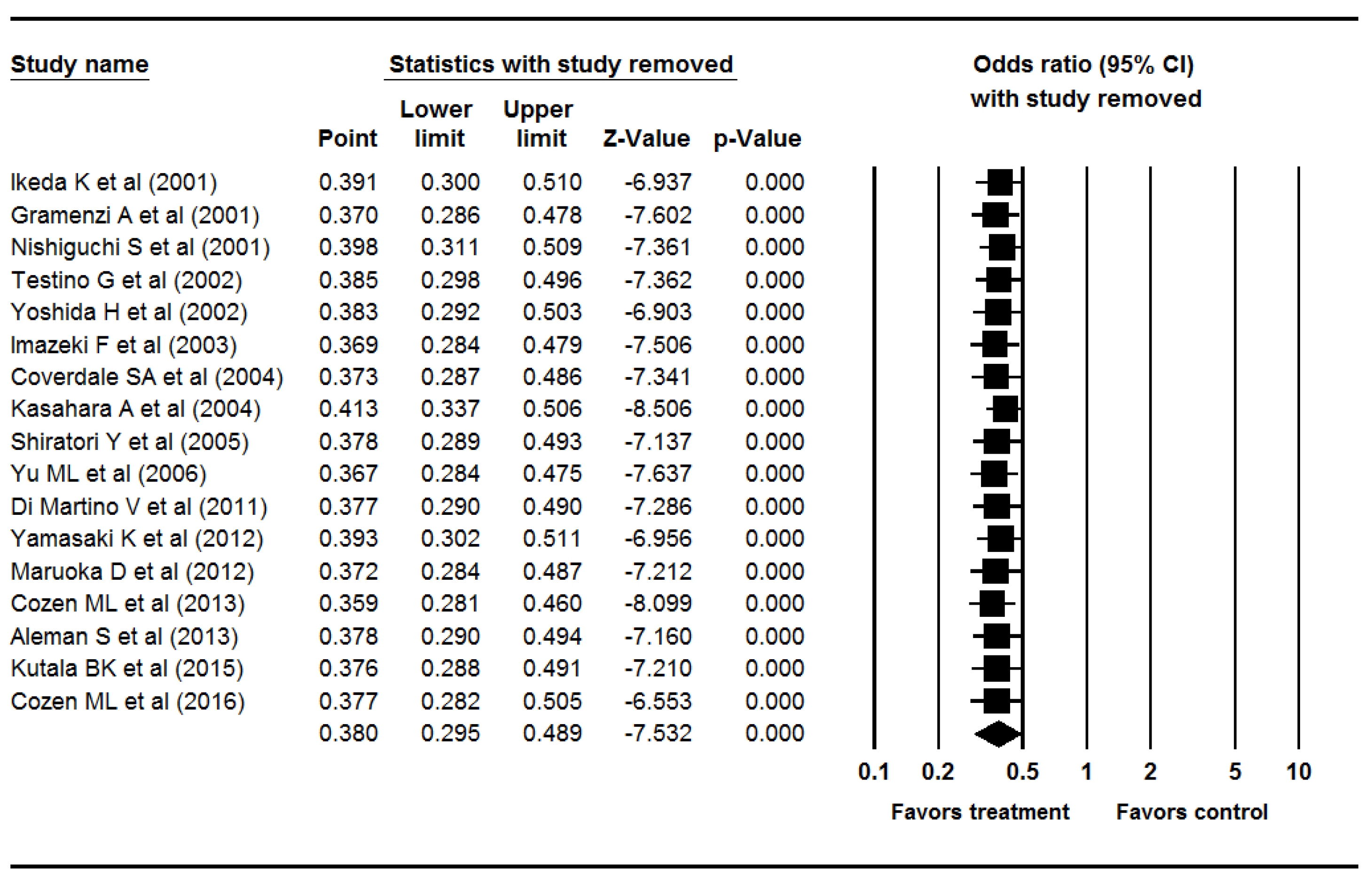
**

Diamond is the summary estimate from the pooled studies with 95% CI (Random effect model). CI, confidence interval.

**Appendix 11.** Meta-ANOVA according to the modifiers for the efficacy of antiviral treatment on all-cause mortality (study format / Nationality / Histology / Follow-up duration / Newcastle-Ottawa scale / Age / Treatment).

**
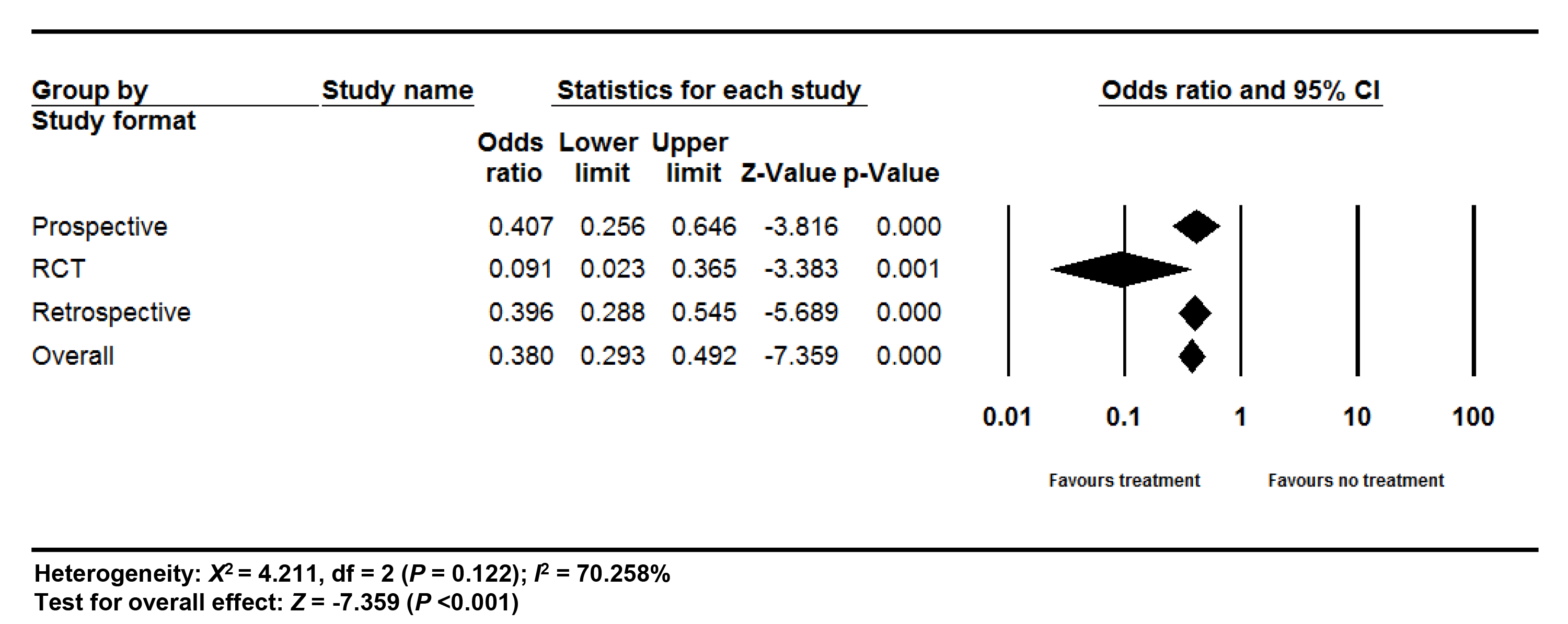
**

**
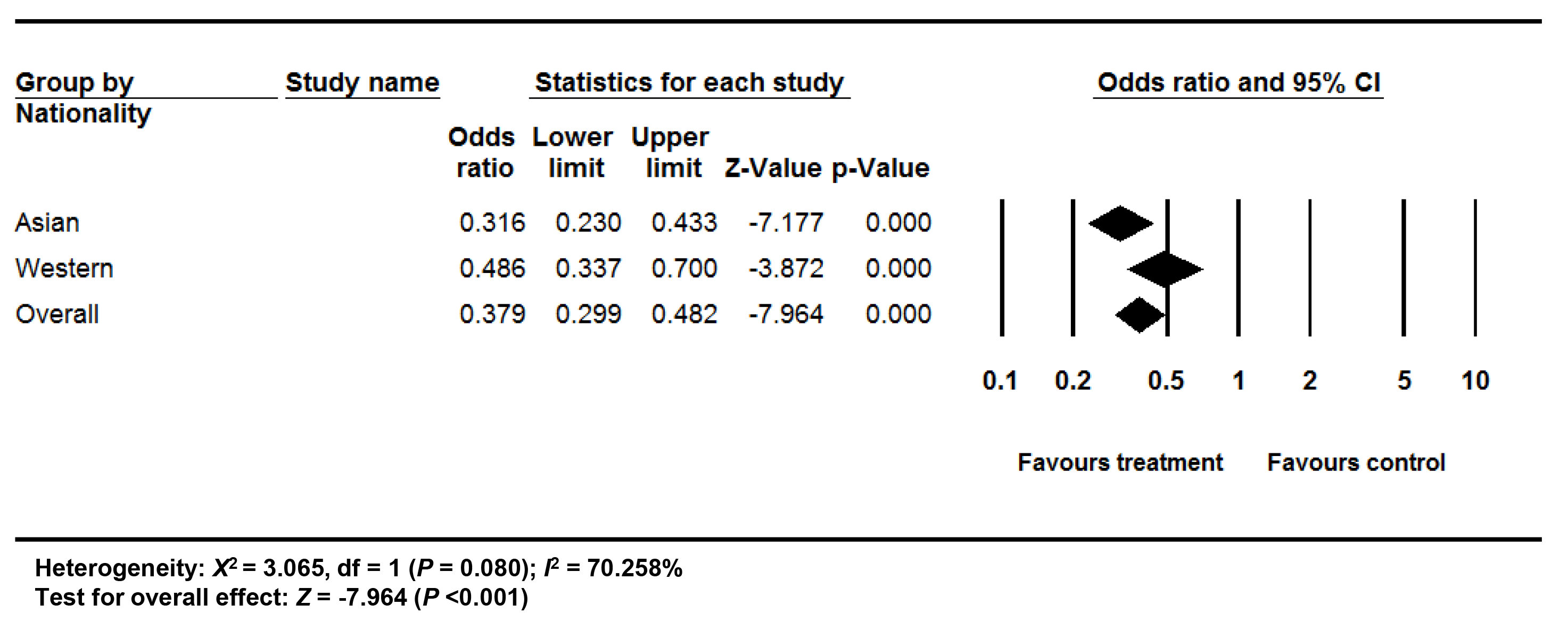
**

**
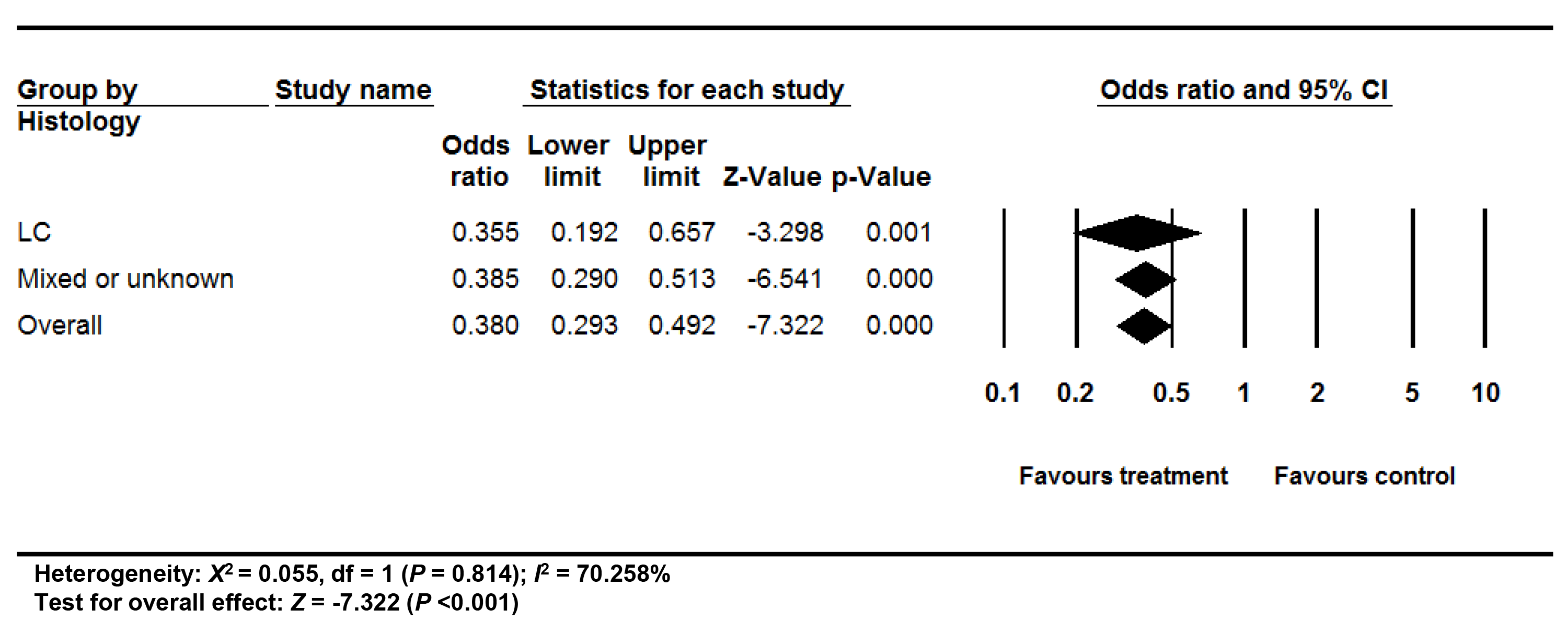
**

**
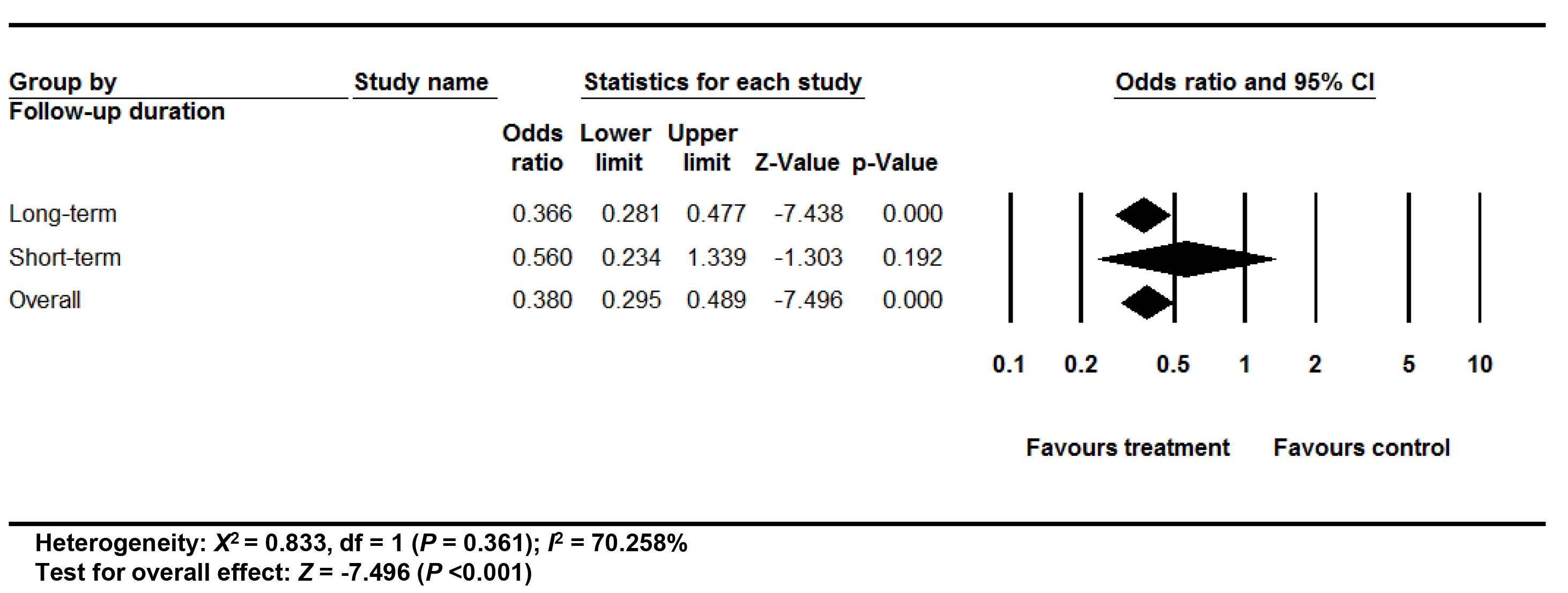
**

**
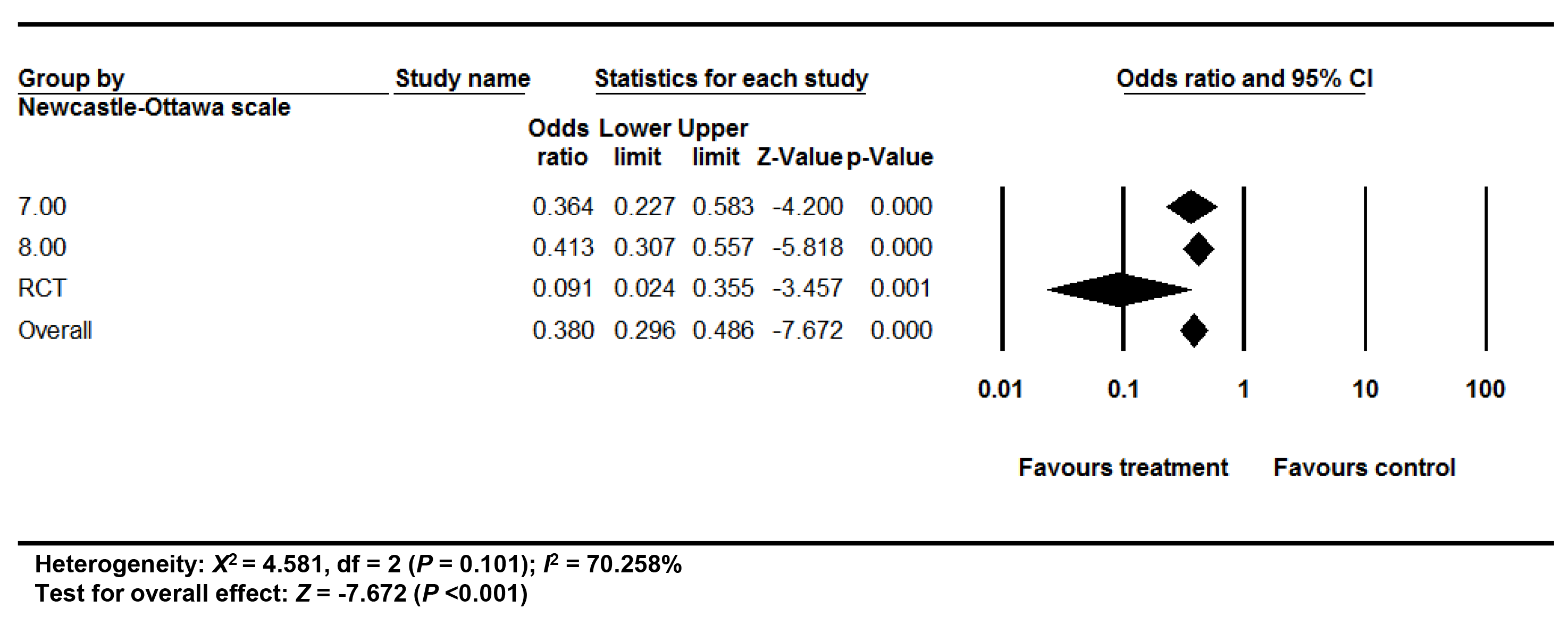
**

**
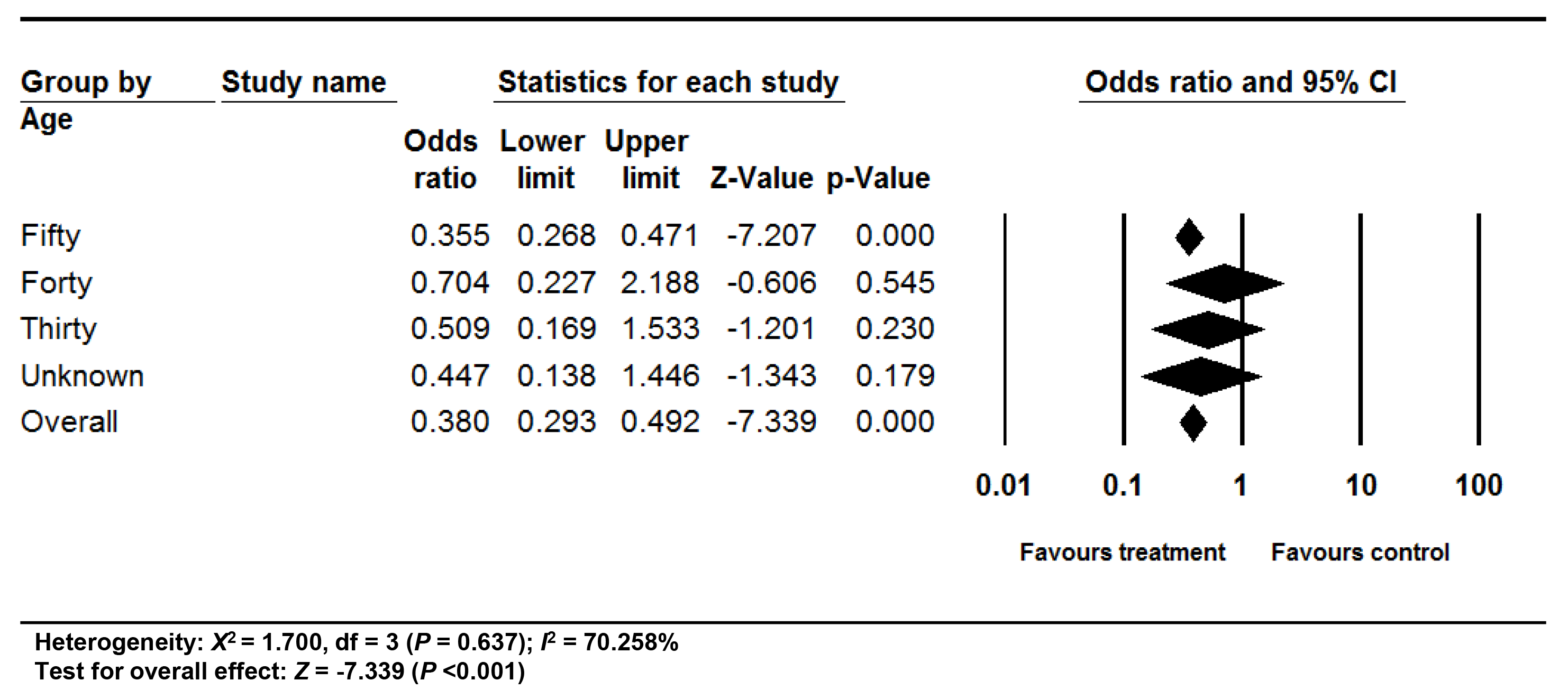
**


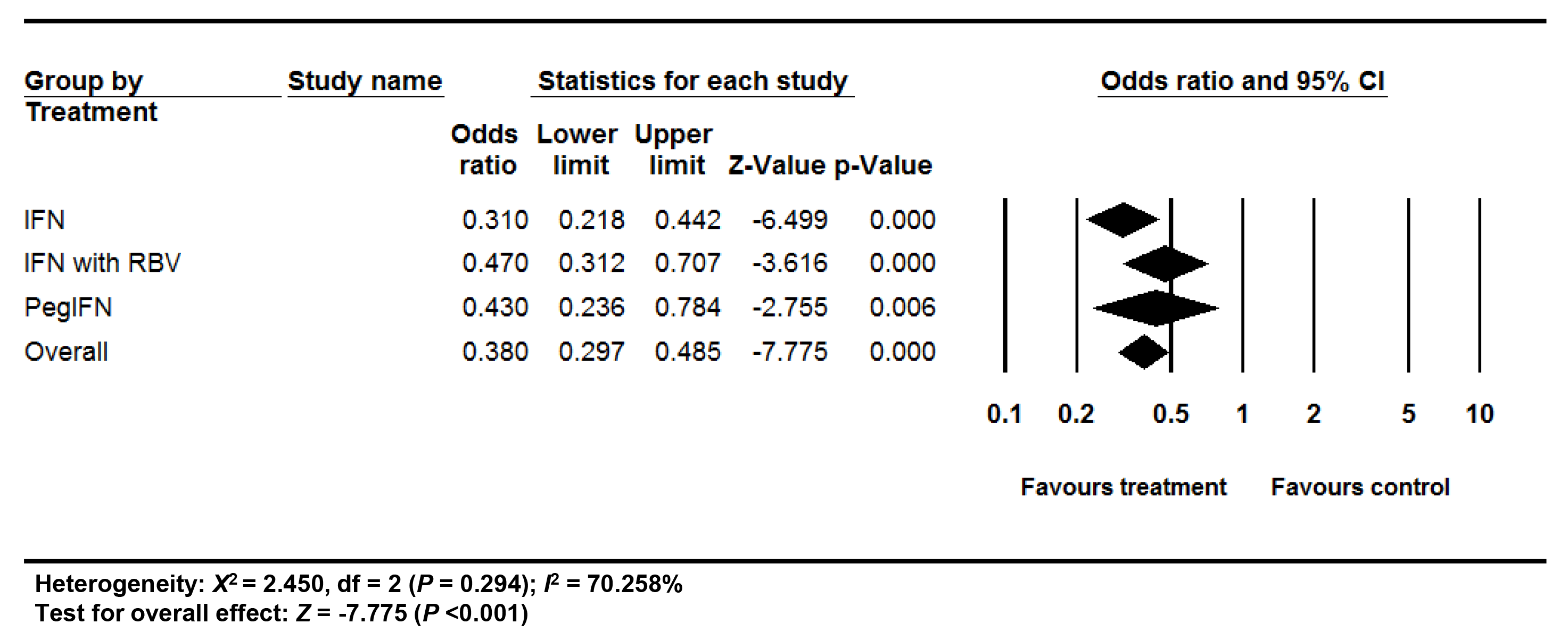


Diamond is the summary estimate from the pooled studies with 95% CI (Mixed effect model). CI, confidence interval.

**Appendix 12.** Efficacy of antiviral treatment on liver-specific mortality in patients with CHC.


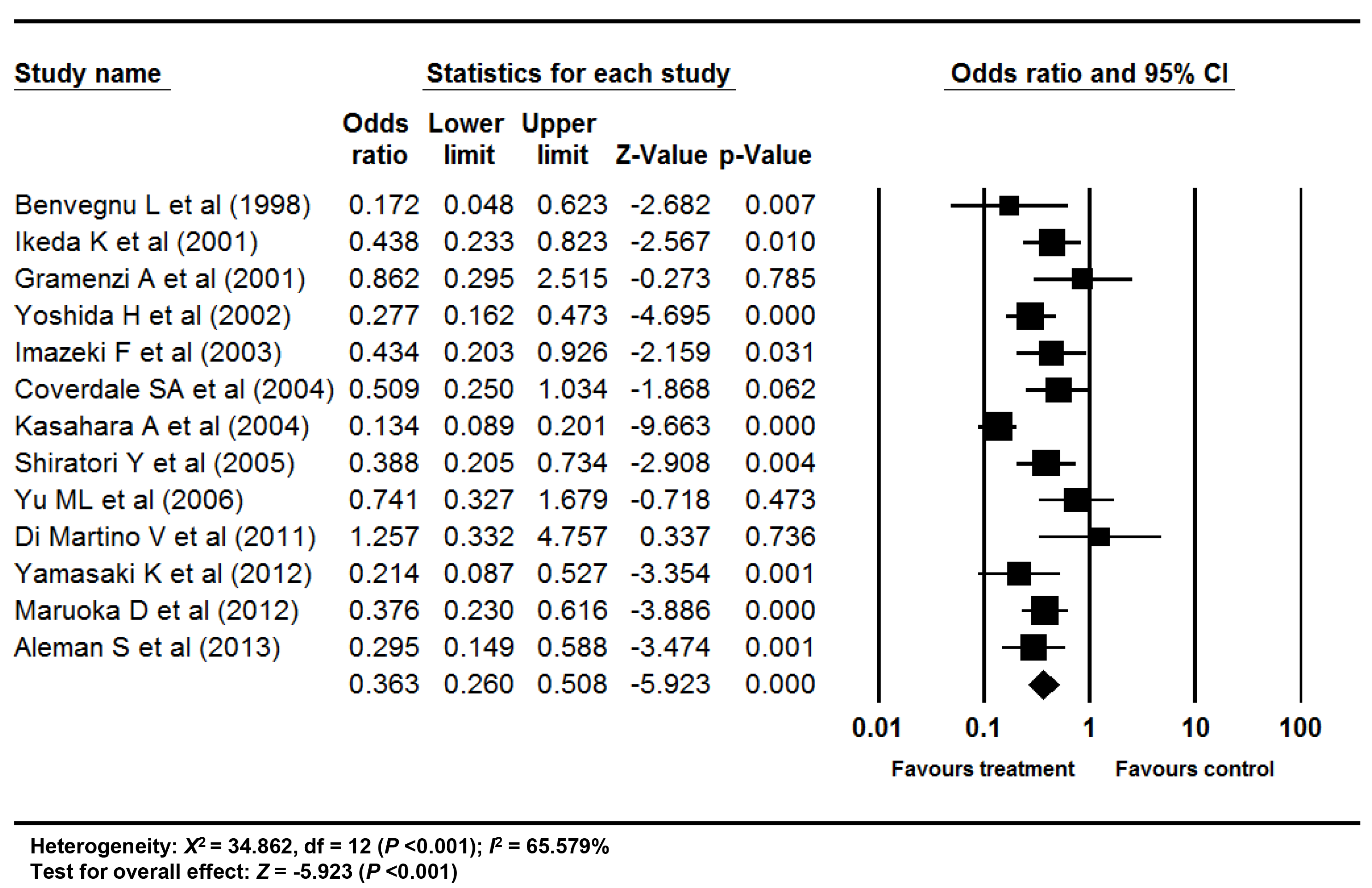


The size of each square is proportional to the study’s weight. Diamond is the summary estimate from the pooled studies (random effect model). CHC, chronic hepatitis C.

**Appendix 13.** Funnel plot of studies for efficacy of antiviral treatment on liver-specific mortality.

**
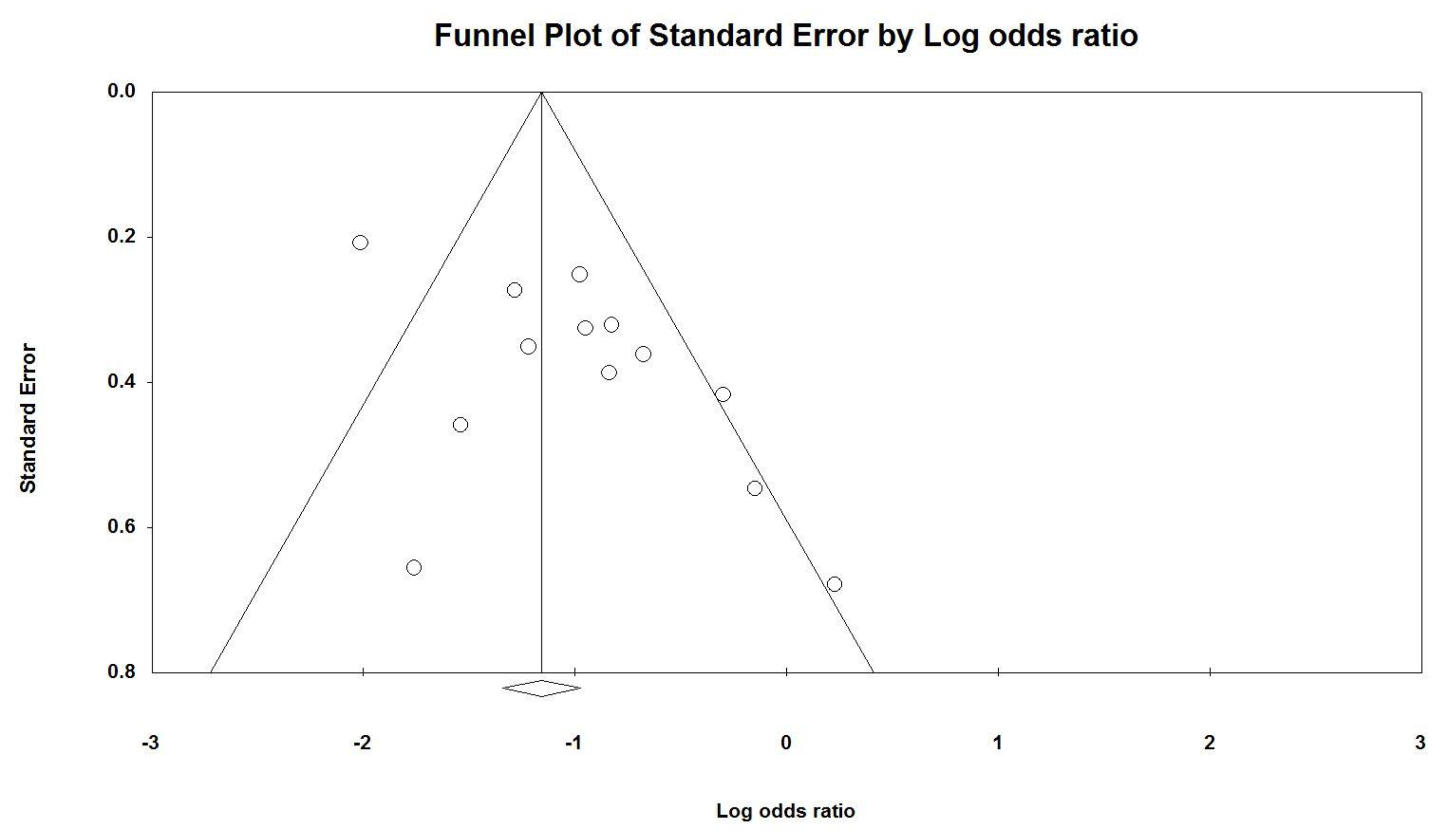
**

The line in center is the natural logarithm of pooled OR, and 2 oblique lines are pseudo 95% confidence limits. OR, odds ratio.

**Appendix 14.** Cumulative meta-analysis of enrolled studies for the efficacy of antiviral treatment on liver-specific mortality (based on publication year).

**
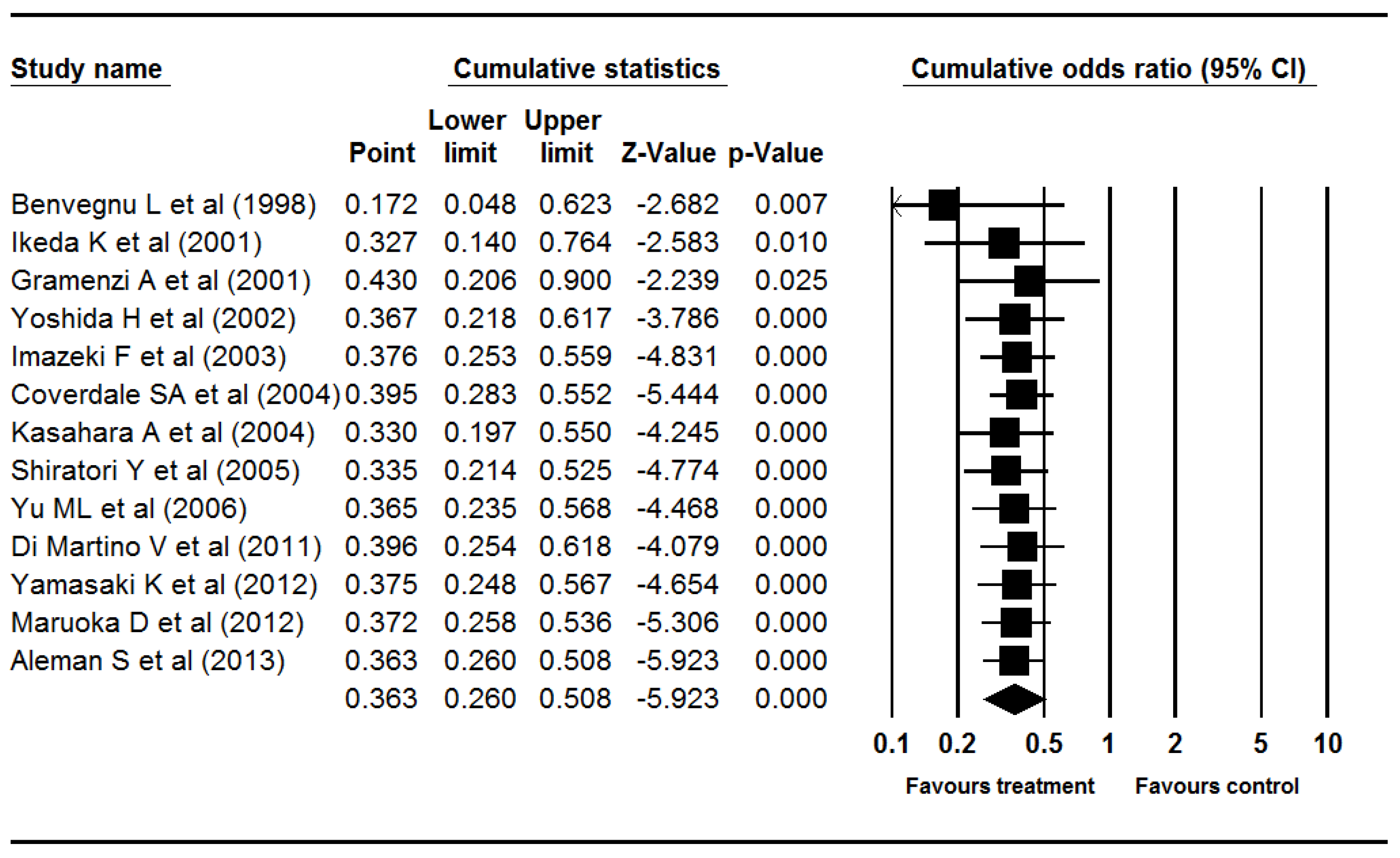
**

Diamond is the summary estimate from the pooled studies with 95% CI (Random effect model). CI, confidence interval.

**Appendix 15.** Cumulative meta-analysis of enrolled studies for the efficacy of antiviral treatment on liver-specific mortality (based on effect size).

**
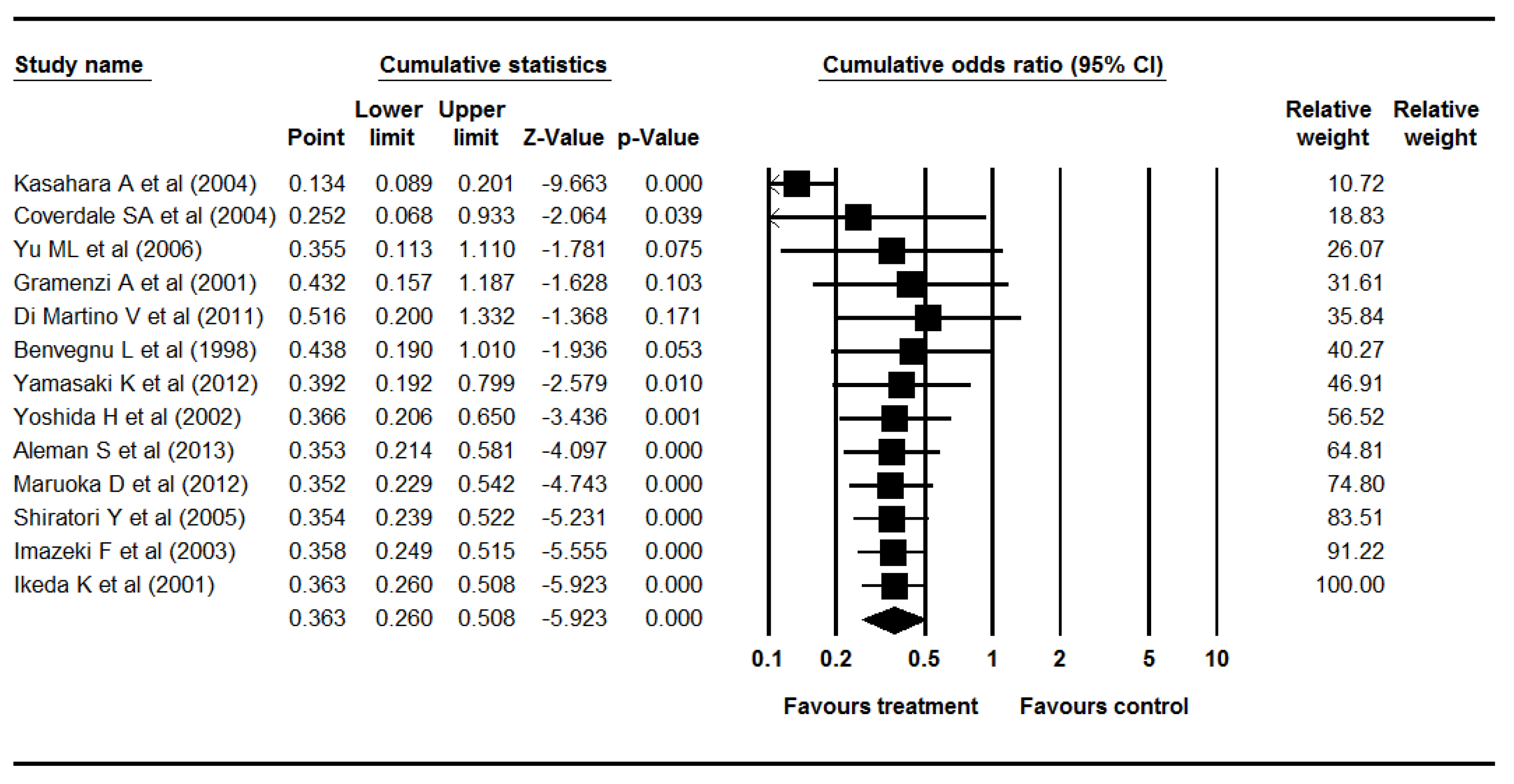
**

Diamond is the summary estimate from the pooled studies with 95% CI (Random effect model). CI, confidence interval.

**Appendix 16.** One study removed meta-analysis of enrolled studies for the efficacy of antiviral treatment on liver-specific mortality.

**
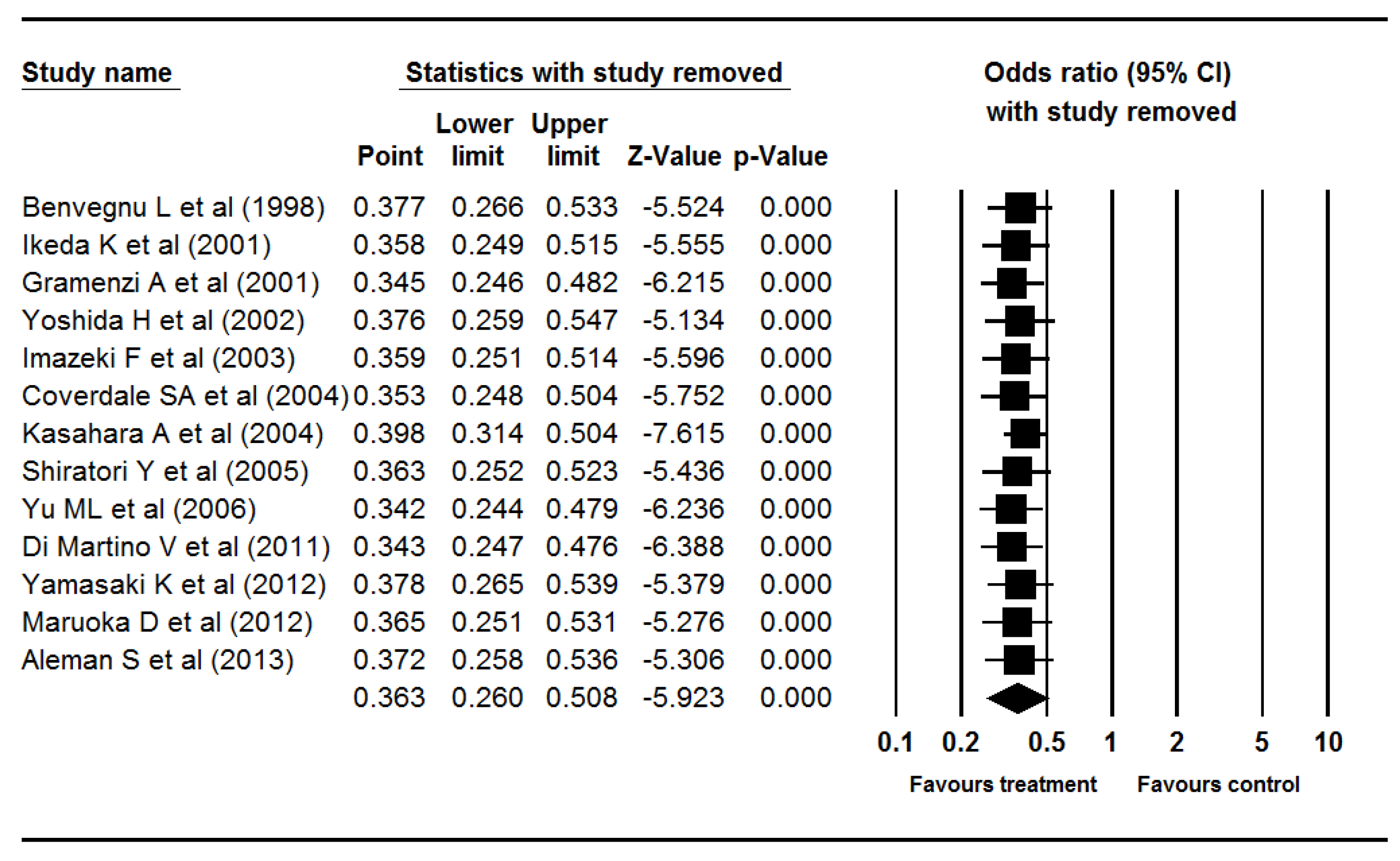
**

Diamond is the summary estimate from the pooled studies with 95% CI (Random effect model). CI, confidence interval.

**Appendix 17.** Meta-ANOVA according to the modifiers for the efficacy of antiviral treatment on liver-specific mortality (study format / Nationality / Histology / Follow-up duration / Newcastle-Ottawa scale / Age / Treatment).

**
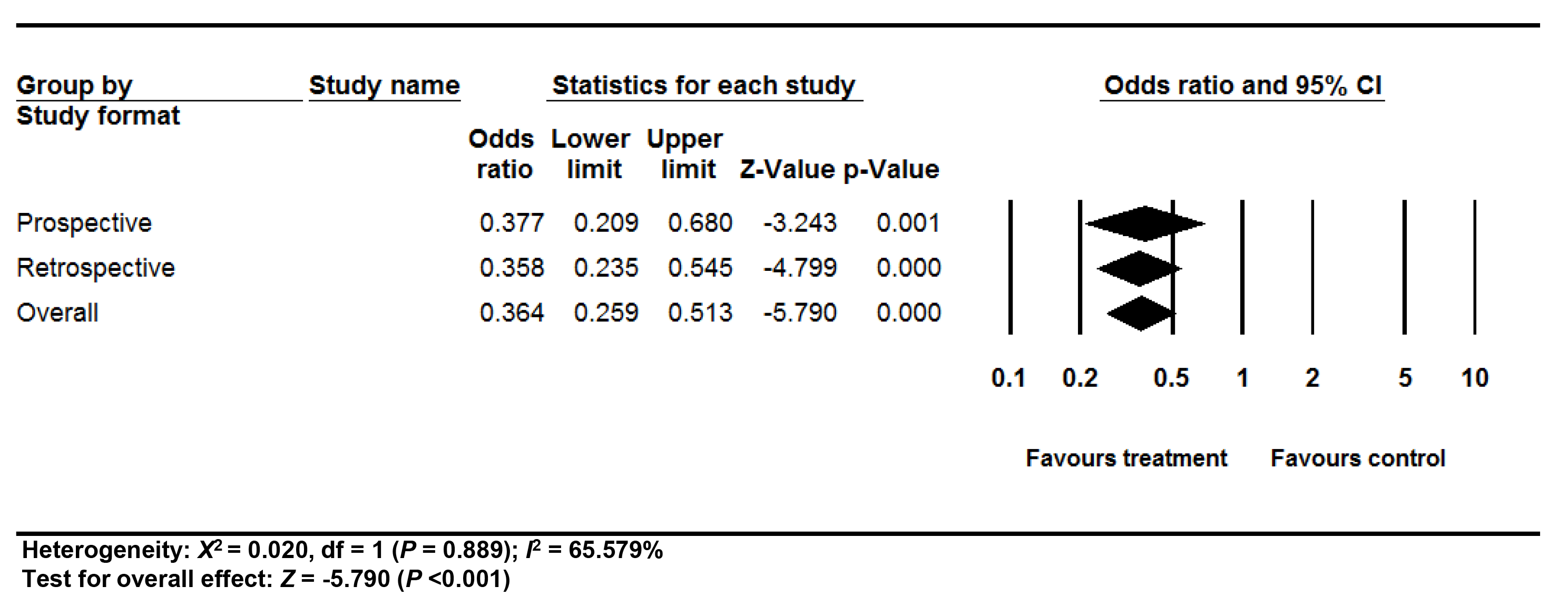
**

**
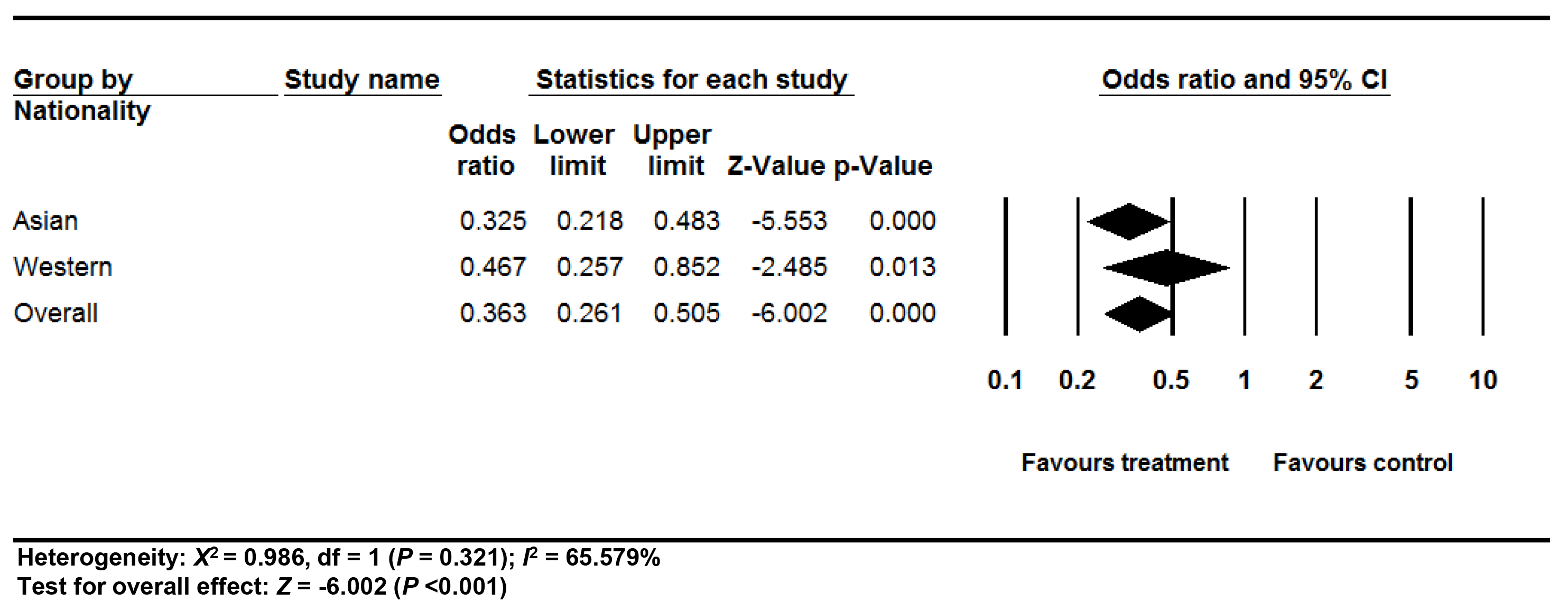
**

**
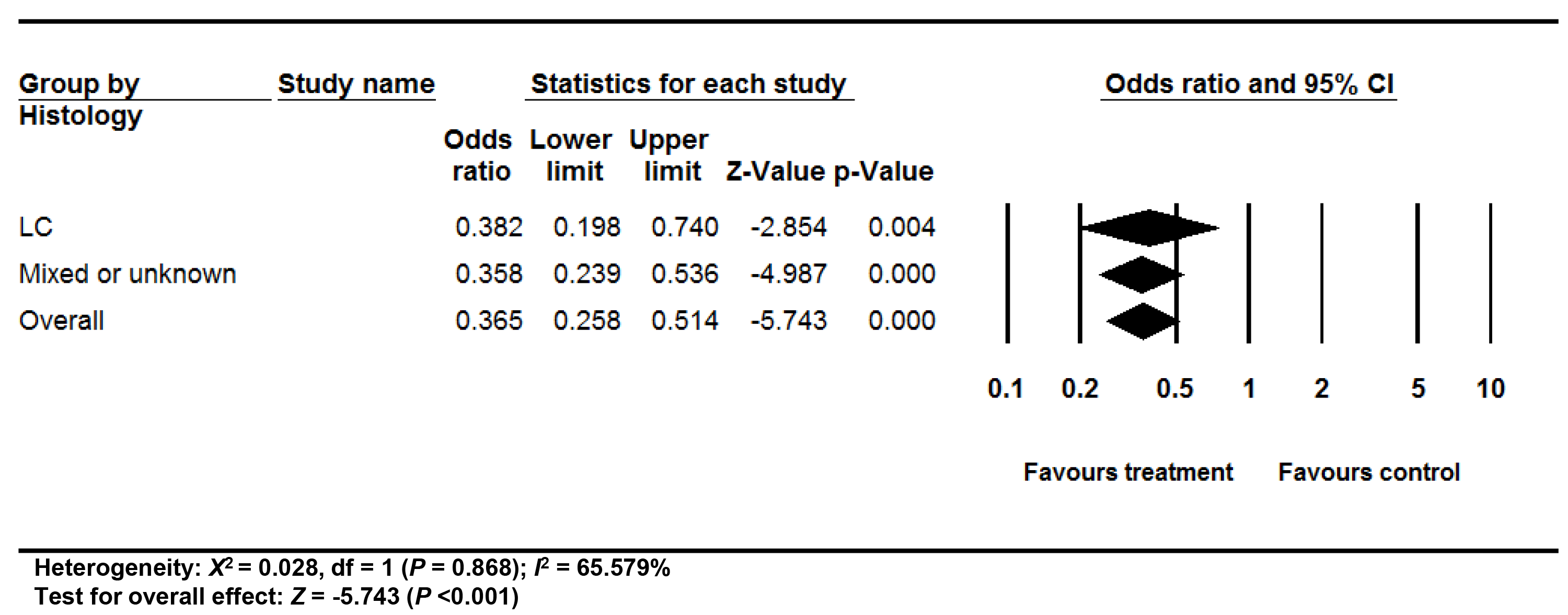
**


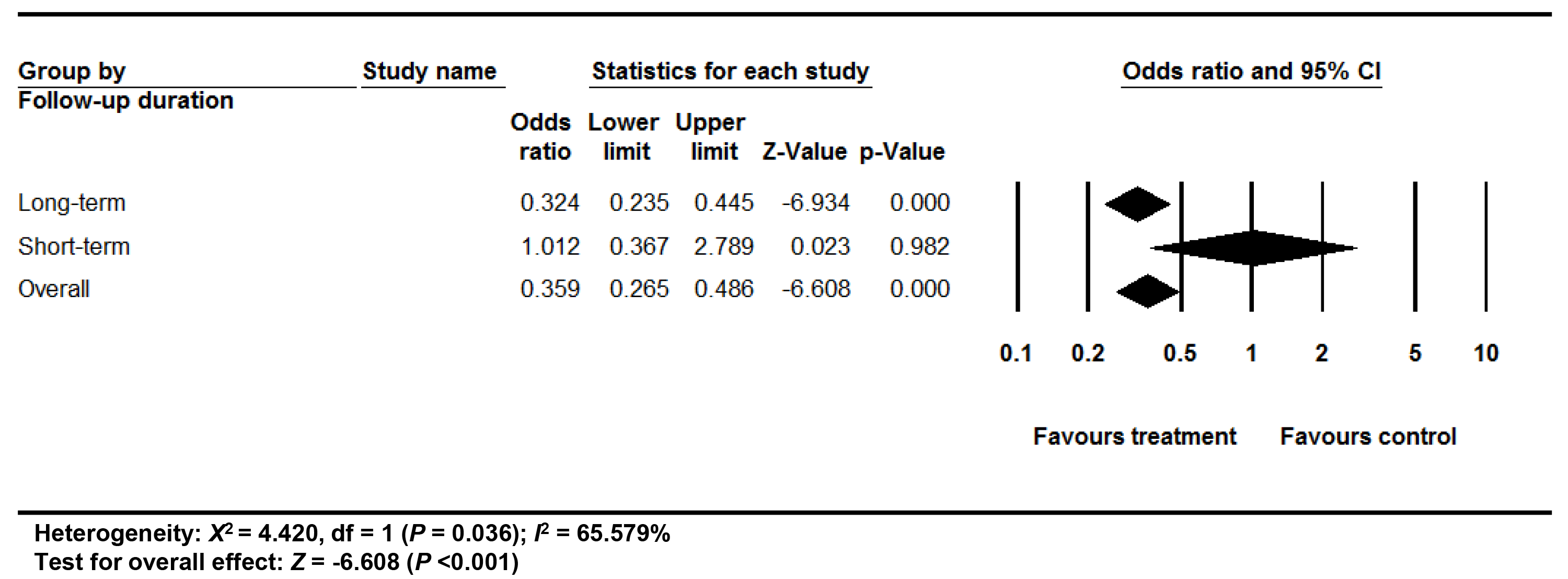


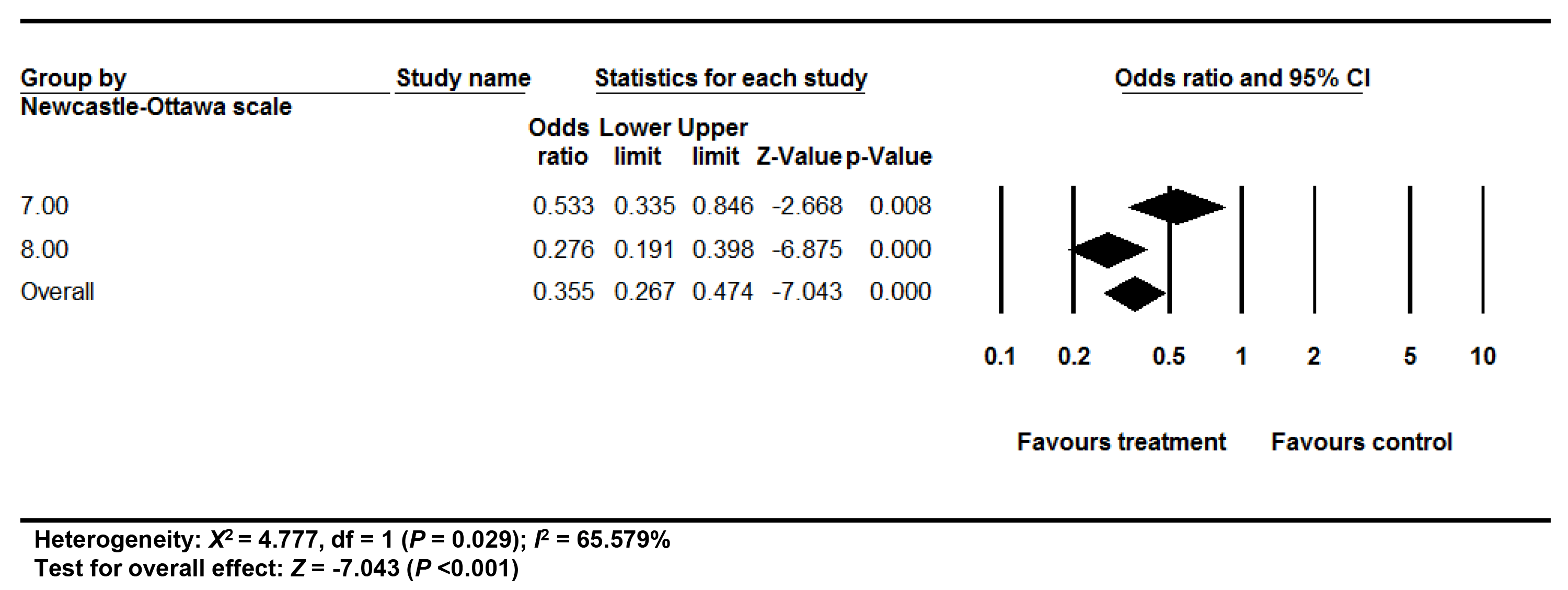


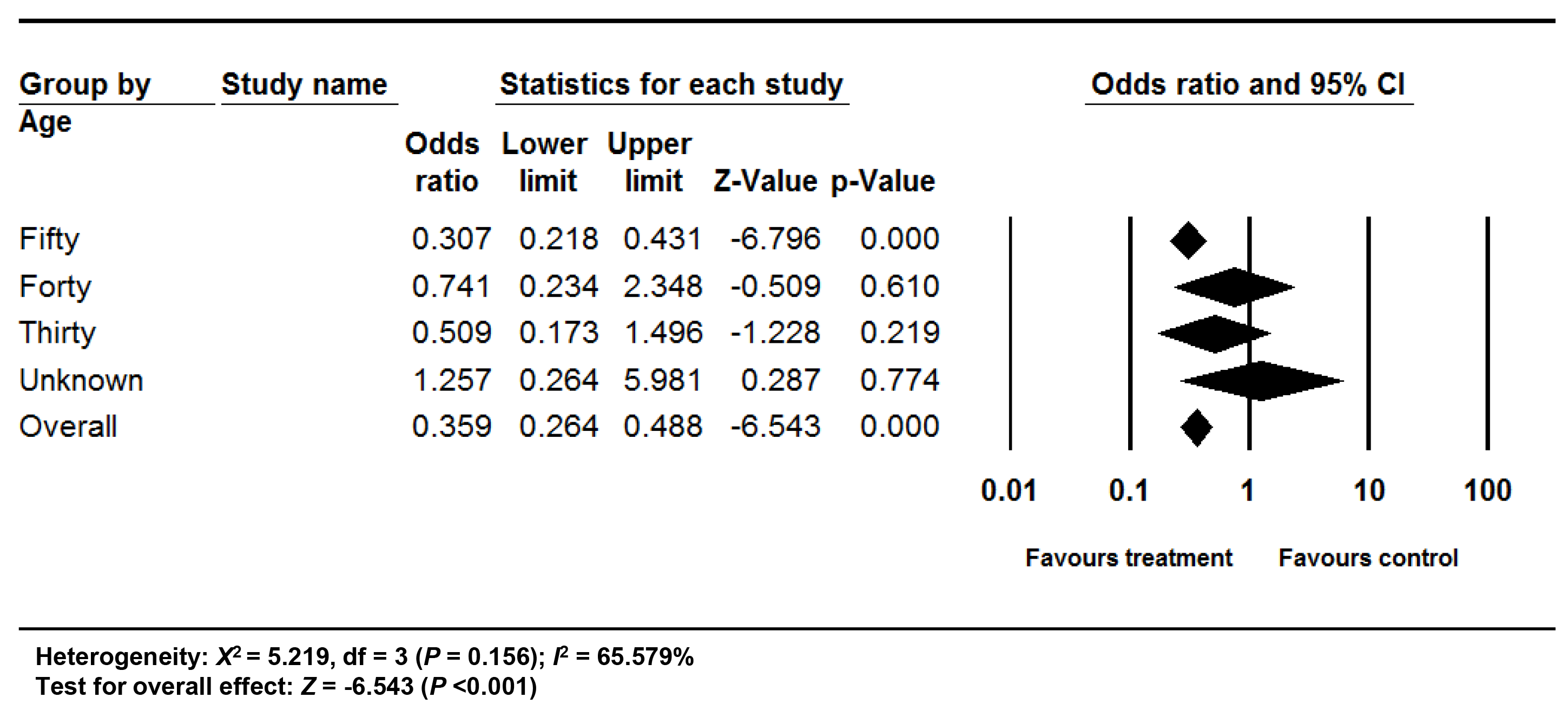


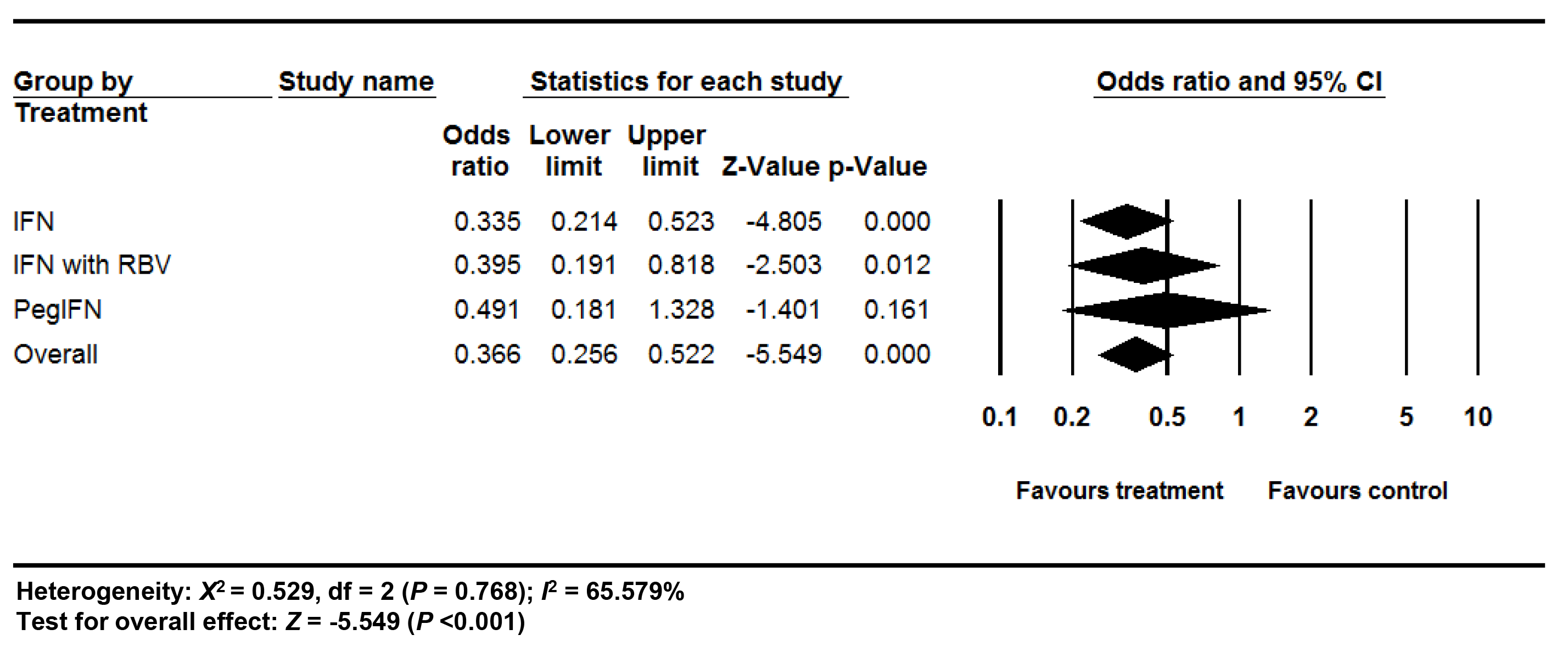


Diamond is the summary estimate from the pooled studies with 95% CI (Mixed effect model). CI, confidence interval.

**Appendix 18.** Funnel plot of studies for efficacy of SVR on the development of HCC.

**
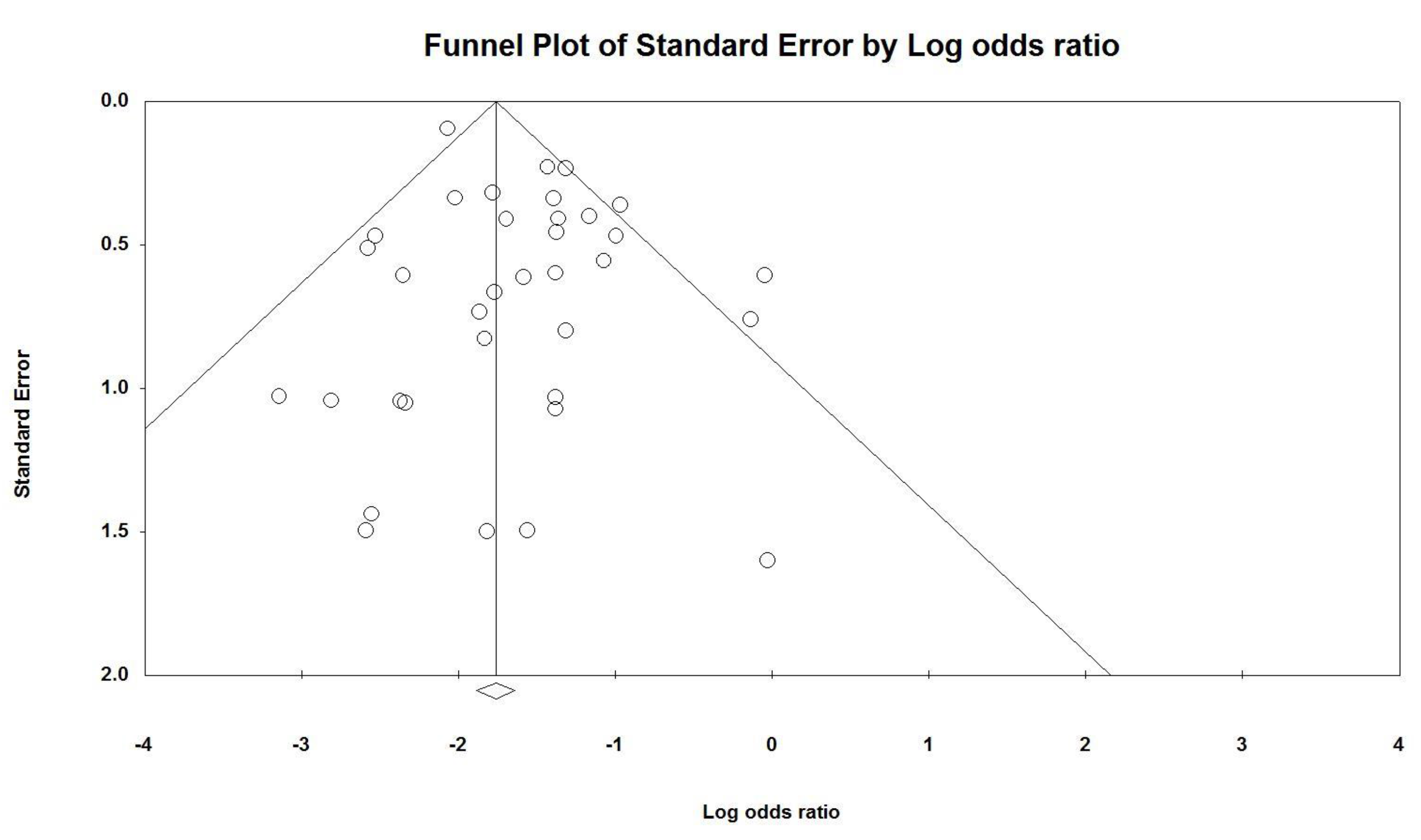
**

The line in center is the natural logarithm of pooled OR, and 2 oblique lines are pseudo 95% confidence limits. OR, odds ratio.

**Appendix 19.** Cumulative meta-analysis of enrolled studies for the efficacy of SVR on the development of HCC (based on publication year).

**
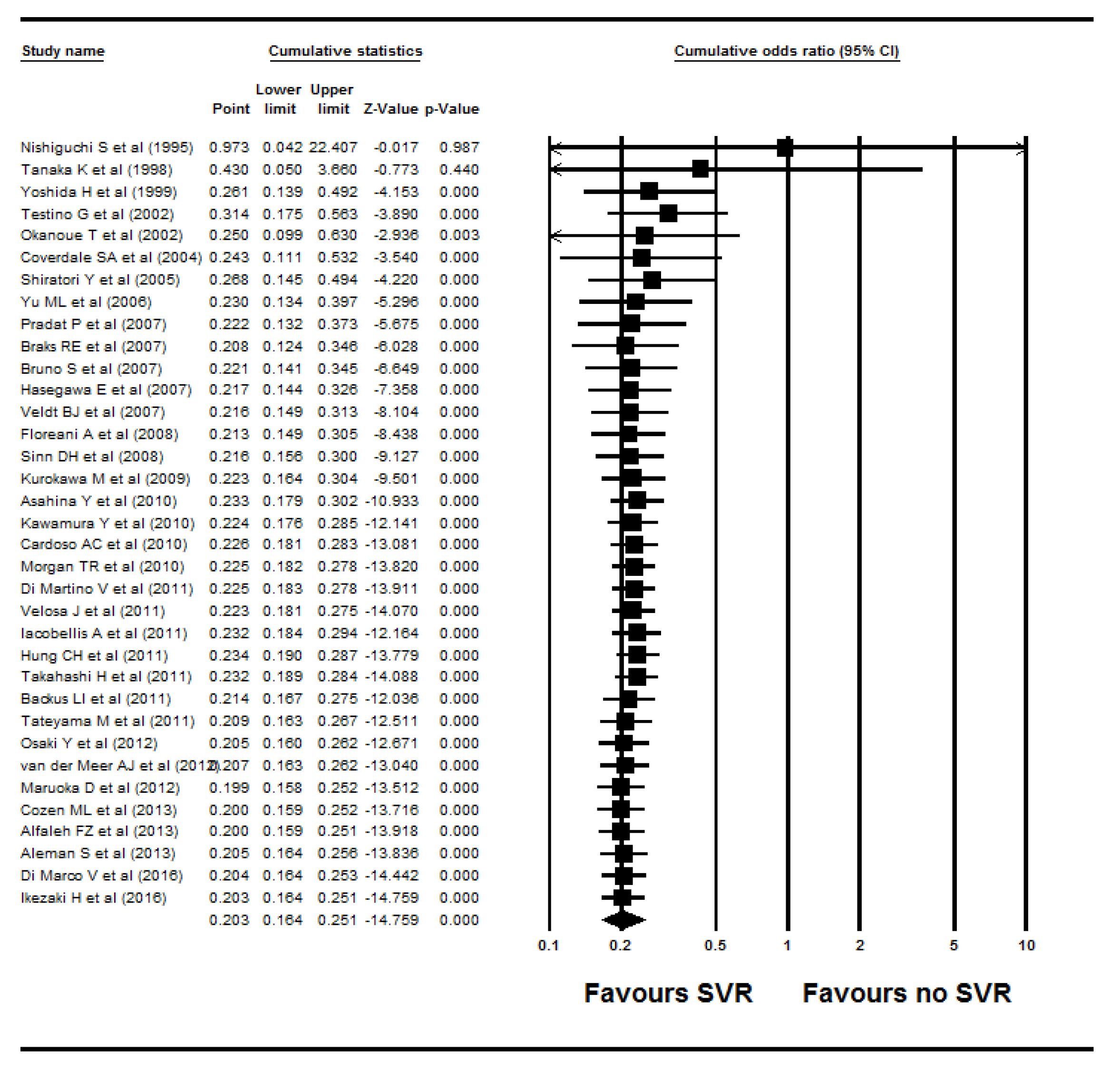
**

Diamond is the summary estimate from the pooled studies with 95% CI (Random effect model). SVR, sustained virologic response; HCC, hepatocellular carcinoma; CI, confidence interval.

**Appendix 20.** Cumulative meta-analysis of enrolled studies for the efficacy of SVR on the development of HCC (based on effect size).


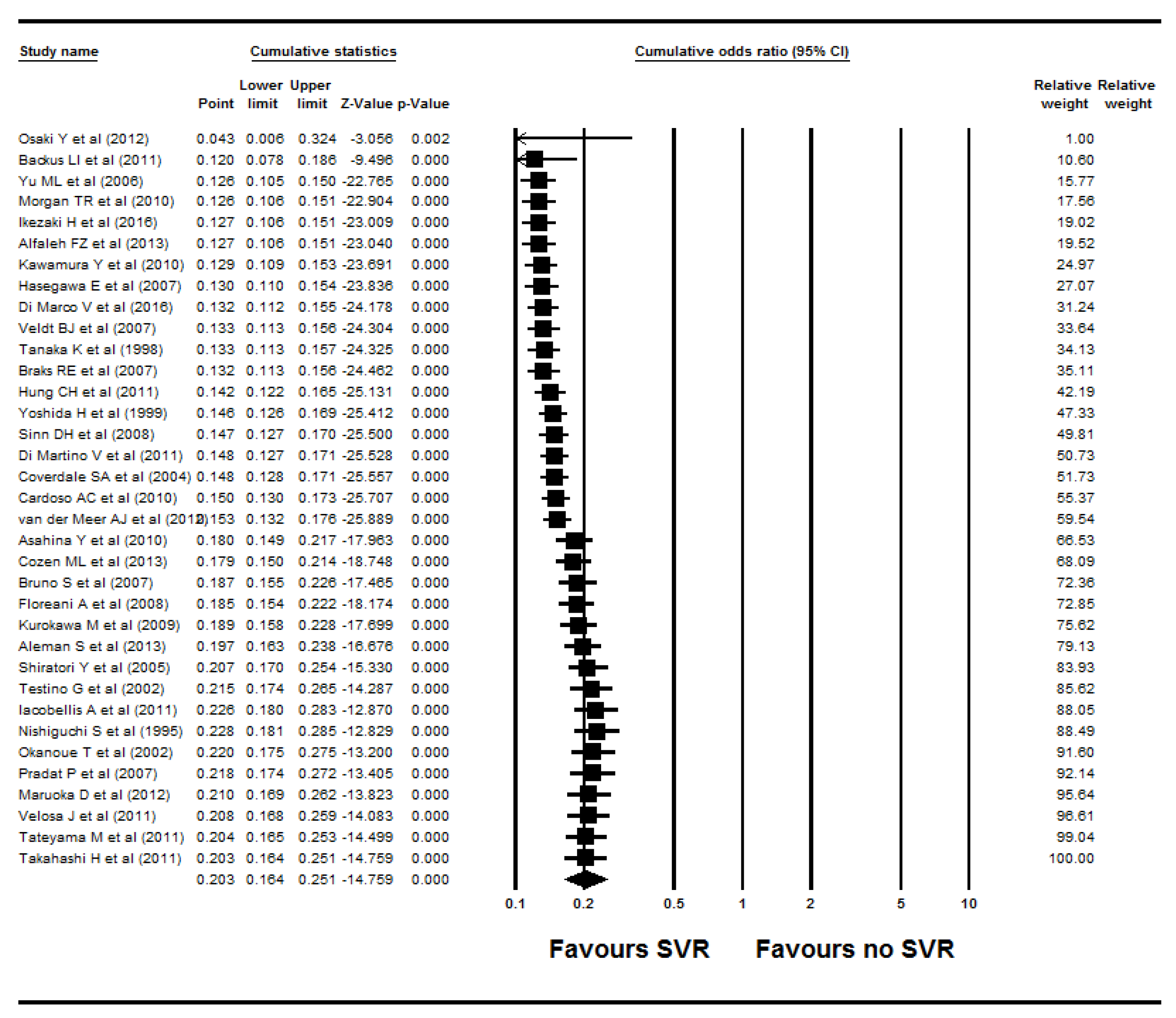


Diamond is the summary estimate from the pooled studies with 95% CI (Random effect model). SVR, sustained virologic response; HCC, hepatocellular carcinoma; CI, confidence interval.

**Appendix 21.** One study removed meta-analysis of enrolled studies for the efficacy of SVR on the development of HCC.

**
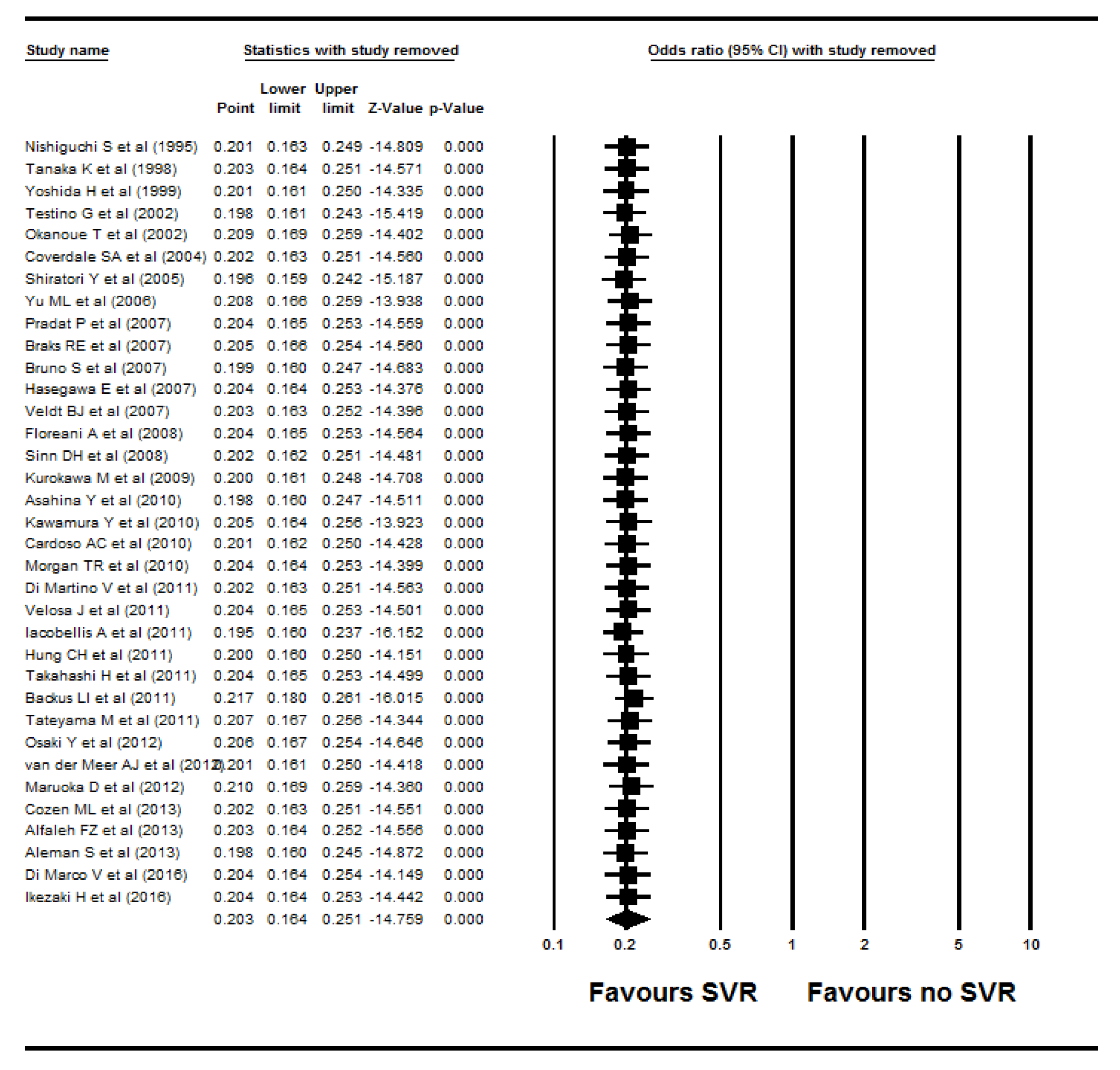
**

Diamond is the summary estimate from the pooled studies with 95% CI (Random effect model). SVR, sustained virologic response; HCC, hepatocellular carcinoma; CI, confidence interval.

**Appendix 22.** Meta-ANOVA according to the modifiers for the efficacy of SVR on the development of HCC (study format / Nationality / Histology / Follow-up duration / Newcastle-Ottawa scale / Age / Treatment).

**
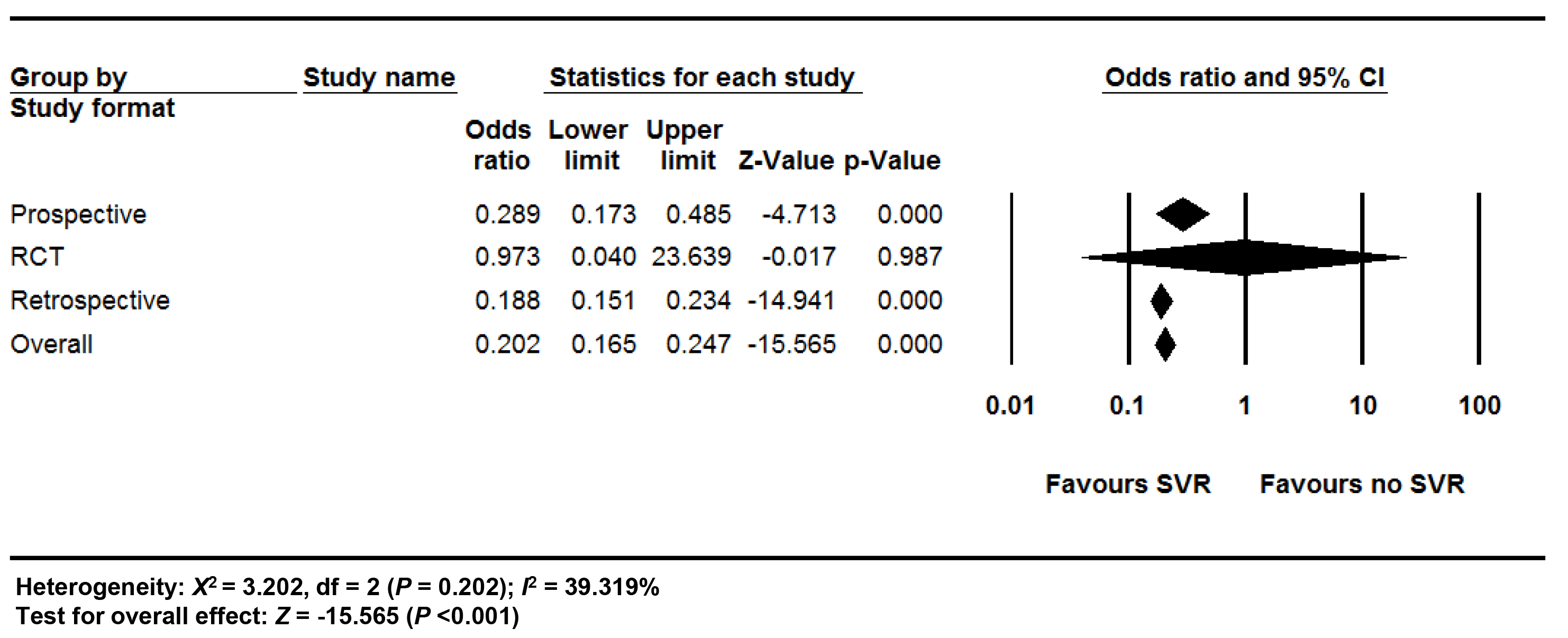
**

**
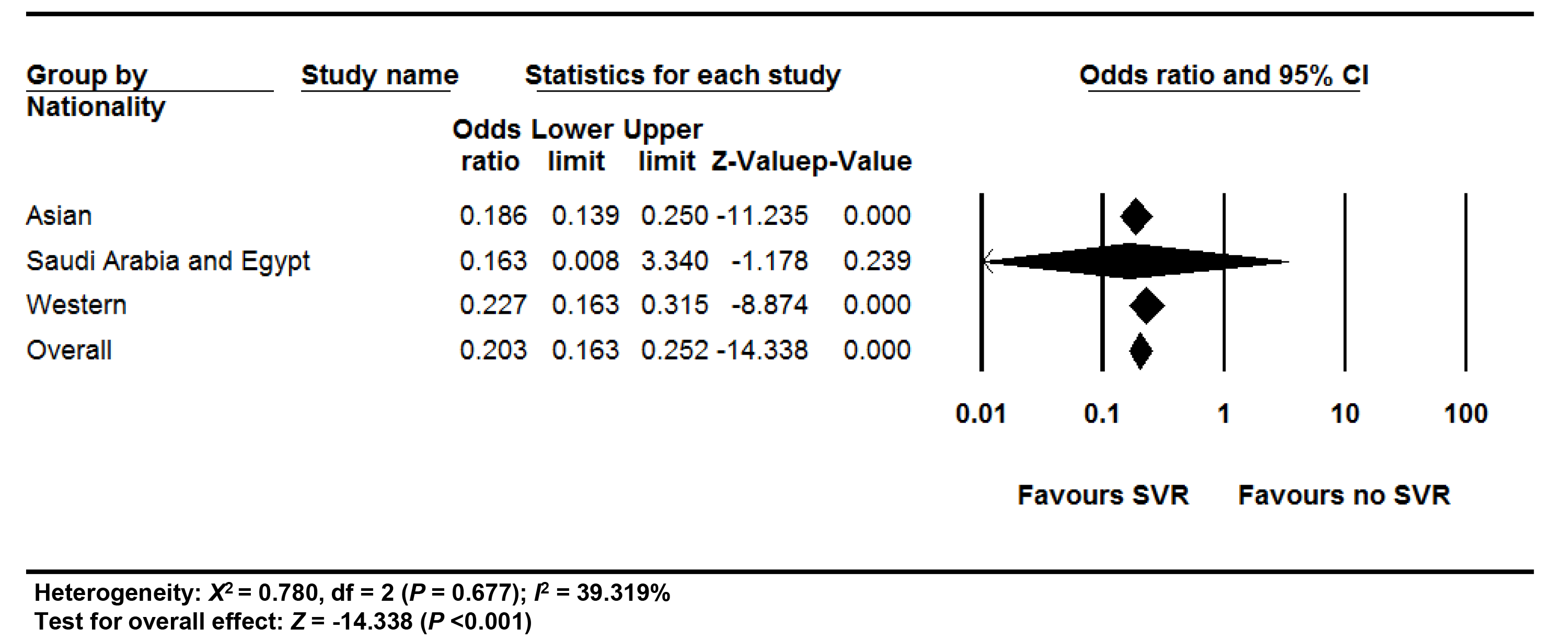
**

**
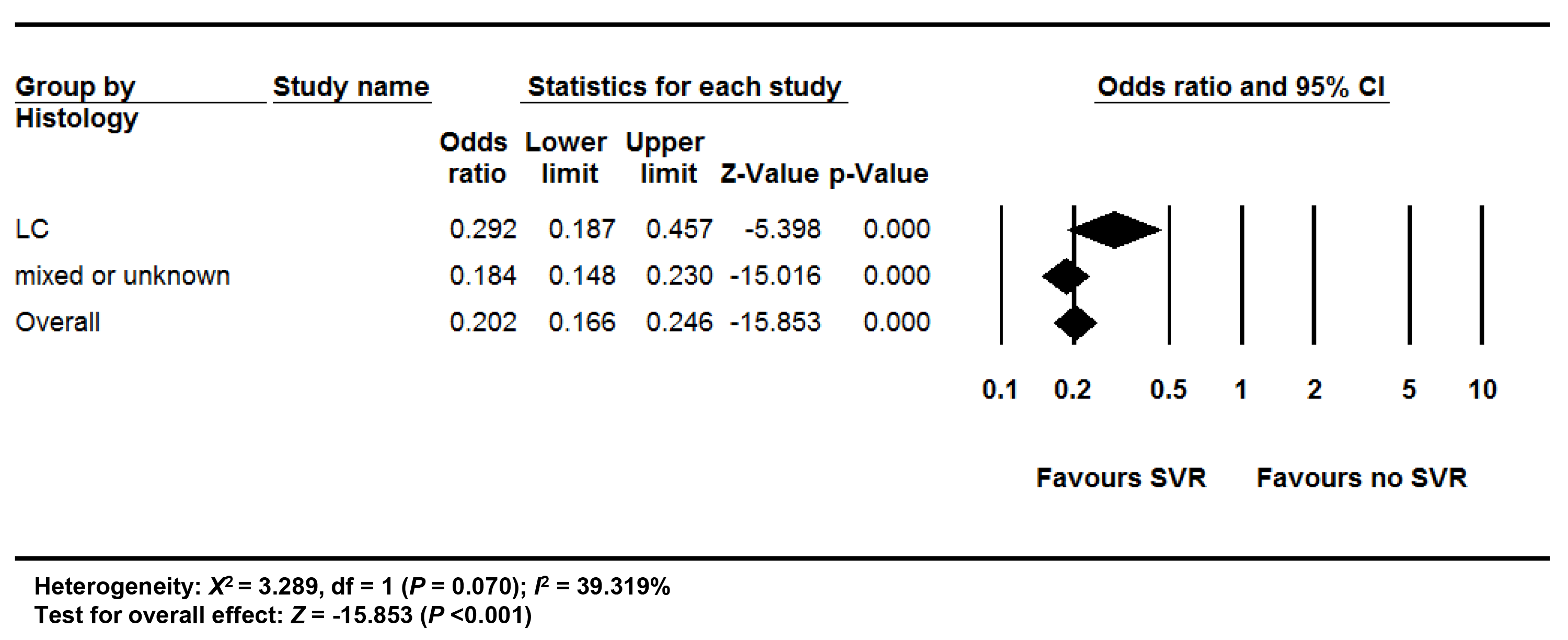
**

**
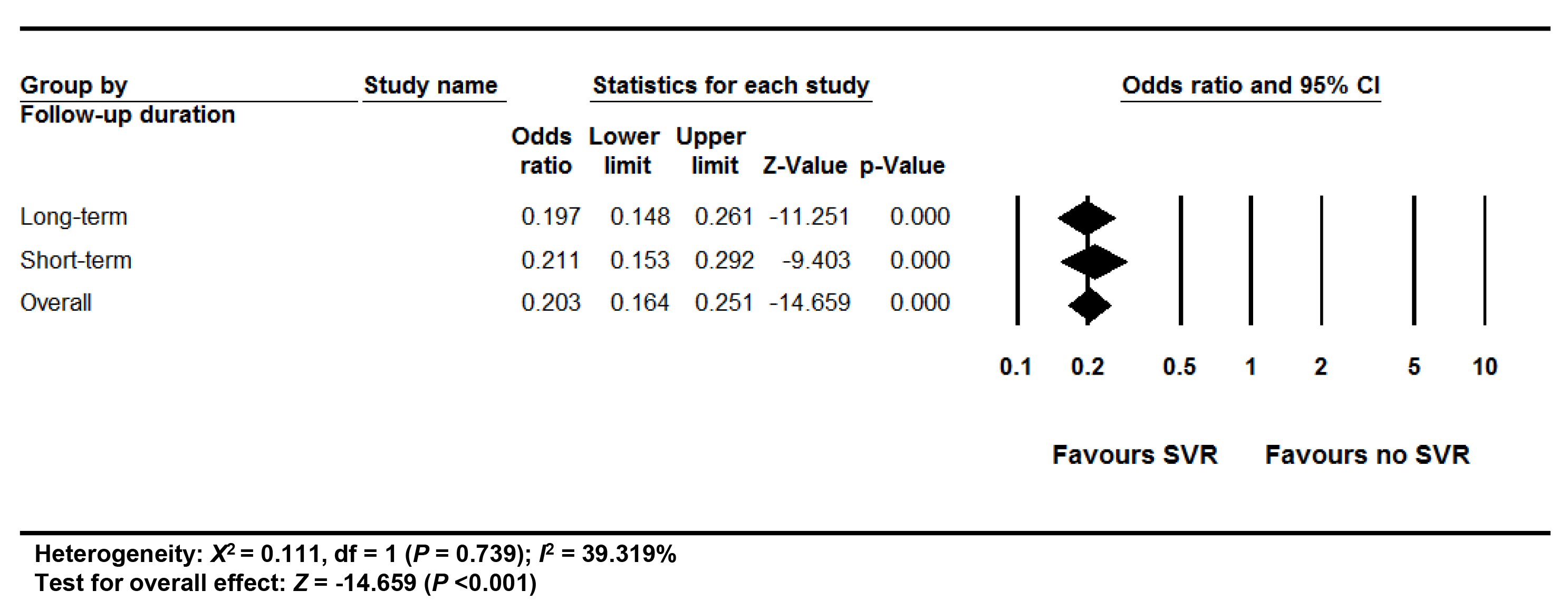
**

**
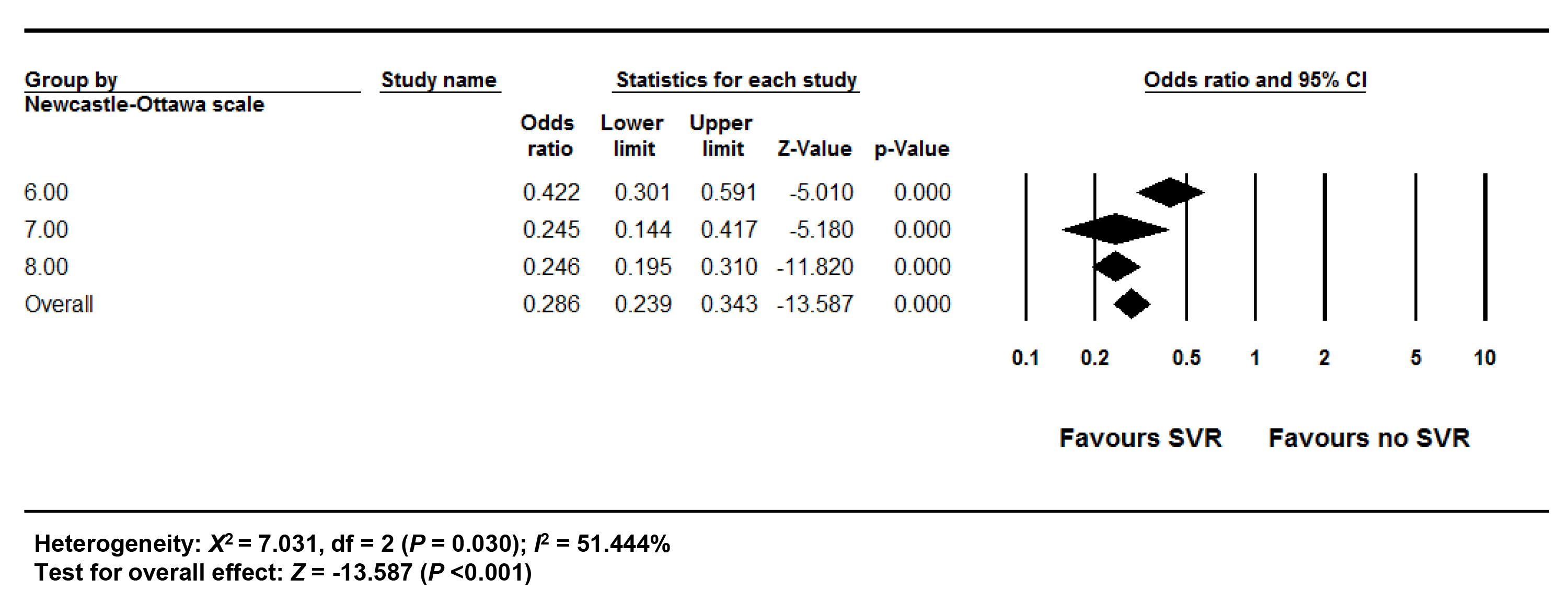

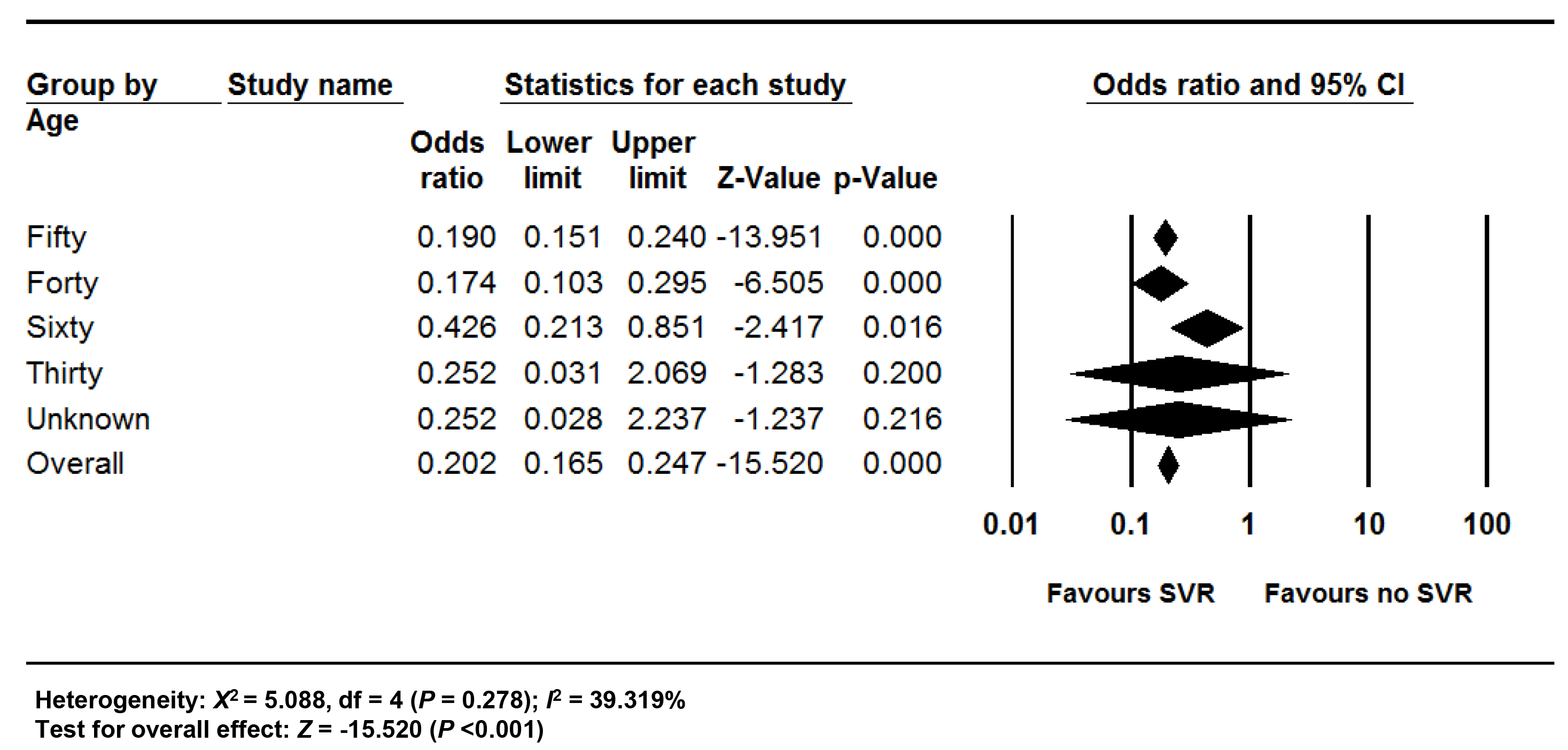
**

**
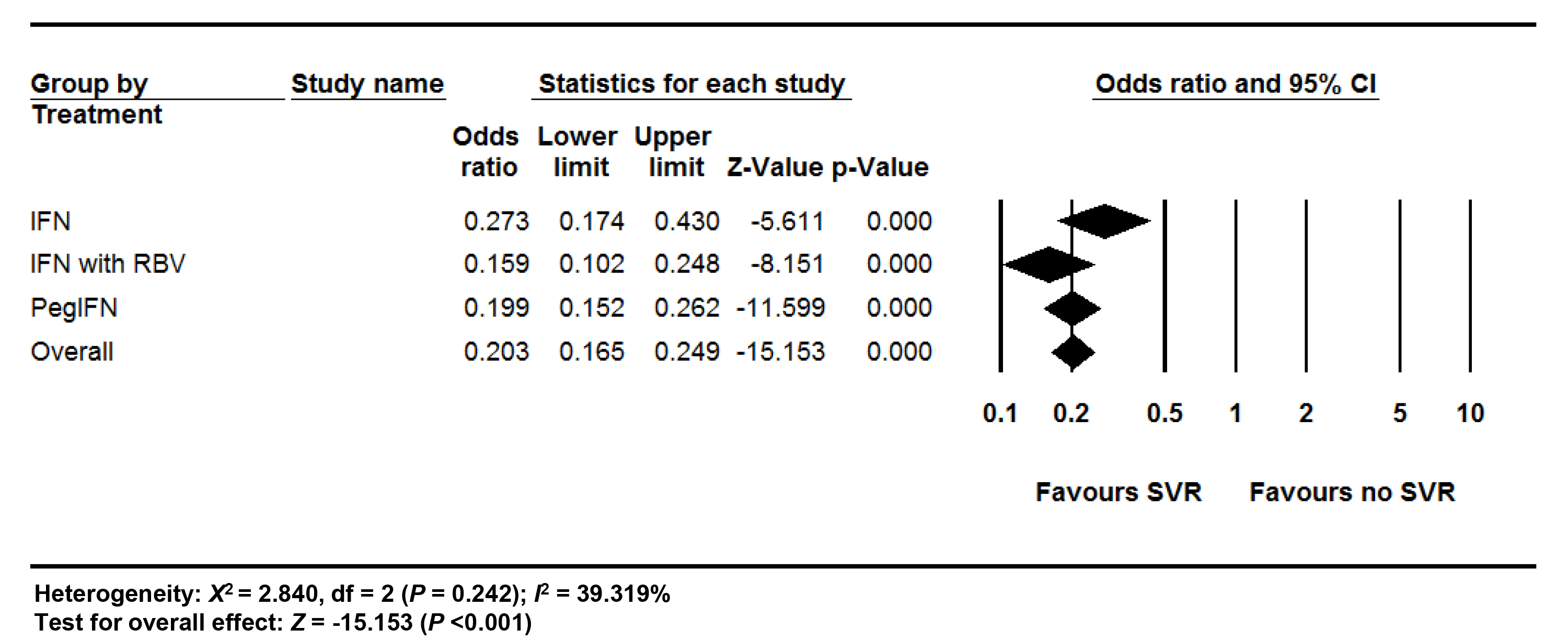
**

Diamond is the summary estimate from the pooled studies with 95% CI (Mixed effect model). SVR, sustained virologic response; HCC, hepatocellular carcinoma; CI, confidence interval.

**Appendix 23.** Funnel plot of studies for efficacy of SVR on all-cause mortality.

**
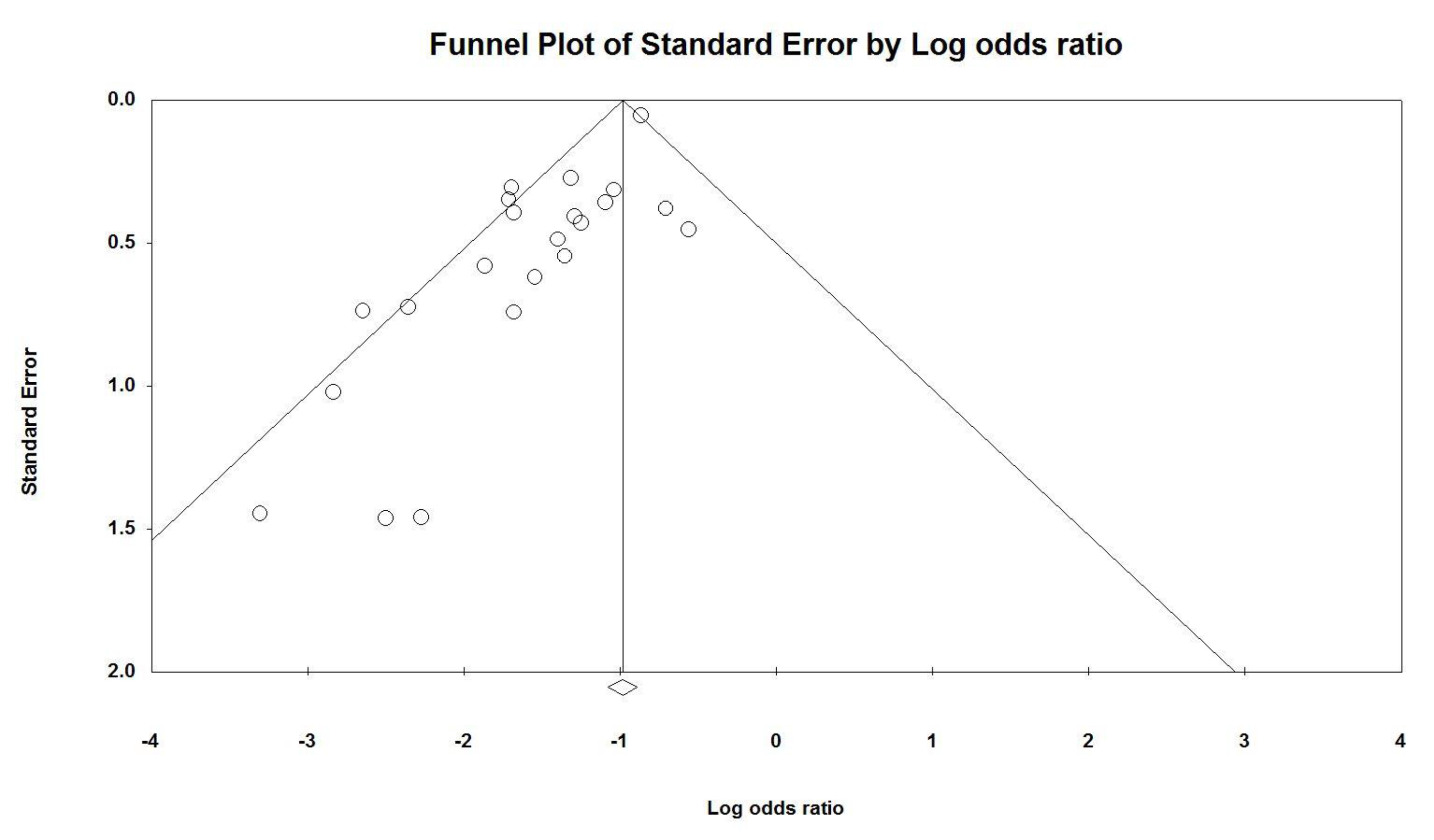
**

The line in center is the natural logarithm of pooled OR, and 2 oblique lines are pseudo 95% confidence limits. OR, odds ratio.

**Appendix 24.** Cumulative meta-analysis of enrolled studies for the efficacy of SVR on all-cause mortality (based on publication year).

**
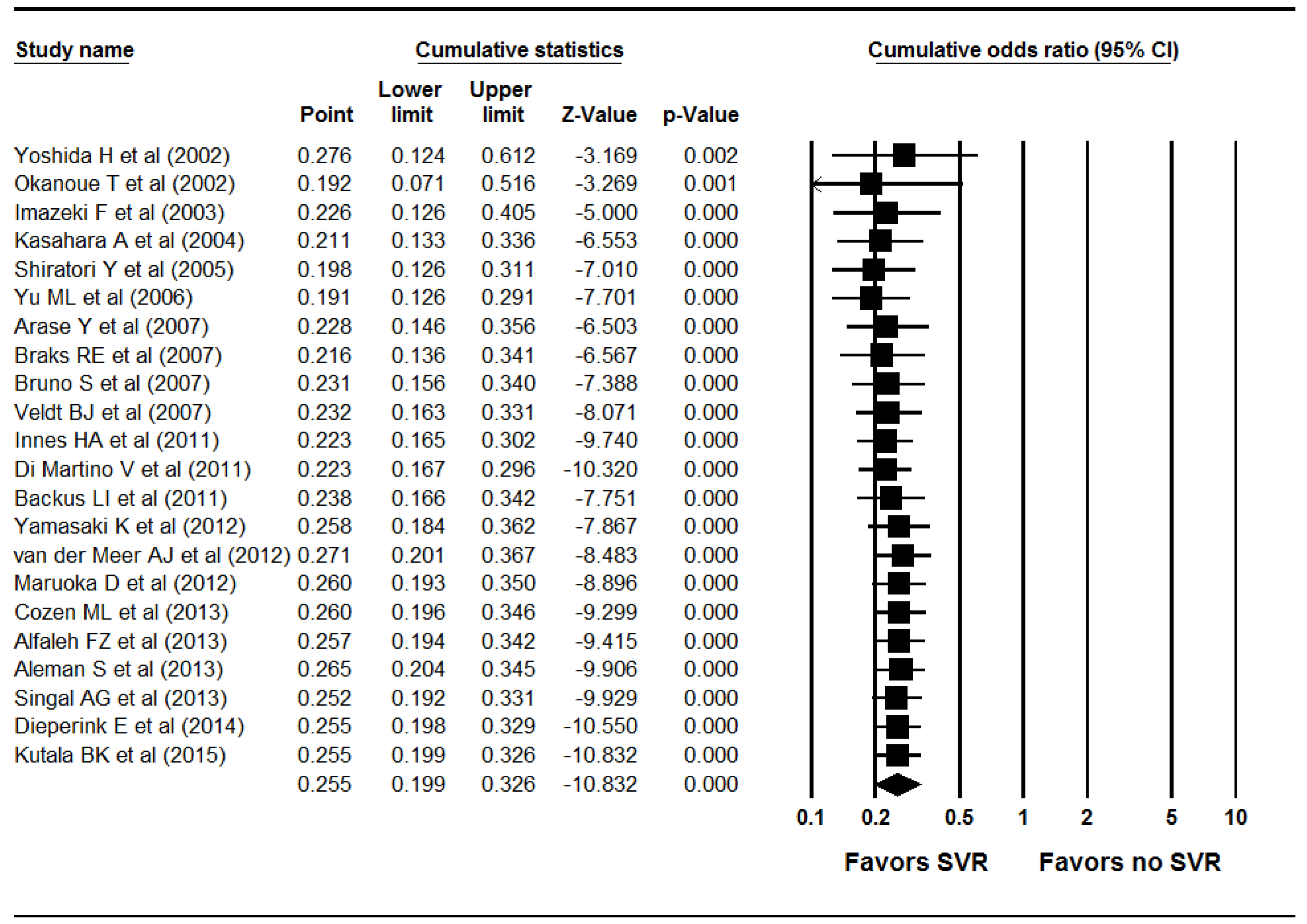
**

Diamond is the summary estimate from the pooled studies with 95% CI (Random effect model). SVR, sustained virologic response; CI, confidence interval.

**Appendix 25.** Cumulative meta-analysis of enrolled studies for the efficacy of SVR on all-cause mortality (based on effect size).

**
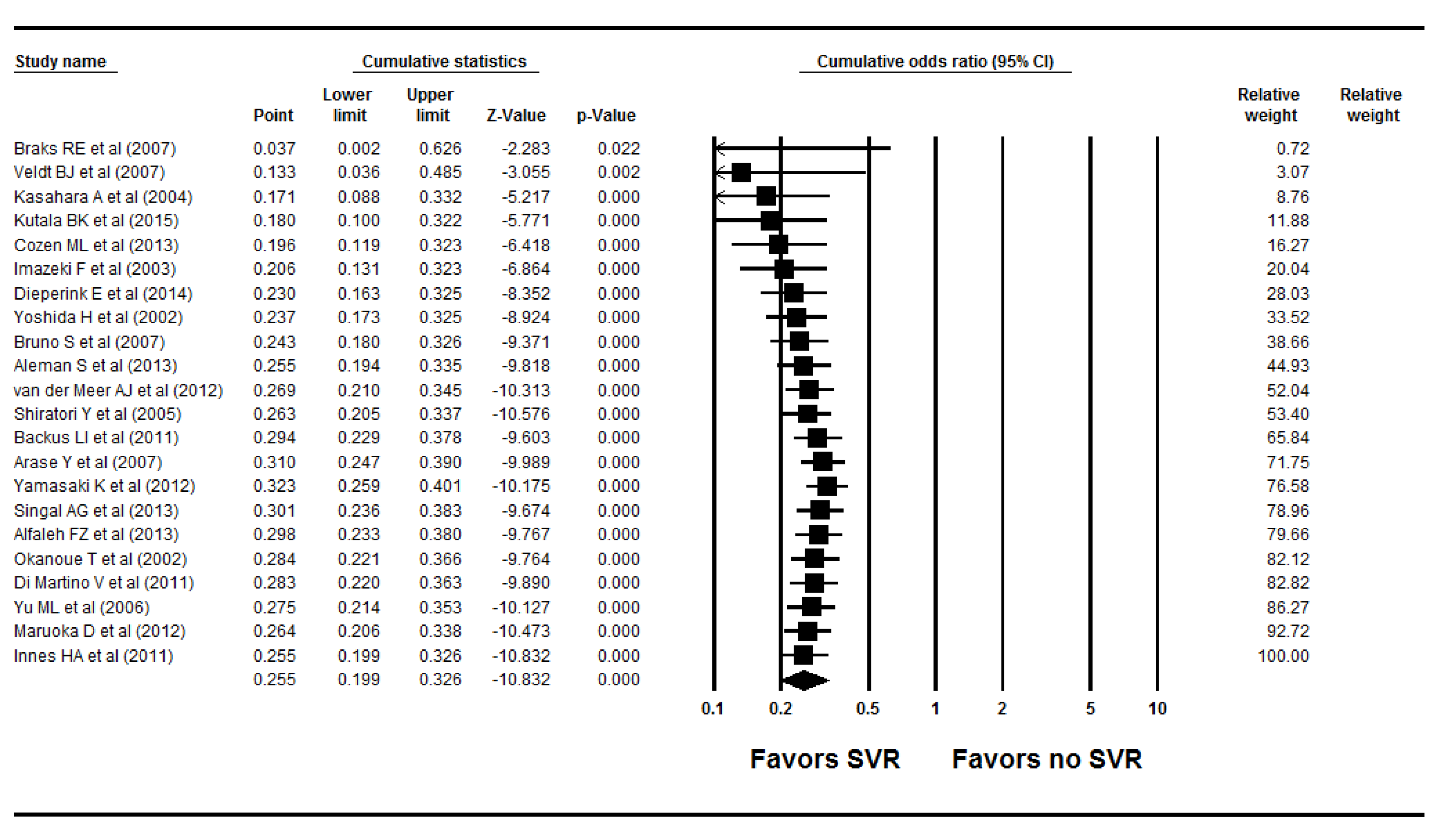
**

Diamond is the summary estimate from the pooled studies with 95% CI (Random effect model). SVR, sustained virologic response; CI, confidence interval.

**Appendix 26.** One study removed meta-analysis of enrolled studies for the efficacy of SVR on all-cause mortality.

**
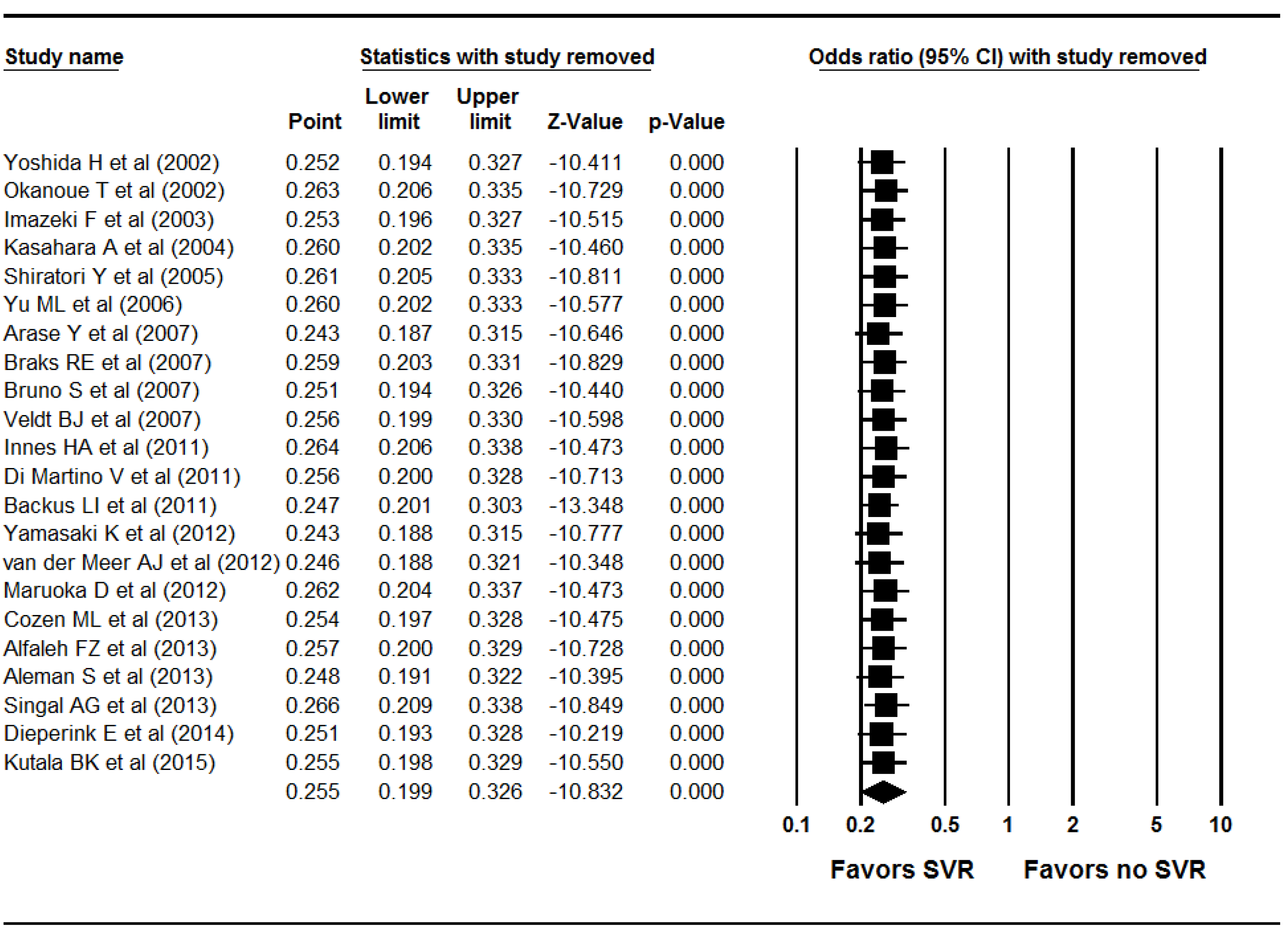
**Diamond is the summary estimate from the pooled studies with 95% CI (Random effect model). SVR, sustained virologic response; CI, confidence interval.

**Appendix 27.** Meta-ANOVA according to the modifiers for the efficacy of SVR on all-cause mortality (study format / Nationality / Histology / Follow-up duration / Newcastle-Ottawa scale / Age / Treatment).

**
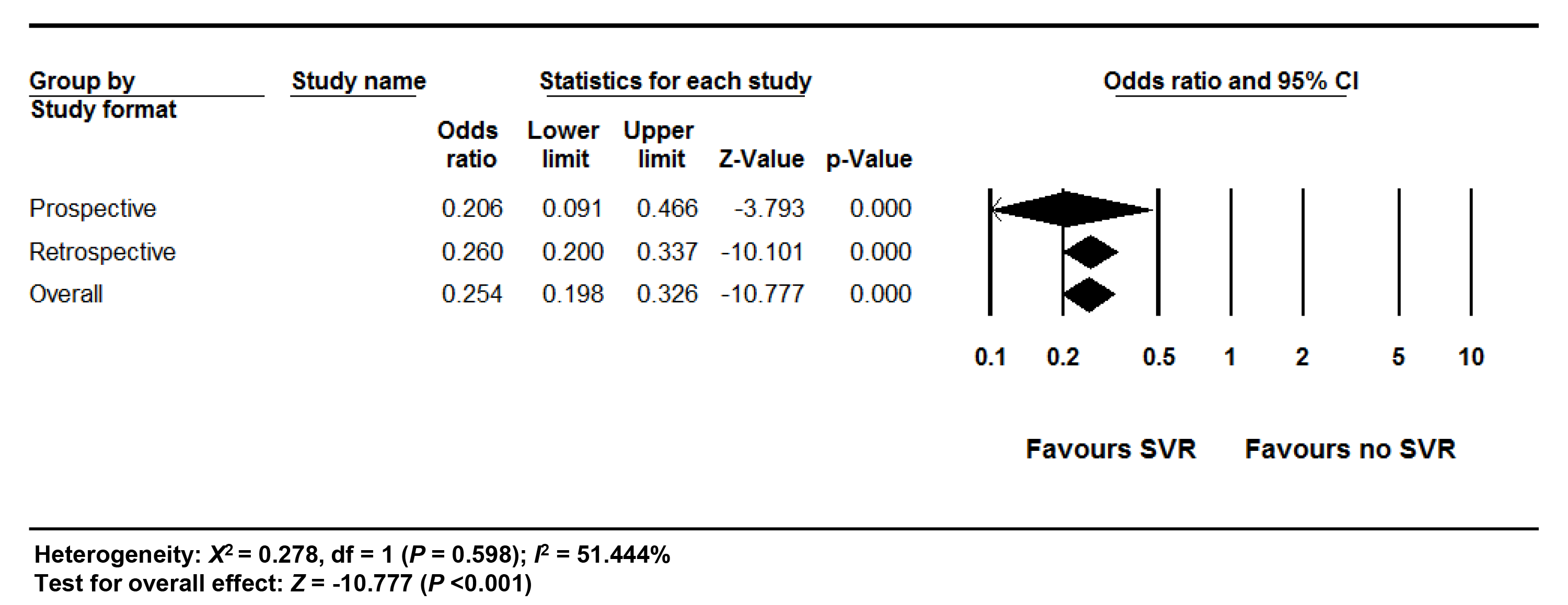
**

**
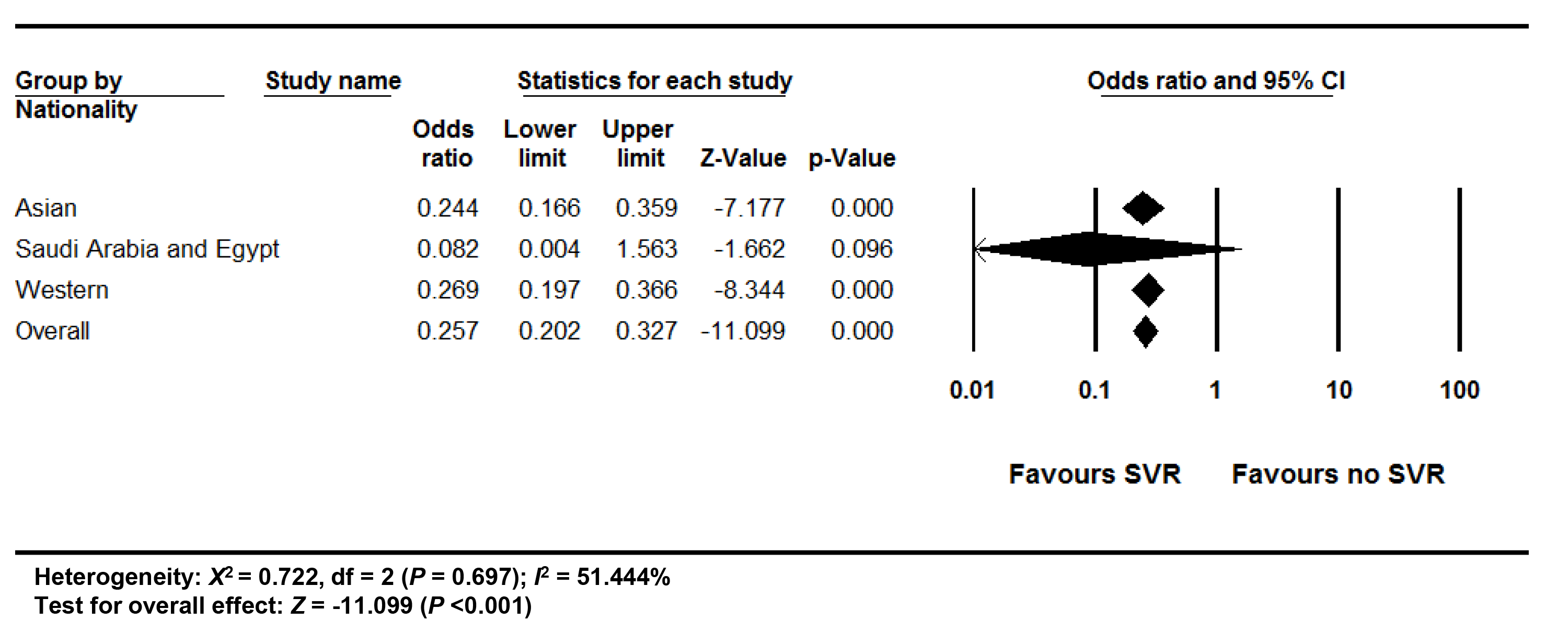
**

**
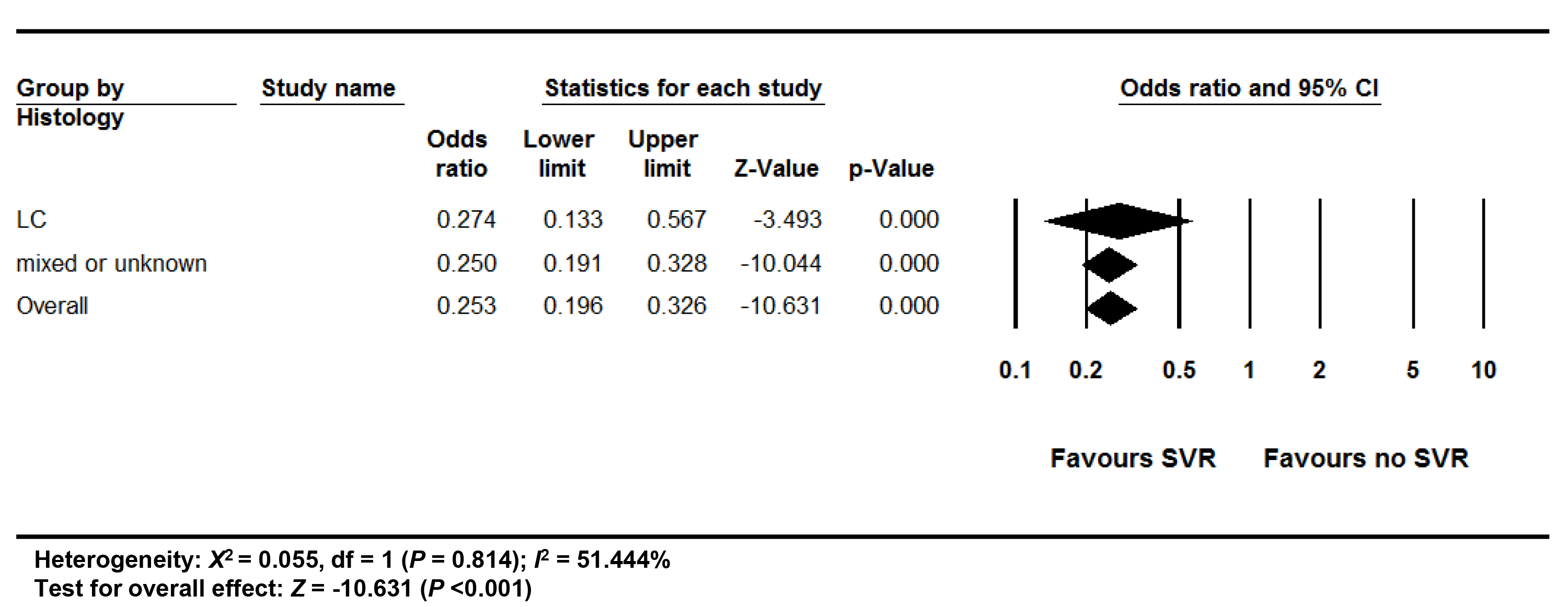
**

**
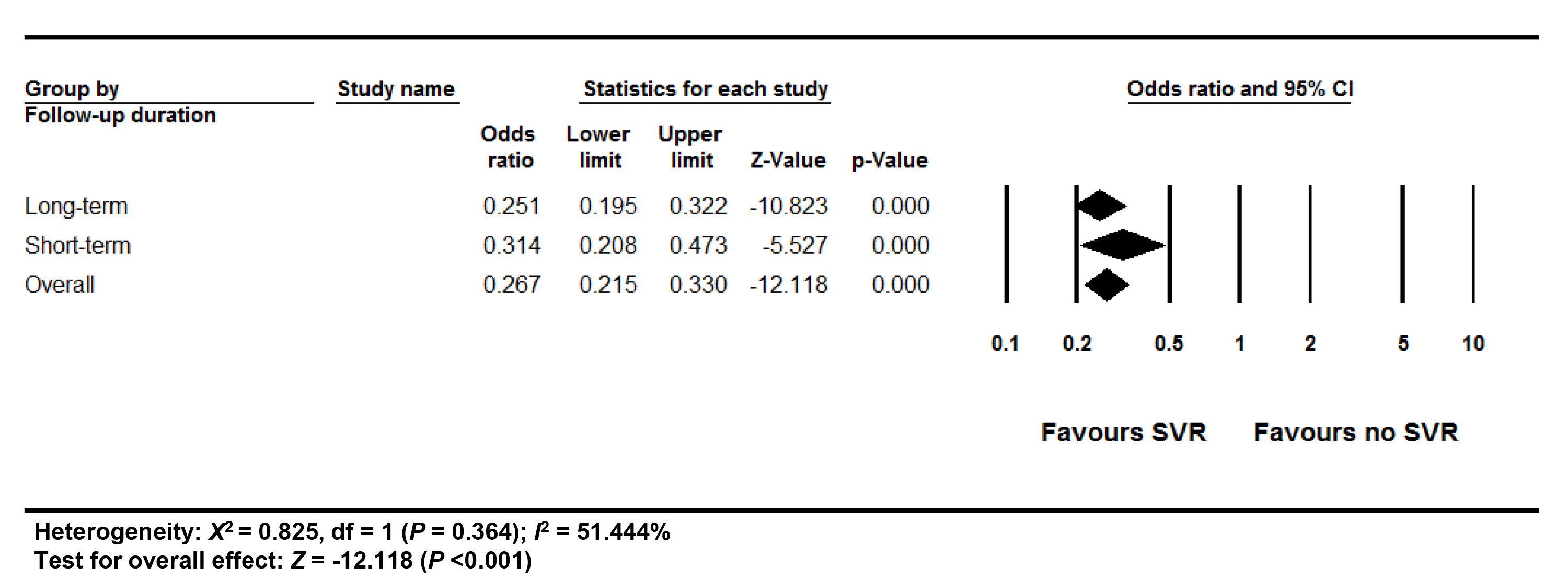
**

**
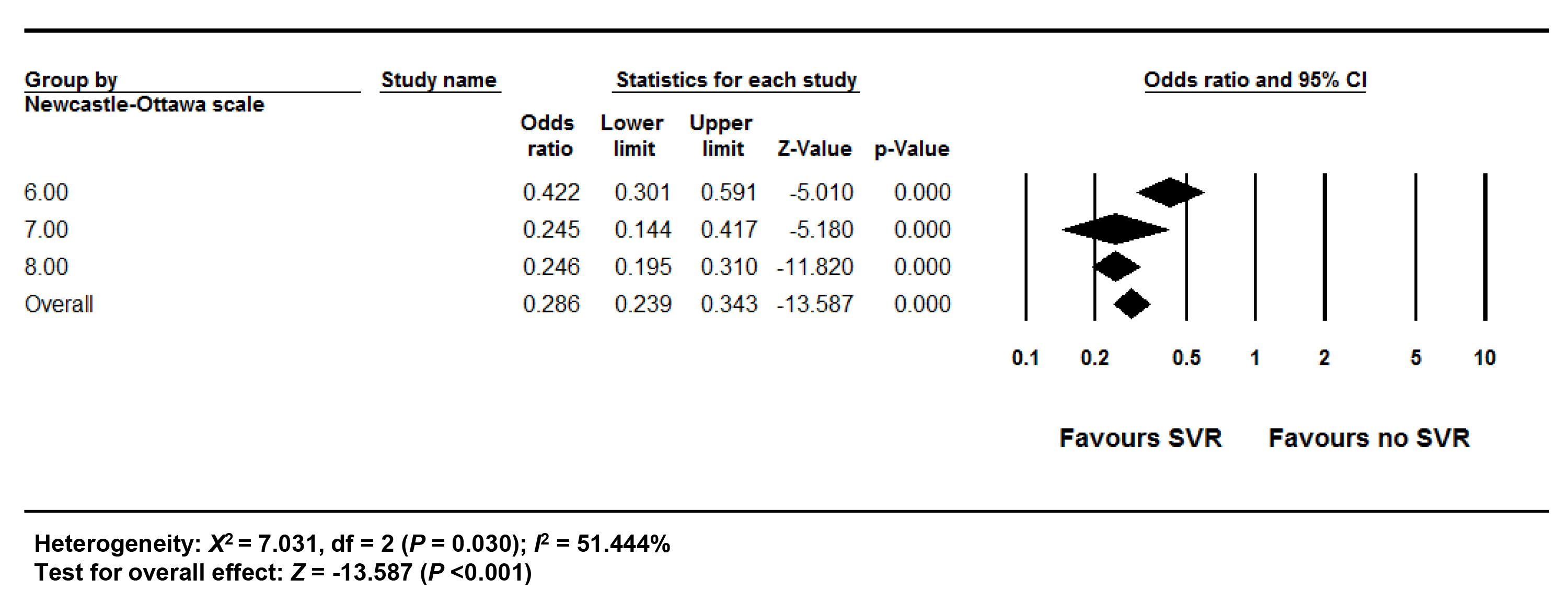
**

**
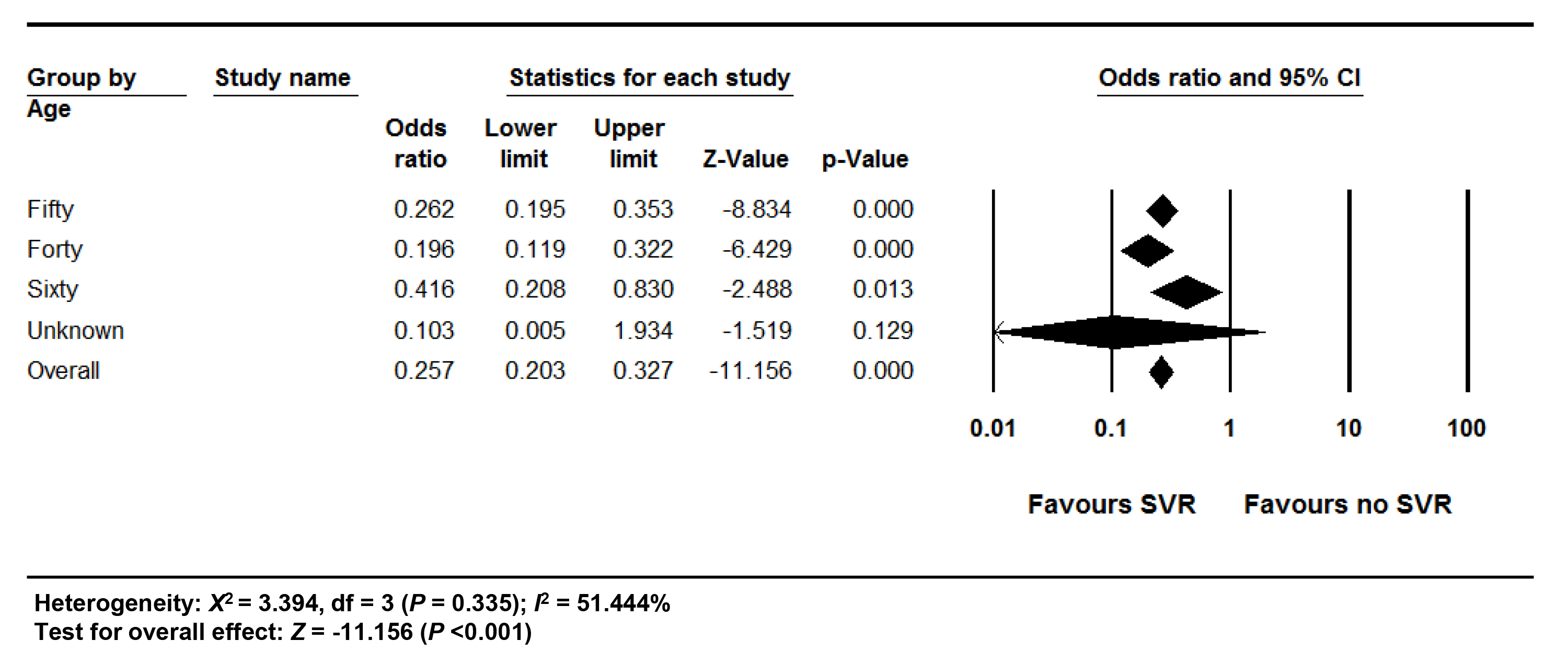
**

**
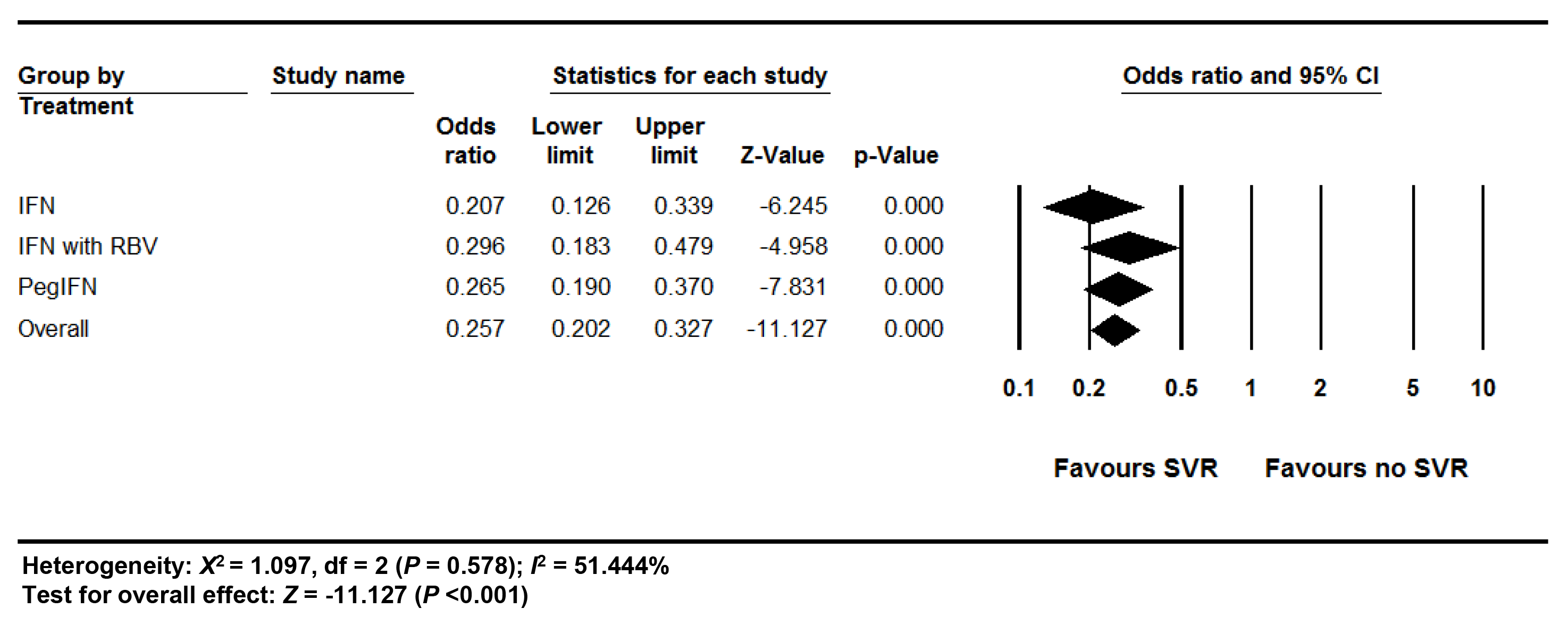
**

Diamond is the summary estimate from the pooled studies with 95% CI (Mixed effect model). SVR, sustained virologic response; CI, confidence interval.

**Appendix 28.** Efficacy of SVR on liver-specific mortality in patients with chronic hepatitis C.


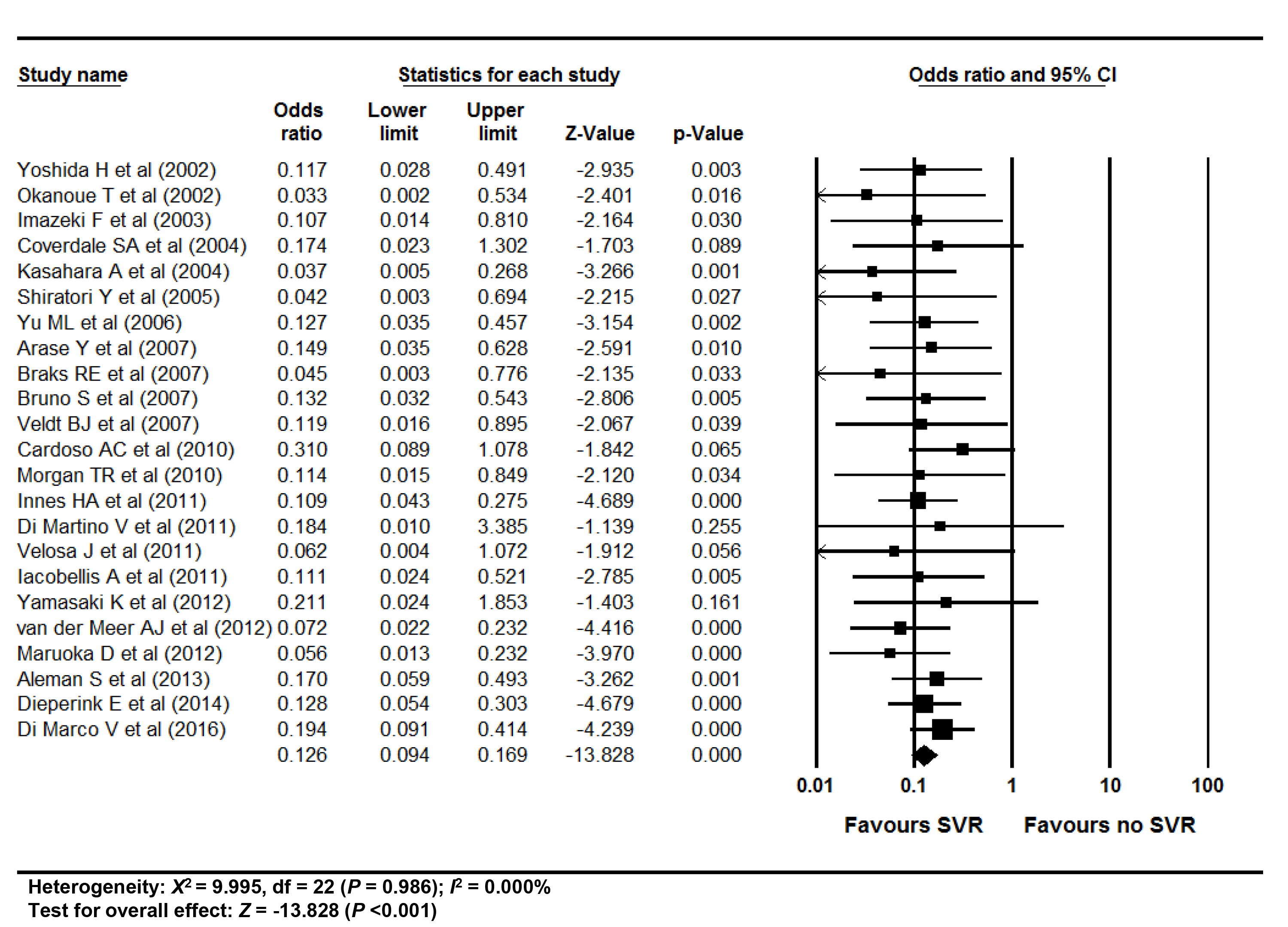


The size of each square is proportional to the study’s weight. Diamond is the summary estimate from the pooled studies (random effect model). SVR, sustained virologic response.

**Appendix 29.** Funnel plot of studies for efficacy of SVR on liver-specific mortality.

**
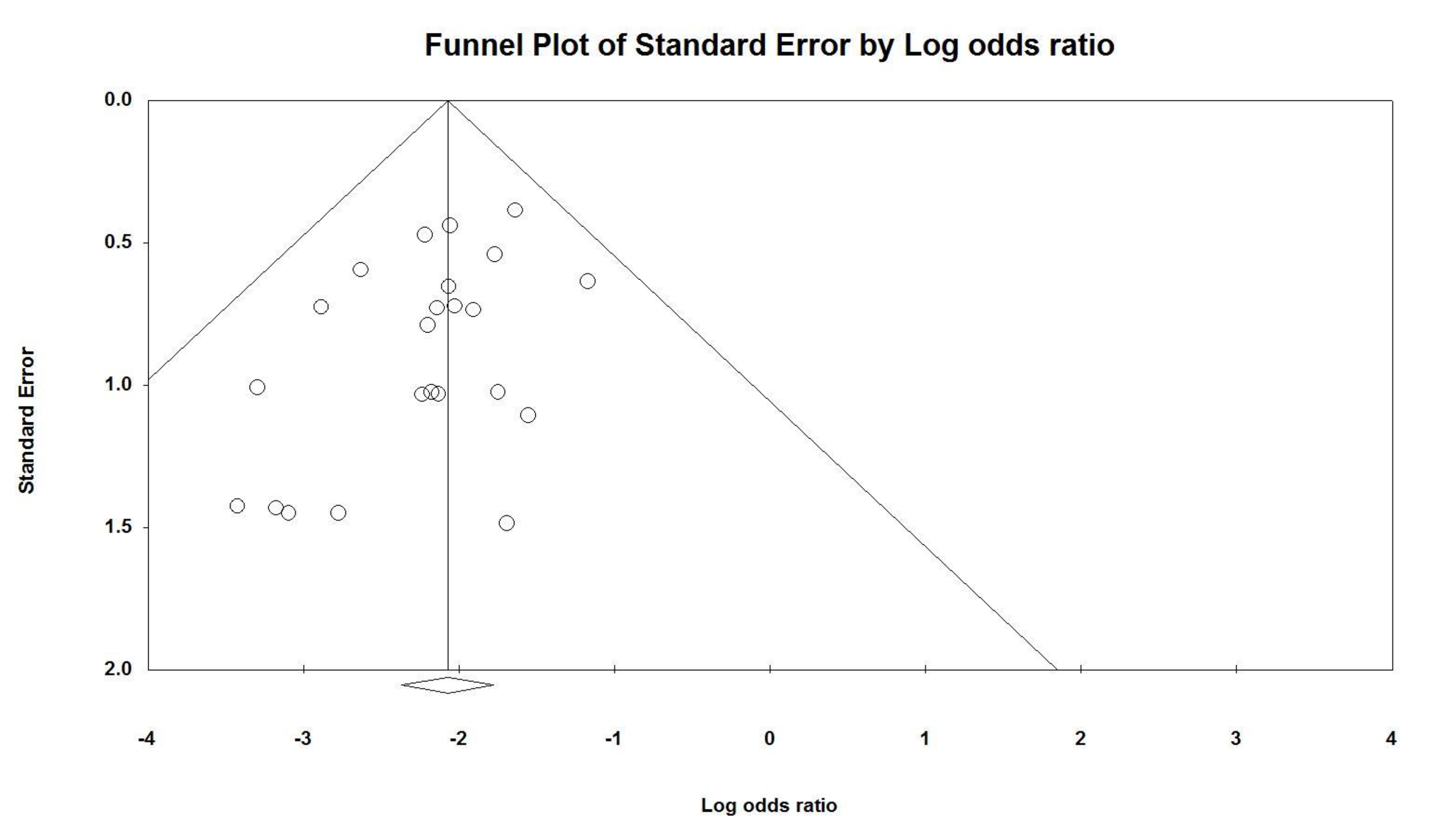
**

The line in center is the natural logarithm of pooled OR, and 2 oblique lines are pseudo 95% confidence limits. OR, odds ratio.

**Appendix 30.** Cumulative meta-analysis of enrolled studies for the efficacy of SVR on liver-specific mortality (based on publication year).

**
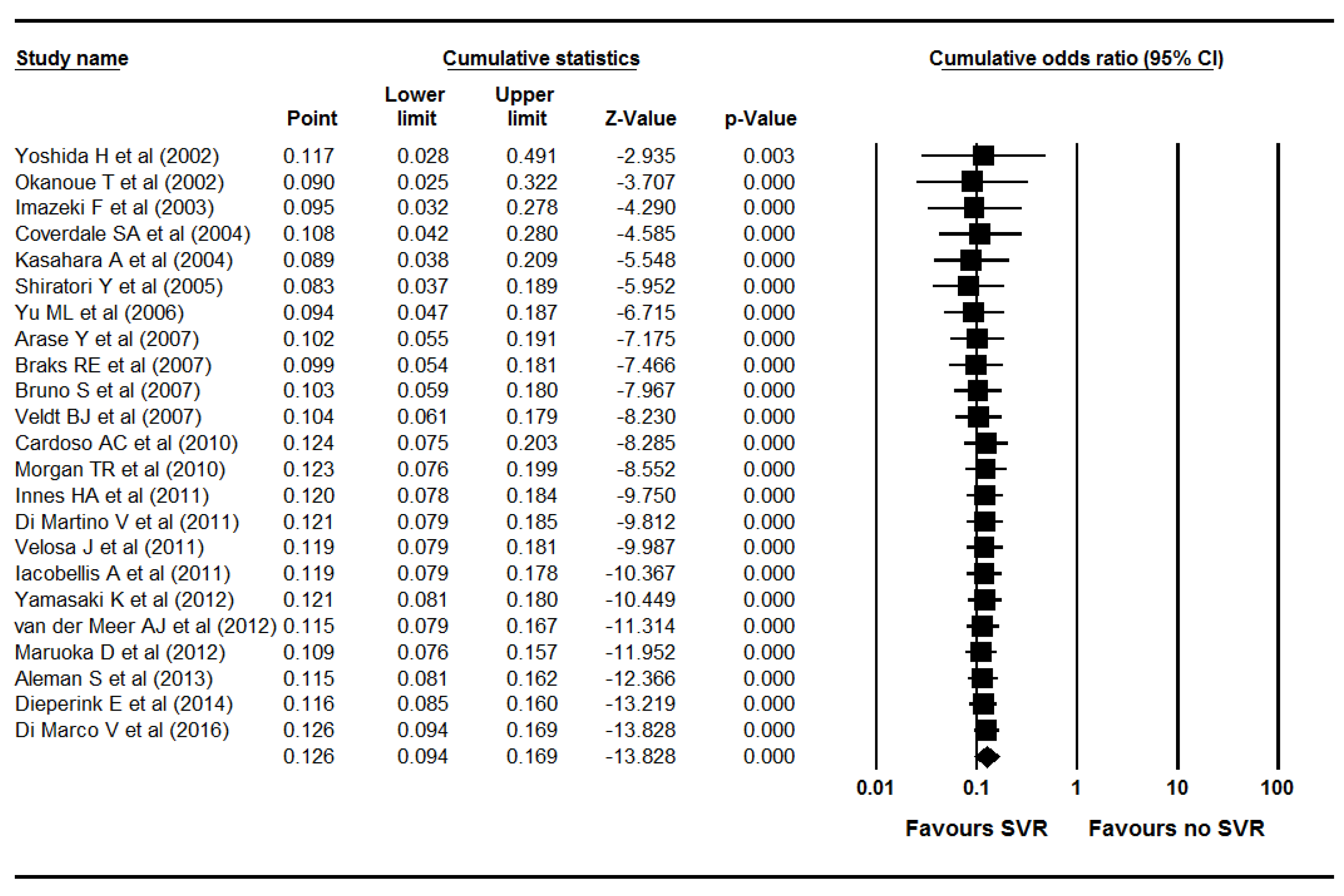
**

Diamond is the summary estimate from the pooled studies with 95% CI (Random effect model). SVR, sustained virologic response; CI, confidence interval.

**Appendix 31.** Cumulative meta-analysis of enrolled studies for the efficacy of SVR on liver-specific mortality (based on effect size).

**
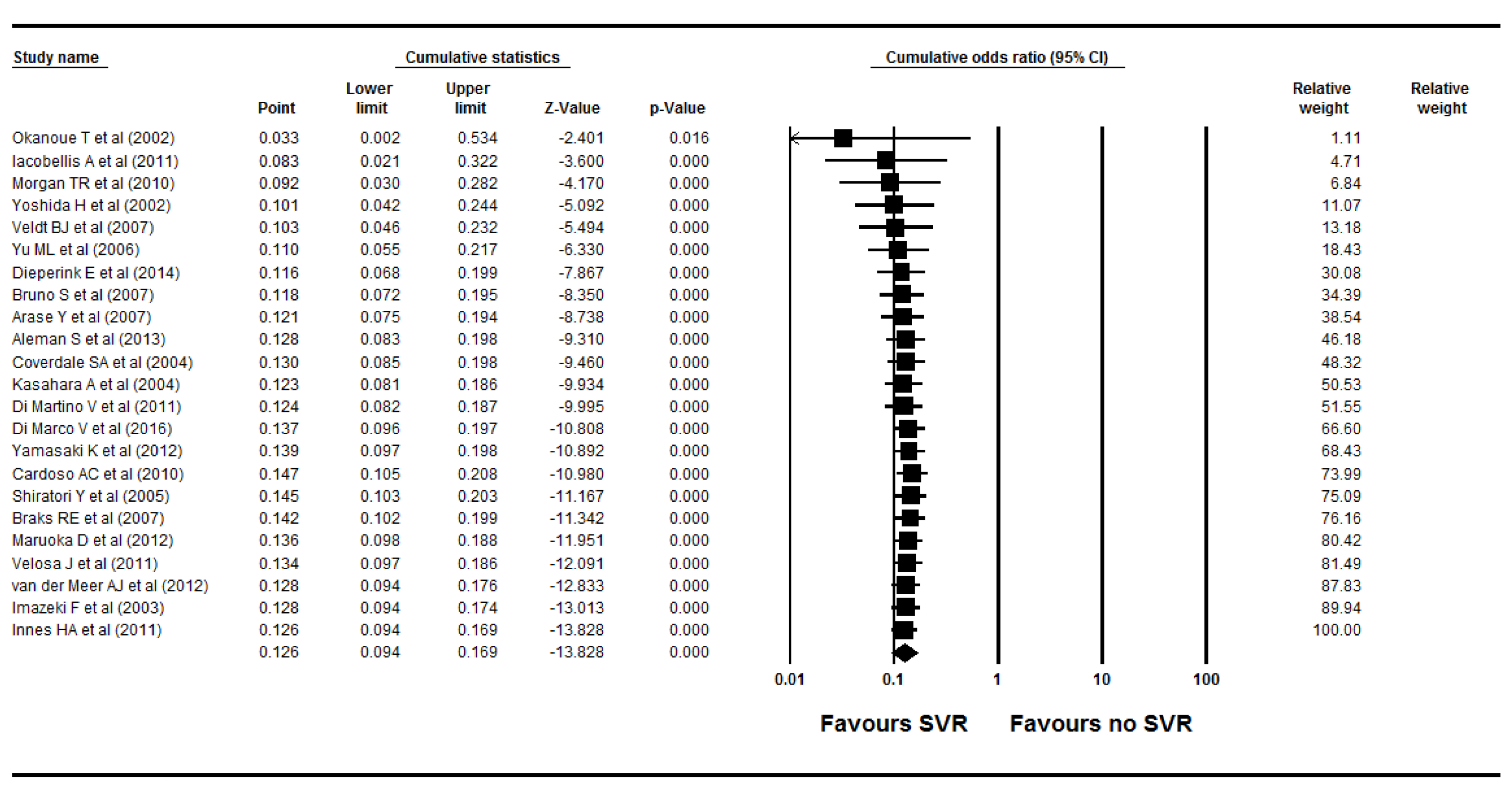
**

Diamond is the summary estimate from the pooled studies with 95% CI (Random effect model). SVR, sustained virologic response; CI, confidence interval.

**Appendix 32.** One study removed meta-analysis of enrolled studies for the efficacy of SVR on liver-specific mortality.

**
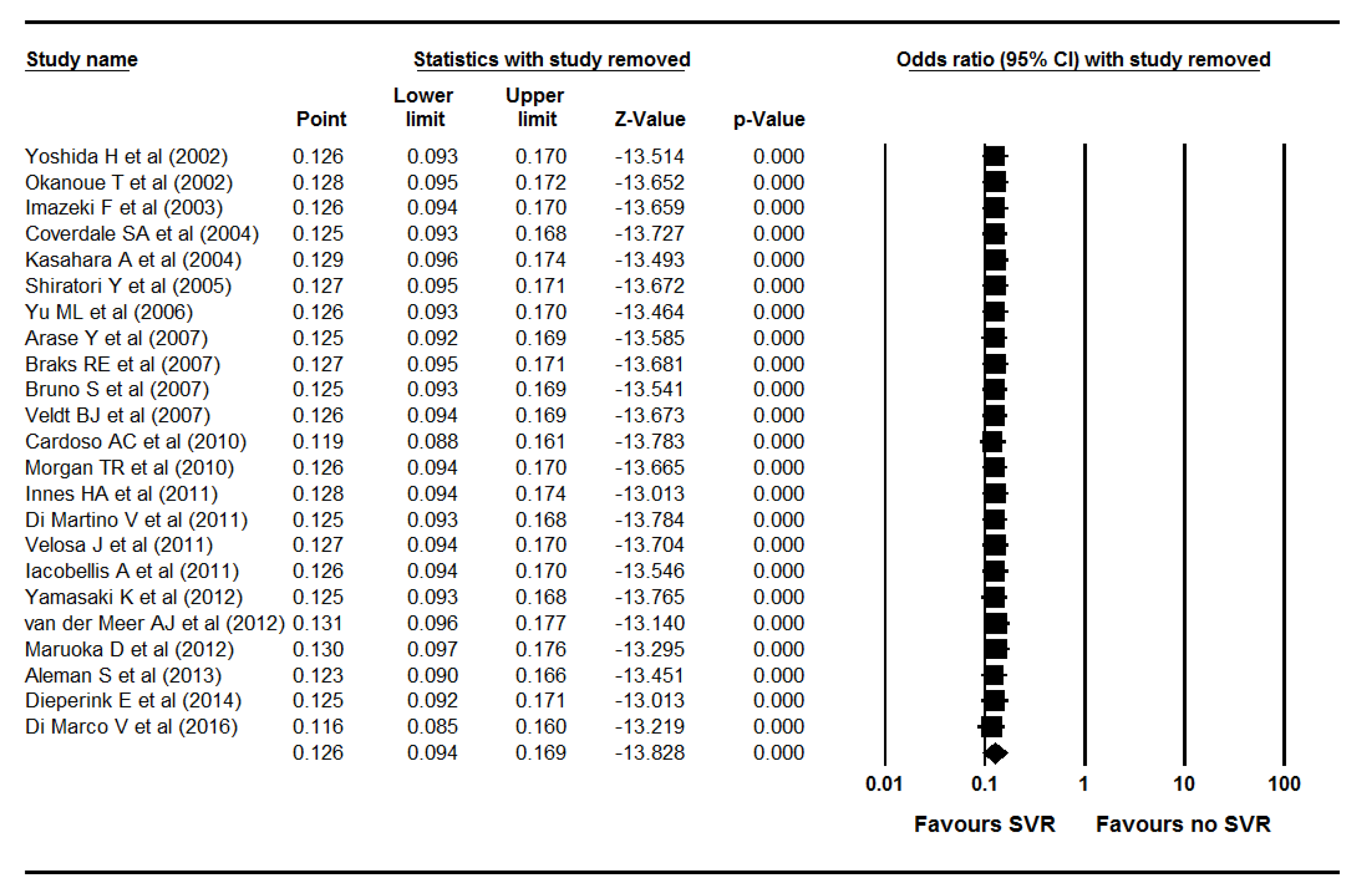
**Diamond is the summary estimate from the pooled studies with 95% CI (Random effect model). SVR, sustained virologic response; CI, confidence interval.

**Appendix 33.** Meta-ANOVA according to the modifiers for the efficacy of SVR on liver-specific mortality (study format / Nationality / Histology / Follow-up duration / Newcastle-Ottawa scale / Age / Treatment).

**
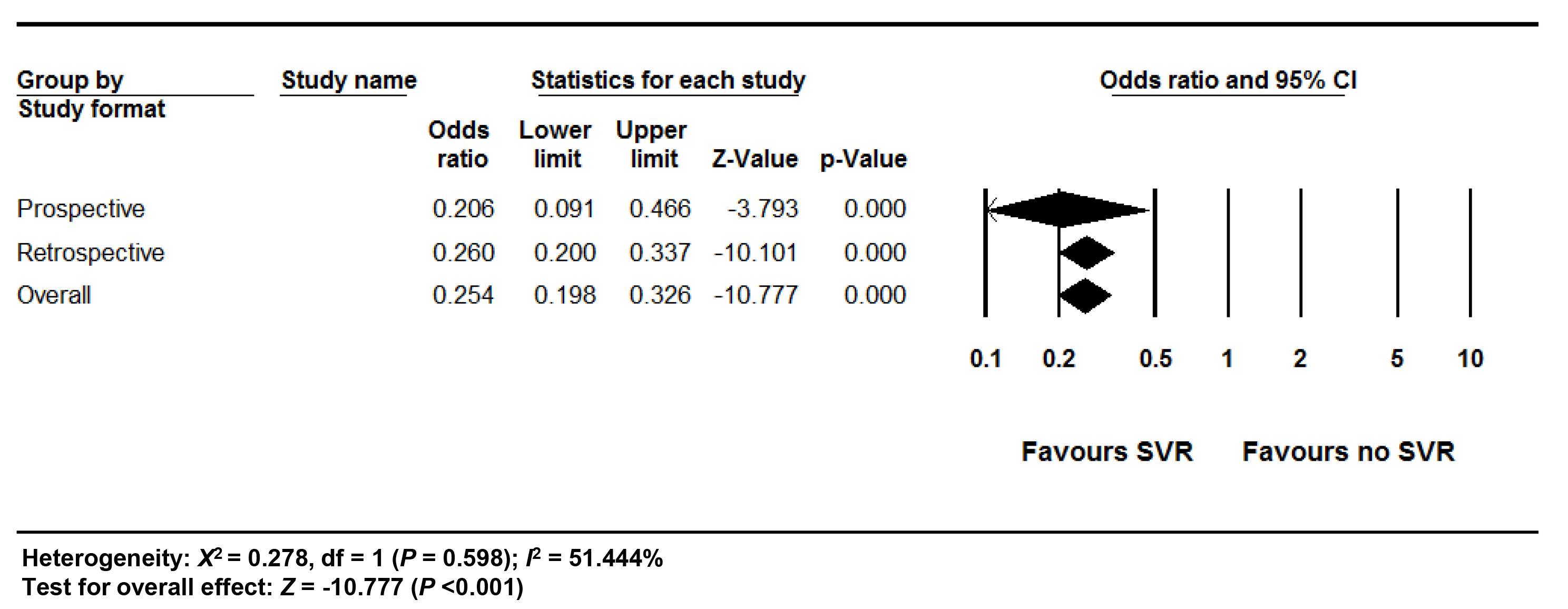
**

**
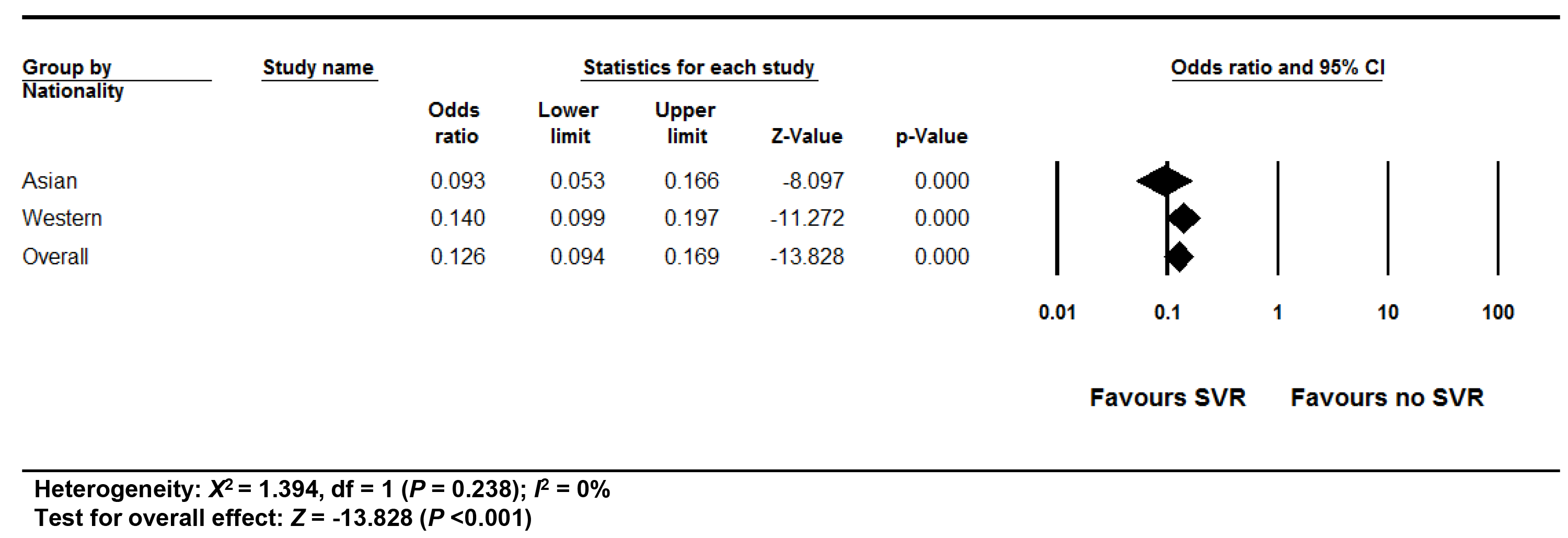
**

**
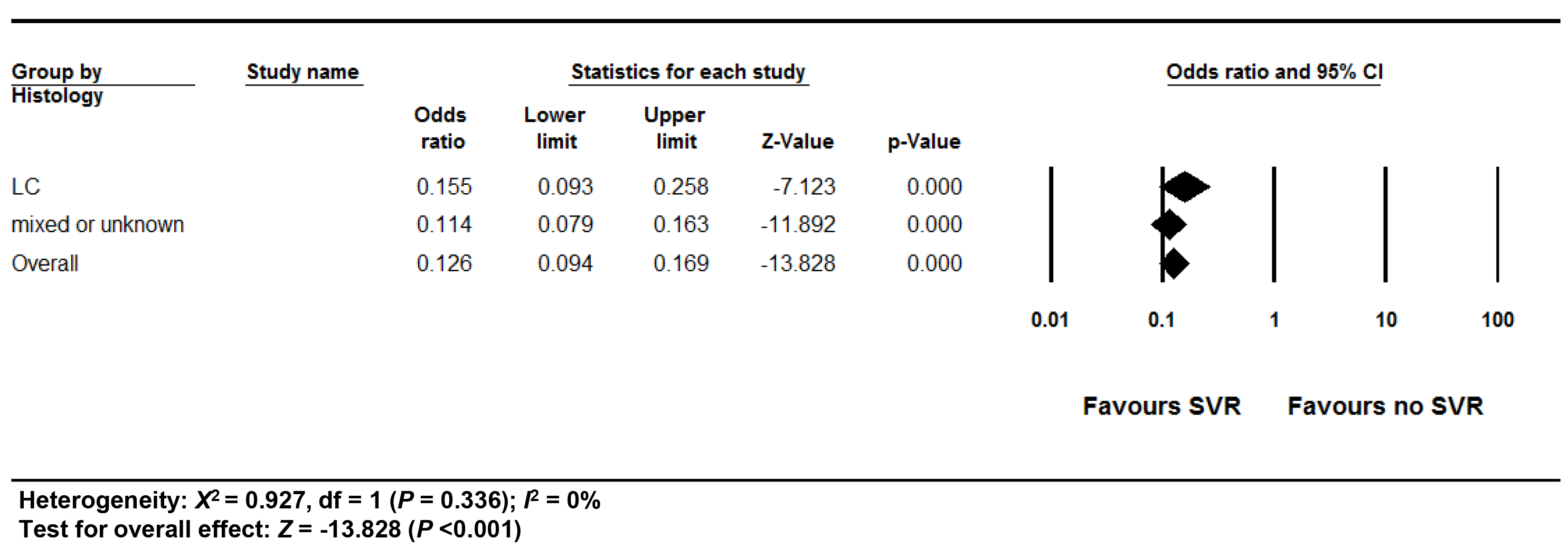
**

**
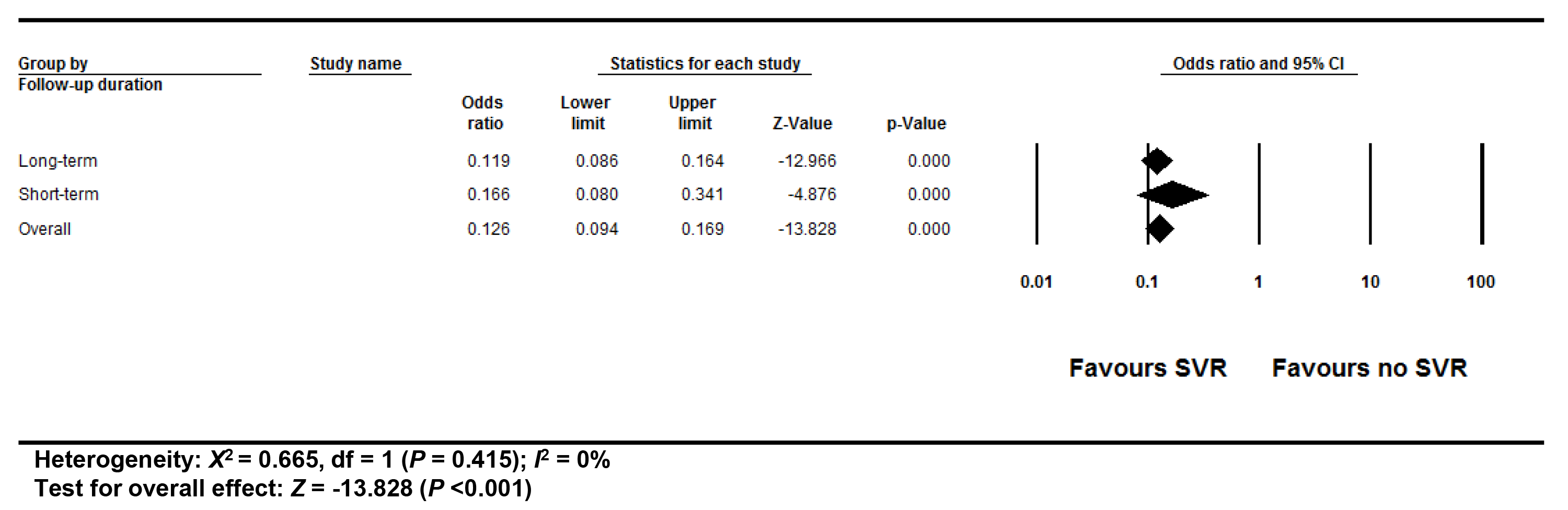
**

**
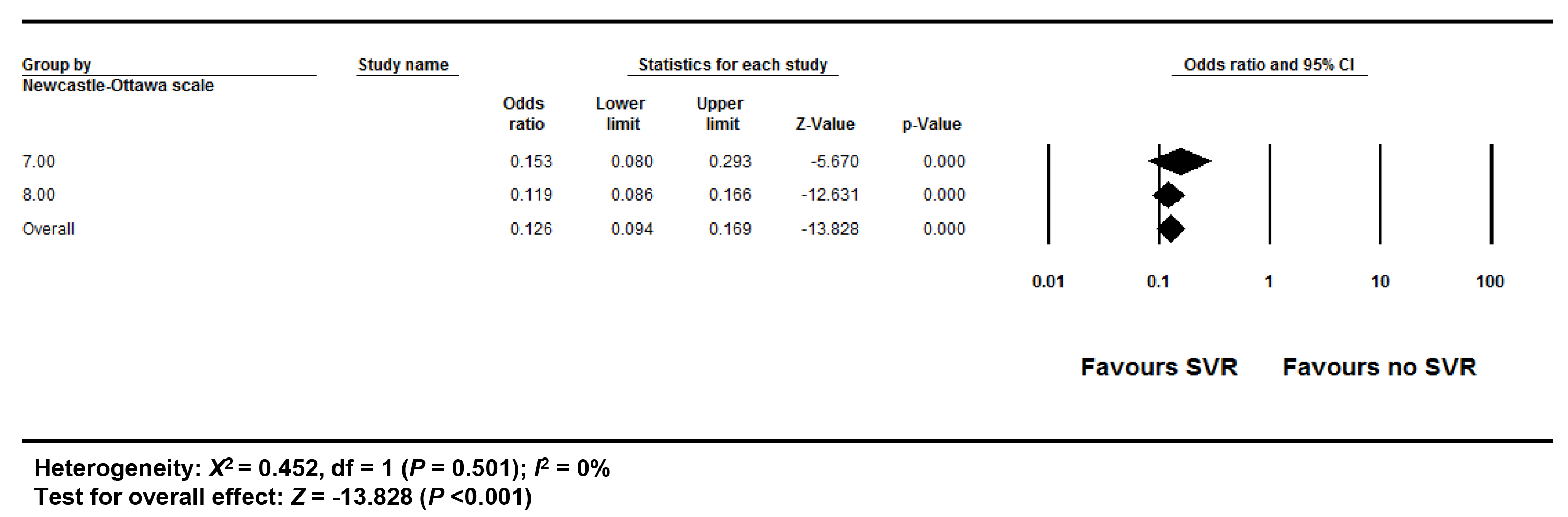
**

**
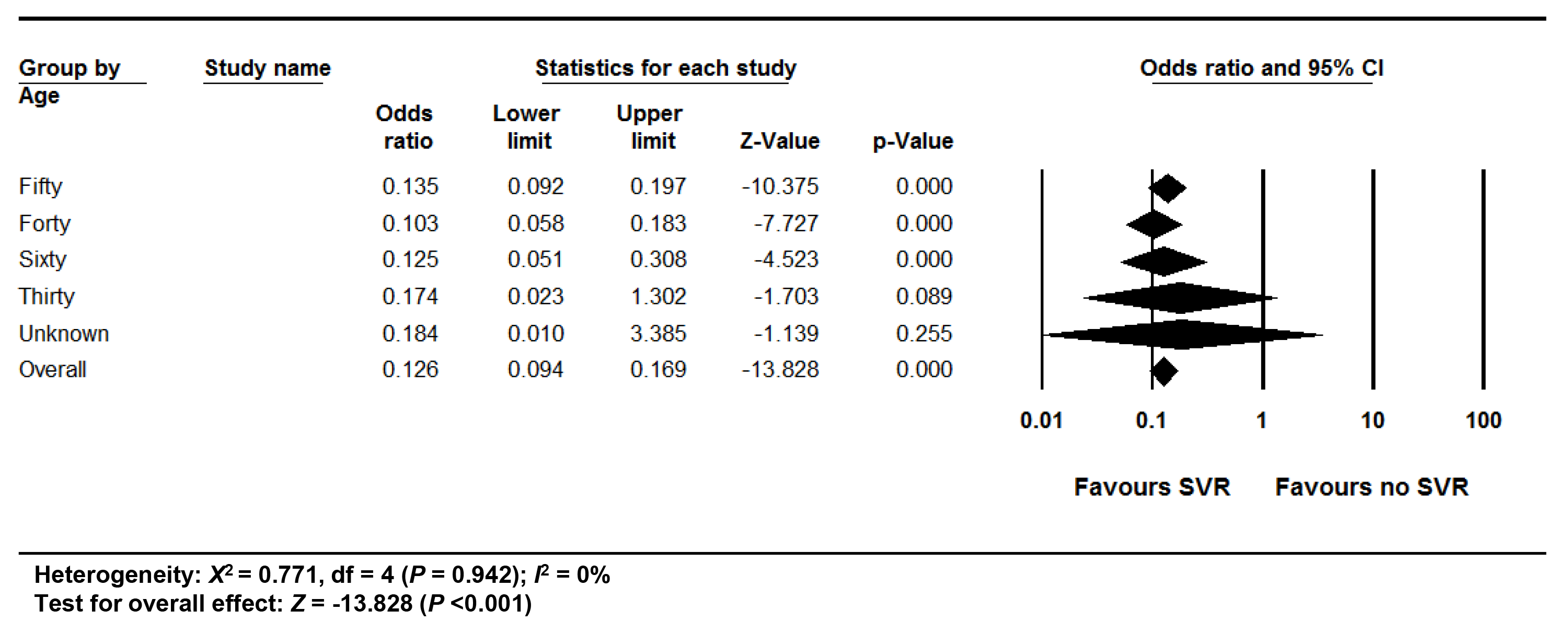
**

**
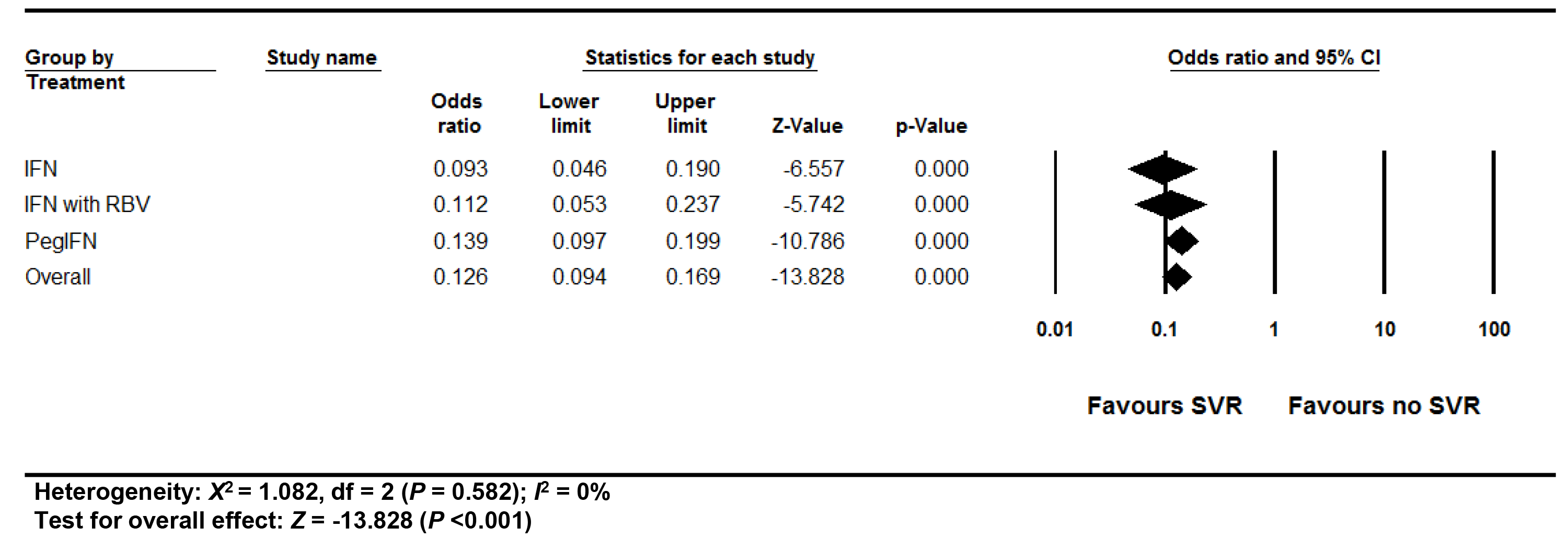
**

Diamond is the summary estimate from the pooled studies with 95% CI (Mixed effect model). SVR, sustained virologic response; CI, confidence interval.
