# Supplementary material for: Impact of antiviral therapy on hepatocellular carcinoma and mortality in patients with chronic hepatitis C: systematic review and meta-analysis
Source: BMC Gastroenterol. 2017 Apr 4;17:46. doi: 10.1186/s12876-017-0606-9 (PMC5379714; doi:10.1186/s12876-017-0606-9)
Supplement: Supplementary file 2 — Contains 6 tables including detailed meta-regression data of 6 study topics of this study. (DOC 79 kb) [file 12876_2017_606_MOESM2_ESM.doc]

**Additional file 2: Table S1** Meta-regression of studies for the efficacy of antiviral treatment on the development of HCC in patients with CHC

| **Modifier** | **Coefficient** | **Standard error** | ***P* value** |
| --- | --- | --- | --- |
| Study format | 0.361 | 0.278 | 0.193 |
| Nationality | 0.235 | 0.368 | 0.522 |
| Histology | 0.388 | 0.377 | 0.304 |
| Follow-up duration | -0.545 | 0.327 | 0.095 |
| NOS | NOS 8: 1.203  NOS 7: 0.501 | 0.565  0.561 | 0.033  0.372  Q: 7.24, df: 2, *P* = 0.027 |
| Age | Forty: 0.313  Thirty: 0.644  Unknown: 0.904 | 0.919  1.002  0.723 | 0.733  0.520  0.212  Q: 1.94, df: 3, *P* = 0.586 |
| Treatment | IFN with RBV: 0.654  PegIFN based treatment: 0.598 | 0.437  0.471 | 0.134  0.204  Q: 3.15, df: 2, *P* = 0.208 |

*HCC* hepatocellular carcinoma, *CHC* chronic hepatitis C, *NOS* Newcastle-Ottawa scale, *IFN* interferon, *PegIFN* pegylated interferon, *RBV* ribavirin

**Additional file 2: Table S2** Meta-regression of studies for the efficacy of antiviral treatment on all-cause mortality in patients with CHC

| **Modifier** | **Coefficient** | **Standard error** | ***P* value** |
| --- | --- | --- | --- |
| Study format | RCT: -1.493  Retrospective cohort study: -0.025 | 0.746  0.287 | 0.045  0.930  Q: 4.21, df: 2, *P* = 0.122 |
| Nationality | 0.431 | 0.246 | 0.080 |
| Histology | 0.081 | 0.346 | 0.815 |
| Follow-up duration | 0.424 | 0.465 | 0.361 |
| NOS | NOS 8: 0.127  RCT: -1.382 | 0.285  0.733 | 0.655  0.059  Q: 4.58, df: 2, *P* = 0.101 |
| Age | Forty: 0.684  Thirty: 0.359  Unknown: 0.230 | 0.596  0.581  0.616 | 0.251  0.537  0.708  Q: 1.70, df: 3, *P* = 0.637 |
| Treatment | IFN with RBV: 0.414  PegIFN based treatment: 0.326 | 0.276  0.355 | 0.134  0.359  Q: 2.45, df: 2, *P* = 0.294 |

*CHC* chronic hepatitis C, *NOS* Newcastle-Ottawa scale, *IFN* interferon, *PegIFN* pegylated interferon, *RBV* ribavirin

**Additional file 2 Table S3** Meta-regression of studies for the efficacy of antiviral treatment on liver-specific mortality in patients with CHC

| **Modifier** | **Coefficient** | **Standard error** | ***P* value** |
| --- | --- | --- | --- |
| Study format | -0.052 | 0.369 | 0.889 |
| Nationality | 0.365 | 0.367 | 0.321 |
| Histology | -0.066 | 0.395 | 0.868 |
| Follow-up duration | 1.140 | 0.542 | 0.036 |
| NOS | NOS 8: -0.659 | 0.302 | 0.029 |
| Age | Forty: 0.882  Thirty: 0.506  Unknown: 1.411 | 0.614  0.577  0.815 | 0.151  0.381  0.083  Q: 5.22, df: 3, *P* = 0.157 |
| Treatment | IFN with RBV: 0.165  PegIFN based treatment: 0.382 | 0.435  0.556 | 0.705  0.492  Q: 0.53, df: 2, *P* = 0.768 |

*CHC* chronic hepatitis C, *NOS* Newcastle-Ottawa scale, *IFN* interferon, *PegIFN* pegylated interferon, *RBV* ribavirin

**Additional file 2: Table S4** Meta-regression of studies for the efficacy of SVR on the development of HCC in patients with CHC

| **Modifier** | **Coefficient** | **Standard error** | ***P* value** |
| --- | --- | --- | --- |
| Study format | RCT: 1.213  Retrospective: -0.430 | 1.649  0.286 | 0.462  0.132  Q: 3.20, df: 2, *P* = 0.202 |
| Nationality | Saudi Arabia and Egypt: -0.136  Western: 0.196 | 1.549  0.224 | 0.930  0.384  Q: 0.78, df: 2, *P* = 0.677 |
| Histology | -0.461 | 0.254 | 0.070 |
| Follow-up duration | 0.073 | 0.220 | 0.739 |
| NOS | NOS 8: 0.538  NOS 7: 0.560  RCT: 2.052 | 0.255  0.271  1.628 | 0.035  0.039  0.208  Q: 6.09, df: 3, *P* = 0.108 |
| Age | Sixty: 0.805  Forty: -0.087  Thirty: 0.282  Unknown: 0.281 | 0.373  0.294  1.080  1.120 | 0.031  0.767  0.794  0.802  Q: 5.09, df: 4, *P* = 0.279 |
| Treatment | IFN with RBV: -0.541  PegIFN based treatment: -0.317 | 0.323  0.270 | 0.240  0.240  Q: 2.84, df: 2, *P* = 0.242 |

*SVR* sustained virologic response, *HCC* hepatocellular carcinoma, *CHC* chronic hepatitis C, *NOS* Newcastle-Ottawa scale, *IFN* interferon, *PegIFN* pegylated interferon, *RBV* ribavirin

**Additional file 2: Table S5** Meta-regression of studies for the efficacy of SVR on all-cause mortality in patients with CHC

| **Modifier** | **Coefficient** | **Standard error** | ***P* value** |
| --- | --- | --- | --- |
| Study format | 0.231 | 0.437 | 0.598 |
| Nationality | Saudi Arabia and Egypt: -1.087  Western: 0.096 | 1.515  0.252 | 0.473  0.704  Q: 0.72, df: 2, *P* = 0.697 |
| Histology | -0.093 | 0.395 | 0.814 |
| Follow-up duration | 0.223 | 0.246 | 0.364 |
| NOS | NOS 8: -0.540  NOS 7: -0.544 | 0.209  0.322 | 0.010  0.091  Q: 7.03, df: 2, *P* = 0.030 |
| Age | Sixty: 0.462  Forty: -0.291  Unknown: -0.933 | 0.384  0.295  1.504 | 0.229  0.325  0.535  Q: 3.39, df: 3, *P* = 0.335 |
| Treatment | IFN with RBV: 0.357  PegIFN based treatment: 0.247 | 0.352  0.304 | 0.310  0.417  Q: 1.10, df: 2, *P* = 0.578 |

*SVR* sustained virologic response, *CHC* chronic hepatitis C, *NOS* Newcastle-Ottawa scale, *IFN* interferon, *PegIFN* pegylated interferon, *RBV* ribavirin

**Additional file 2: Table S6** Meta-regression of studies for the efficacy of SVR on liver-specific mortality in patients with CHC

| **Modifier** | **Coefficient** | **Standard error** | ***P* value** |
| --- | --- | --- | --- |
| Study format | -0.297 | 0.334 | 0.375 |
| Nationality | 0.403 | 0.341 | 0.238 |
| Histology | -0.308 | 0.320 | 0.336 |
| Follow-up duration | 0.329 | 0.404 | 0.415 |
| NOS | -0.250 | 0.371 | 0.501 |
| Age | Sixty: -0.071  Forty: -0.272  Thirty: 0.258  Unknown: 0.312 | 0.498  0.352  1.044  1.498 | 0.886  0.439  0.805  0.835  Q: 0.77, df: 4, *P* = 0.942 |
| Treatment | IFN with RBV: 0.184  PegIFN based treatment: 0.401 | 0.525  0.405 | 0.726  0.323  Q: 1.08, df: 2, *P* = 0.582 |

*SVR* sustained virologic response, *CHC* chronic hepatitis C, *NOS* Newcastle-Ottawa scale, *IFN* interferon, *PegIFN* pegylated interferon, *RBV* ribavirin
